# Efficacy of Liposomal Bupivacaine and Bupivacaine Hydrochloride vs Bupivacaine Hydrochloride Alone as a Periarticular Anesthetic for Patients Undergoing Knee Replacement: A Randomized Clinical Trial

**DOI:** 10.1001/jamasurg.2022.0713

**Published:** 2022-04-06

**Authors:** Thomas W. Hamilton, Ruth Knight, Jamie R. Stokes, Ines Rombach, Cushla Cooper, Loretta Davies, Susan J. Dutton, Karen L. Barker, Jonathan Cook, Sarah E. Lamb, David W. Murray, Lisa Poulton, Ariel Wang, Louise H. Strickland, Bernard H. Van Duren, Jose Leal, David Beard, Hemant G. Pandit

**Affiliations:** 1 Oxford Orthopaedic Engineering Centre, Nuffield Department of Orthopaedics Rheumatology and Musculoskeletal Sciences, University of Oxford, Oxford, United Kingdom; 2 Oxford Clinical Trials Research Unit, Centre for Statistics in Medicine, Nuffield Department of Orthopaedics, Rheumatology and Musculoskeletal Sciences, University of Oxford, Oxford, United Kingdom; 3 Health Economics Research Centre, Nuffield Department of Population Health, University of Oxford, Oxford, United Kingdom; 4 Surgical Interventional Trials Unit, Nuffield Department of Orthopaedics, Rheumatology and Musculoskeletal Sciences, University of Oxford, Oxford, United Kingdom; 5 National Institute for Health Research–Biomedical Research Unit, Nuffield Department of Orthopaedics, Rheumatology and Musculoskeletal Sciences, University of Oxford, Oxford, United Kingdom; 6 Physiotherapy Research Unit, Nuffield Orthopaedic Centre, Oxford University Hospitals, NHS (National Health Service) Foundation Trust, Oxford, United Kingdom; 7 Leeds Institute of Rheumatic and Musculoskeletal Medicine, Chapel Allerton Hospital, University of Leeds, Leeds, United Kingdom

## Abstract

**Importance:**

More than half of patients who undergo knee replacement surgery report substantial acute postoperative pain.

**Objective:**

To evaluate the efficacy and cost-effectiveness of periarticular liposomal bupivacaine for recovery and pain management after knee replacement.

**Design, Setting, and Participants:**

This multicenter, patient-blinded, pragmatic, randomized clinical superiority trial involved 533 participants at 11 institutions within the National Health Service in England. Adults undergoing primary unilateral knee replacement for symptomatic end-stage osteoarthritis were enrolled between March 29, 2018, and February 29, 2020, and followed up for 1 year after surgery. Follow-up was completed March 1, 2021. A per-protocol analysis for each coprimary outcome was performed in addition to the main intention-to-treat analysis.

**Interventions:**

Two hundred sixty-six milligrams of liposomal bupivacaine admixed with 100 mg of bupivacaine hydrochloride compared with 100 mg of bupivacaine hydrochloride alone (control) administered by periarticular injection at the time of surgery.

**Main Outcome and Measures:**

The coprimary outcomes were Quality of Recovery 40 (QoR-40) score at 72 hours and pain visual analog scale (VAS) score area under the curve (AUC) from 6 to 72 hours. Secondary outcomes included QoR-40 and mean pain VAS at days 0 (evening of surgery), 1, 2, and 3; cumulative opioid consumption for 72 hours; functional outcomes and quality of life at 6 weeks, 6 months, and 1 year; and cost-effectiveness for 1 year. Adverse events and serious adverse events up to 12 months after randomization were also assessed.

**Results:**

Among the 533 participants included in the analysis, the mean (SD) age was 69.0 (9.7) years; 287 patients were women (53.8%) and 246 were men (46.2%). Baseline characteristics were balanced between study groups. There was no difference between the liposomal bupivacaine and control groups in QoR-40 score at 72 hours (adjusted mean difference, 0.54 [97.5% CI, −2.05 to 3.13]; *P* = .64) or the pain VAS score AUC at 6 to 72 hours (−21.5 [97.5% CI, −46.8 to 3.8]; *P* = .06). Analyses of pain VAS and QoR-40 scores demonstrated only 1 statistically significant difference, with the liposomal bupivacaine arm having lower pain scores the evening of surgery (adjusted difference −0.54 [97.5% CI, −1.07 to −0.02]; *P* = .02). No difference in cumulative opioid consumption and functional outcomes was detected. Liposomal bupivacaine was not cost-effective compared with the control treatment. No difference in adverse or serious adverse events was found between the liposomal bupivacaine and control groups.

**Conclusions and Relevance:**

This study found no difference in postoperative recovery or pain associated with the use of periarticular liposomal bupivacaine compared with bupivacaine hydrochloride alone in patients who underwent knee replacement surgery.

**Trial Registration:**

isrctn.com Identifier: ISRCTN54191675

## Key Points

**Question:**

Among patients undergoing knee replacement surgery, does liposomal bupivacaine and bupivacaine hydrochloride administered at the surgical site improve postoperative recovery at 72 hours and postoperative pain from 6 to 72 hours compared with bupivacaine hydrochloride alone?

**Findings:**

In this randomized clinical trial of 533 patients undergoing knee replacement surgery, no difference in the coprimary outcomes of Quality of Recovery 40 score at 72 hours or pain visual analog scale score area under the curve from 6 to 72 hours was detected between patients receiving liposomal bupivacaine and bupivacaine hydrochloride and those receiving bupivacaine hydrochloride alone. In addition, liposomal bupivacaine was not found to be cost-effective.

**Meaning:**

This study found that liposomal bupivacaine did not improve postoperative recovery or pain compared with bupivacaine hydrochloride alone among patients undergoing knee replacement surgery.

## Teaser

This randomized clinical trial assesses the efficacy and cost-effectiveness of liposomal bupivacaine plus bupivacaine hydrochloride compared with bupivacaine hydrochloride alone for postoperative recovery and pain management among patients undergoing knee replacement.

## Introduction

Knee replacement is a highly successful operation for patients with symptomatic advanced arthritis refractory to nonoperative treatment; however, recovery from surgery can be painful despite multimodal opiate-sparing techniques.^1,2^ Postoperative pain is detrimental to the patient experience and has been reported to be associated with an increased morbidity and mortality and may be associated with long-term outcomes.^3,4,5^ The ideal analgesic would be one that is locally delivered, avoiding systemic adverse effects, and that provides a long-lasting sensory but not motor block. Liposomal bupivacaine (Exparel [Pacira Pharmaceuticals Inc]) is a novel liposome-encapsulated local anesthetic developed to improve postoperative analgesia and reduce the need for supplementary opiate analgesia.^6^ In the knee, liposomal bupivacaine is licensed for single-dose periarticular local infiltration at the time of surgery.

Current evidence on the effectiveness of liposomal bupivacaine is inconclusive owing to small study size and nonstandardized comparators.^7^ The present study was a large randomized clinical trial assessing the clinical efficacy and cost-effectiveness of liposomal bupivacaine plus bupivacaine hydrochloride compared with bupivacaine hydrochloride alone (control condition) for postoperative recovery and pain after knee replacement to guide best clinical practice. We sought to assess the null hypothesis that no true difference in postoperative recovery and pain exists after knee replacement between liposomal bupivacaine plus bupivacaine hydrochloride compared with bupivacaine hydrochloride alone when administered by periarticular infiltration at the time of surgery.

## Methods

### Design

This trial was a multicenter, randomized, patient-blinded, active comparator-controlled, superiority clinical trial conducted across 11 National Health Service institutions in the UK. A summary of the study protocol has been published previously and is available in Supplement 1.^8,9^ The study followed the Consolidated Standards of Reporting Trials (CONSORT) and Consolidated Health Economic Evaluation Reporting Standards (CHEERS) reporting guidelines. The trial was approved by the National Research Ethics Service, Oxfordshire Research Ethics Committee C in 2017, with written informed consent obtained from all participants before their involvement in the study.

### Participants

Eligible participants were adults 18 years or older with American Society of Anaesthesiologists grades 1 to 3 physical classification status inclusive who underwent unilateral primary total knee preplacement (TKR) or unicompartmental knee replacement (UKR) for end-stage osteoarthritis. Patients were excluded if they had rheumatoid arthritis, an allergy or intolerance to amide-type local anesthetics, objective evidence of nerve damage in the affected lower limb, or contralateral knee replacement within the 12 months before randomization or had participated in another research trial involving an investigational medicinal product in the 6 months before randomization. A list of the participating centers is available in eAppendix 1 in Supplement 2; a list of members of the Data and Safety Monitoring Committee is available in eAppendix 2 in Supplement 2.

### Randomization

Patients were randomized 1:1 to the intervention (liposomal bupivacaine plus bupivacaine hydrochloride) or control (bupivacaine hydrochloride) arms using a secure online system. Randomization was stratified according to recruitment site and type of surgery (TKR or UKR). Treatment group numbers were balanced using random permuted blocks of sizes 2, 4, and 6.

### Interventions

All patients underwent knee replacement with the surgical technique, implants used, and alignment philosophy in line with surgeons’ usual practice. Patients randomized to the intervention arm received 266 mg of liposomal bupivacaine admixed with 100 mg of bupivacaine hydrochloride without epinephrine. Those randomized to the control arm received 100 mg of bupivacaine hydrochloride without epinephrine. In both groups the volume was expanded to 120 mL with normal saline. Liposomal bupivacaine and control drugs were administered via a standardized periarticular injection technique with a reference guide provided in each operating theater. Preoperative, intraoperative, and postoperative analgesia regimens and management were in line with local protocols and did not differ based on patient randomization.

### Outcomes

The coprimary outcome measures were the Quality of Recovery 40 (QoR-40) score^10^ at 72 hours and pain visual analog scale (VAS) score from 6 to 72 hours after surgery.^11^ Predefined secondary outcomes included QoR-40 and VAS scores at days 0 (evening of surgery), 1, 2, and 3; cumulative opioid consumption; functional outcomes measured using the Oxford Knee Score and American Knee Society Score; quality of life measured using the 5-level EQ-5D (EQ-5D-5L); use of health care resources; and complications measured using the Clavien-Dindo classification.^12,13,14,15^

### Statistical Analysis

The full statistical analysis plan has previously been published and can be accessed in Supplement 1.^9^ The sample size for the trial was 500 patients. A total of 240 patients per arm were required to detect a 5-point difference in global QoR-40 scores at *P* = .025 significance level with 90% power, assuming an SD of 15.5. Allowing a 4% loss to follow-up increased this sample size to 500 patients overall. This sample size was also sufficient to detect a standardized difference of 33% between treatment groups in cumulative pain score calculated as the area under the curve (AUC) from 6 to 72 hours after surgery, with *P* = .025 indicating statistical significance and allowing for 10% loss to follow-up.

The QoR-40 scores at 72 hours after surgery in the treatment groups were compared using a mixed-effects linear regression model adjusting for type of surgery, baseline QoR-40 scores, age, and sex as fixed effects and recruitment site as a random effect. Cumulative pain scores were compared between the treatment groups using parameters from a repeated-measures mixed-effects linear regression model to calculate the summary measures AUC from 6 to 72 hours after surgery.^11^ Continuous secondary outcomes were analyzed using multilevel mixed-effects linear regression models; categorical secondary outcomes were analyzed using multilevel mixed-effects logistic regression models.

Sensitivity analyses to assess the impact of missing data were conducted on both coprimary outcomes. A per-protocol analysis for each coprimary outcome was performed in addition to the main intention-to-treat (ITT) analysis. The primary outcome analyses were performed with a 2-sided *P* = .025 indicating statistical significance to adjust for multiplicity associated with the coprimary outcomes, with all other analyses performed with a 2-sided *P* < .05 indicating statistical significance.

### Health Economic Analysis

Patients were followed up for 1 year, until March 1, 2021. We conducted a within-trial cost-utility analysis during the follow-up, adopting the perspective of the National Health Service in the UK and personal social services. Details of the analysis are available in the eMethods and eTables 1 to 5 in Supplement 2. Briefly, we estimated quality-adjusted life-years (QALYs) and derived total costs (price year 2019) from resources used during the index procedure (knee replacement surgery and associated hospital stay,^16^ opioids, and liposomal bupivacaine injection), self-reported use of health care and social services resources, and hospital readmissions obtained from trial sites. Missing baseline and follow-up data were handled by mean and multiple imputation, respectively.

Differences in total health care costs and QALYs were estimated using linear regression models adjusted for randomized treatment allocation, type of surgery performed (TKR vs UKR), and baseline utility (QALYs only); robust standard errors were used to account for clustering by site. We estimated the incremental cost-effectiveness ratio by dividing the mean cost difference by the mean QALY difference. We assessed the joint uncertainty around incremental total costs, QALYs, and cost-effectiveness via bootstrapping, used to calculate the probability that liposomal bupivacaine is cost-effective compared with bupivacaine hydrochloride alone at a willingness-to-pay threshold of £20 000 (US $26 400)/QALY gained. An intervention was judged to be cost-effective if the incremental cost-effectiveness ratio was less than £20 000 (US $26 400)/QALY gained. A cost-effectiveness acceptability curve was used to show the probability that liposomal bupivacaine is cost-effective at different willingness-to-pay threshold values to £60 000 (US $79 200)/QALY.

In sensitivity analyses, we considered a wider perspective and included patient costs (private contacts, equipment, and home changes) and productivity losses. To facilitate comprehension of the cost data, we converted UK health care costs to US dollars using 2019 power purchasing parities for gross domestic product (1.45), 2019 exchange rates (1.28), and 2017 power purchasing parities for health (1.33) as sensitivity analysis. Because the economic results in the UK setting cannot be applied directly to the US setting, we did not estimate incremental cost-effectiveness ratios or cost-effectiveness acceptability curves in US dollars. All analyses were undertaken using Stata, version 15 (StataCorp LLC).

## Results

### Participants

**Figure 1.**
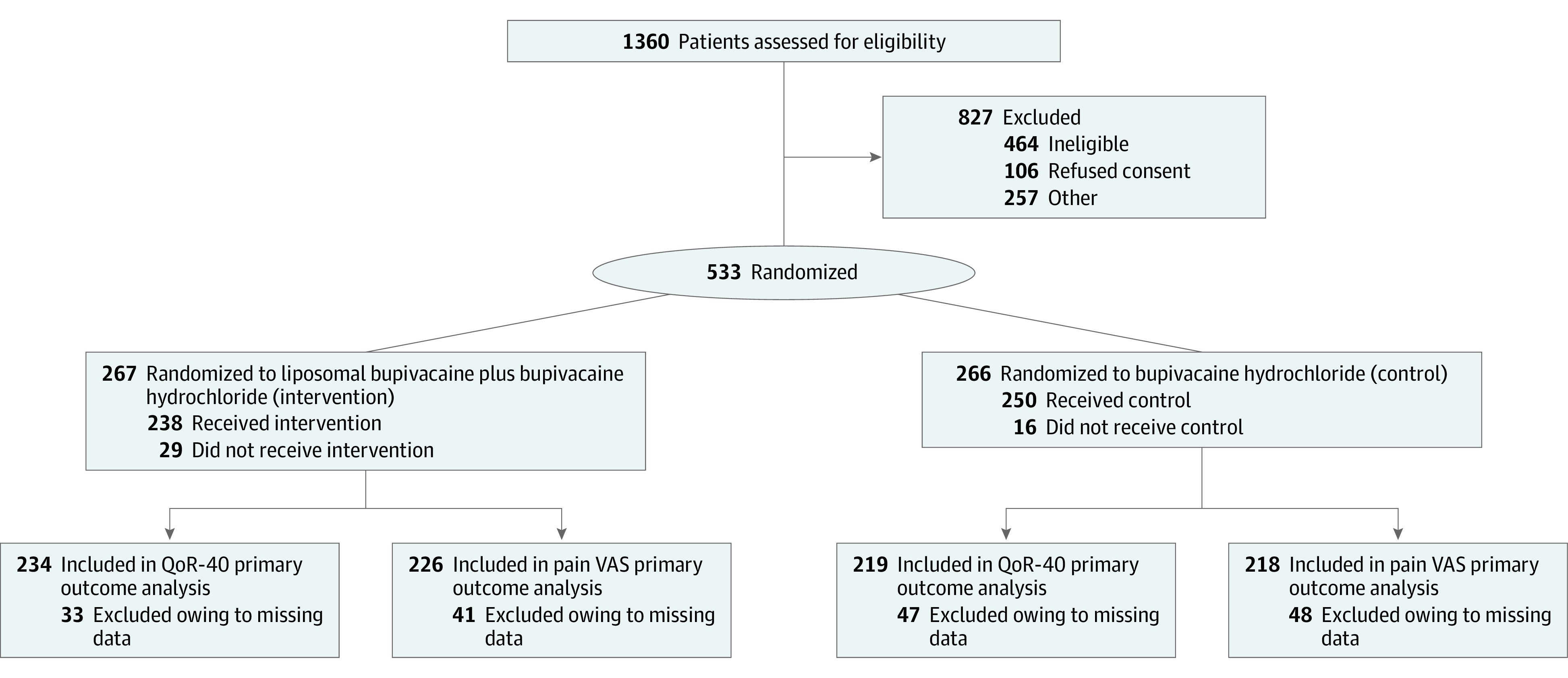
CONSORT Flow Diagram of Patient Enrollment, Randomization, and Follow-up QoR-40 indicates Quality of Recovery 40; VAS, visual analog scale.

From March 29, 2018, to February 29, 2020, 1360 participants were assessed for eligibility for the trial. A total of 827 participants were excluded before randomization, with the remaining 533 participants randomized into 2 groups. A CONSORT flow diagram is provided in Figure 1 and eTable 6 in Supplement 2 shows stratification factors at baseline. The mean (SD) age of participants was 69.0 (9.7) years; 287 patients were women (53.8%) and 246 were men (46.2%). Race and ethnicity data were not collected. The mean (SD) body mass index (calculated as weight in kilograms divided by height in meters squared) was 31.8 (6.1). Baseline characteristics were well balanced between groups (Table 1 and eTable 8 in Supplement 2).

**Table 1.** Baseline Characteristics of Study Population

Characteristic	Patient group		
	Intervention (n = 267)	Control (n = 266)	All (n = 533)
Age			
No. of patients	267	266	533
Mean (SD) [range], y	68.9 (10.1) [39.4-91.4]	69.0 (9.3) [43.5-90.5]	69.0 (9.7) [39.4-91.4]
BMI			
No. of patients	263	263	526
Mean (SD) [range]	32.0 (6.4) [9.2-49.7]	31.6 (5.9) [10.0-53.9]	31.8 (6.1) [9.2-53.9]
Sex, No. (%)			
Men	116/267 (43.4)	130/266 (48.9)	246/533 (46.2)
Women	151/267 (56.5)	136/266 (51.1)	287/533 (53.8)
Knee, No. (%)^a^			
Left	115/267 (43.1)	114/266 (42.9)	229 (43.0)
Right	149/267 (55.8)	151/266 (56.8)	300 (56.3)
ASA grade, No. (%)^b^			
1	18/267 (6.7)	17/266 (6.4)	35/533 (6.6)
2	187/267 (70.0)	174/266 (65.4)	361/533 (67.7)
3	52/267 (19.5)	64/266 (24.1)	116/533 (21.8)

Abbreviations: ASA, American Society of Anesthesiologists; BMI, body mass index (calculated as weight in kilograms divided by height in meters squared).

^a^ Data were missing for 4 participants (3 in the intervention group, 1 in the control group).

^b^ Data were missing for 21 participants (10 in the intervention group, 11 in the control group).

### Procedural Demographics

Among the 514 patients with available data, 461 (89.7%) received a TKR and 53 (10.3%) received a UKR. The type of surgery, anesthetic technique used, and time in the operating theater were similar between groups (eTable 9 in Supplement 2). Data on adherence to the administration technique for liposomal bupivacaine were available for 1483 of the 1548 syringes administered (95.8%), with 1462 syringes (98.6%) administered in line with the standardized protocol. Data on the difficulty of administration of liposomal bupivacaine were available for 246 cases and recorded as easy in 193 (78.5%), moderate in 47 (19.1%), and difficult in 6 (2.4%).

### Withdrawals and ITT and Per-Protocol Populations

Similar numbers of participants withdrew from the treatment groups, with most (16 of 23 [69.6%]) withdrawing before receiving their knee surgery (eTable 10 in Supplement 2). No patients were unblinded before the end of the trial.

The numbers of participants included in the ITT analysis are reported in the relevant analysis tables (eTables 7, 9-12, and 15 in Supplement 2). Participants were defined as being in the per-protocol population if they received their randomized treatment as planned and had at least 1 of the coprimary outcomes available for analysis, with details available in eTable 11 in Supplement 2.

### Coprimary Outcomes

For the ITT analysis, the adjusted mean difference for QoR-40 scores was 0.54 (97.5% CI, −2.05 to 3.13; *P* = .64) and the adjusted mean difference for pain VAS score AUC at 6 to 72 hours was −21.5 (97.5% CI, −46.8 to 3.8; *P* = .06). Based on the significance level of .025, there was no statistical evidence of a difference between the intervention and control groups with regard to either QoR-40 score at 72 hours after surgery or pain VAS score AUC from 6 to 72 hours after surgery (eTable 12 in Supplement 2). Findings were consistent for the per-protocol population (eTable 13 in Supplement 2) and the different sites (eFigure 1 in Supplement 2).

### Secondary Outcomes

**Figure 2.**
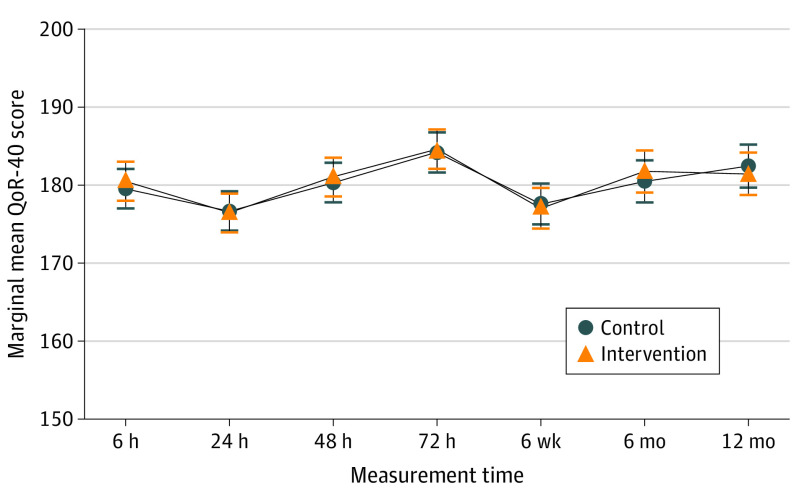
Marginal Mean Quality of Recovery 40 (QoR-40) Scores at Each Time Point Error bars indicate 97.5% CIs. For numbers analyzed at each time point, see eTable 14 in Supplement 2.

**Figure 3.**
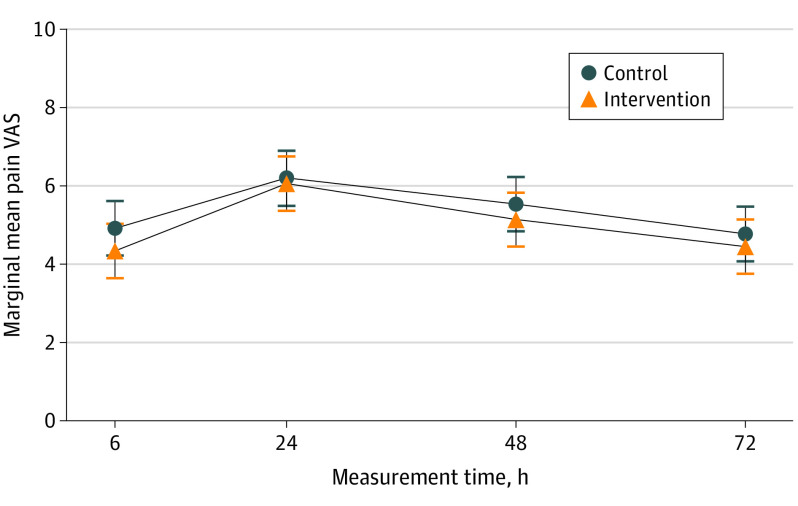
Marginal Mean Pain Visual Analog Scale (VAS) Scores at Each Time Point Error bars indicate 97.5% CIs. For numbers analyzed at each time point, see eTable 14 in Supplement 2.

Using ITT analysis, no statistically significant difference was detected for the QoR-40 score at any follow-up time point (Figure 2 and eTable 14 in Supplement 2). For pain VAS score at 6 hours after surgery, a statistically significant difference based on the threshold significance level of .025 was detected in favor of liposomal bupivacaine (adjusted difference, −0.54 [97.5% CI, −1.07 to −0.02]; *P* = .02). All other time points showed no evidence of statistically significant difference for the pain VAS scores (Figure 3 and eTable 14 in Supplement 2).

Cumulative mean (SD) best-case oral morphine equivalent opioid consumption from days 0 to 3 in the liposomal bupivacaine arm was 126.5 (88.9) mg and 127.4 (132.6) mg in the control arm. No statistically significant difference in best-case cumulative opiate use (adjusted difference, −3.06 mg [95% CI, −22.32 to 16.19 mg]; *P* = .76) or worst-case cumulative opiate use (adjusted difference, −6.83 mg [95% CI, −26.09 to −12.42 mg]; *P* = .49) was detected between treatment arms (eTable 15 in Supplement 2).

Analysis of Oxford Knee and American Knee Society Expectations, Satisfaction, and Function scores at 6 weeks and 6 and 12 months identified no statistically significant differences between treatment groups (eTable 16 in Supplement 2). No statistically significant difference in fitness for discharge was observed between the treatment groups at any time point (eTable 17 in Supplement 2).

### Adverse Events

Clavien-Dindo classification of inpatient and surgical complications was similar between groups (eTable 18 in Supplement 2). The number of adverse events and serious adverse events were balanced between treatment arms (eTable 19 in Supplement 2). Three deaths occurred in the intervention arm, none of which were related to the trial treatment (eTable 19 in Supplement 2).

### Sensitivity Analysis

The impact of missing data on the coprimary outcomes was explored via sensitivity analyses. These analyses did not alter the findings of the main analysis (eFigure 2 and eTable 20 in Supplement 2).

### Health Economic Analysis

Availability of EQ-5D-5L and use of health care resources data ranged from 96% at baseline to 70% at the 1-year follow-up (eTable 21 in Supplement 2). Liposomal bupivacaine was dominated by the control intervention (ie, it was less effective; adjusted difference for 1 year, −0.005 QALYs [95% CI, −0.048 to 0.038 QALYs]) (eTable 22 in Supplement 2) and more costly (adjusted difference, £22 [US $29] [95% CI, −£410 (US $540) to £455 (US $599)]) (eTables 23 and 24 in Supplement 2), although the differences were not significant. Furthermore, no significant differences were observed for any of the costing components, with the exception of liposomal bupivacaine costs, which were only incurred in the liposomal bupivacaine arm (eTable 25 and eFigure 3 in Supplement 2). The probability that liposomal bupivacaine was cost-effective was 37% at a willingness-to-pay threshold of £20 000 (US $26 400)/QALY gained (eTable 20 and eFigure 4 in Supplement 2). Adopting a wider perspective (societal), liposomal bupivacaine remained dominated by the control arm and, hence, was not cost-effective for various willingness-to-pay thresholds (Table 2 and eFigure 4 in Supplement 2). Health care costs converted to US dollars are provided in eTable 26 in Supplement 2. Here, the nonsignificant differences in total health care costs between the liposomal bupivacaine intervention arm and the control arm varied from $28.7 using the exchange rate to $32.5 using power purchasing parities for gross domestic product.

**Table 2.** Life-Years, QALYs, Health Care Costs, and Cost-effectiveness for the Base-Case Analysis at 1 Year After Multiple Imputation

	Mean (SE)		Mean difference (95% CI)
	Intervention	Control	
No. of patients	267	266	NA
Life-years^a^	1.0 (0.0)	1.0 (0.0)	0.0 (0.0 to 0.0)
QALYs^b^	0.689 (0.187)	0.698 (0.164)	−0.005 (−0.048 to 0.038)
Costs, £^c^^,^^d^			
Total NHS and PSS (including intervention)	6779.8 (112.0)	6757.2 (147.7)	22.4 (−410.0 to 454.9)
Liposomal bupivacaine	224.6 (3.8)	0.0 (0.0)	224.6 (211.6 to 237.6)
Total non-NHS	1068.1 (195.1)	1012.1 (198.6)	56.0 (−515.4 to 627.3)
Total societal	7847.9 (227.5)	7769.3 (256.4)	78.4 (−690.5 to 847.3)
Incremental cost-effectiveness ratios^e^			
Total NHS and PSS costs	NA	NA	Dominated^f^
Total societal costs	NA	NA	Dominated^f^

Abbreviations: NA, not applicable; NHS, National Health Service; PSS, personal and social services; QALY, quality-adjusted life year.

^a^ All patients in the intervention arm were followed up for 1 year except for 3 patients who died before the end of follow-up.

^b^ Differences derived from linear regression model of each treatment allocation against each outcome adjusted for recruitment site and, for QALYs, baseline utility score.

^c^ Differences derived from unadjusted linear regression models.

^d^ To convert to US dollars, multiply by 1.32.

^e^ Probability of cost-effectiveness at willingness-to-pay threshold of £20 000 (US $26 400)/QALY (NHS and PSS perspective) was 37%.

^f^ Indicates intervention is less effective but more costly than control.

## Discussion

Our findings show no clear benefit to the use of periarticular liposomal bupivacaine plus bupivacaine hydrochloride compared with bupivacaine hydrochloride alone in the treatment of postoperative pain after knee replacement surgery. No significant difference in QoR-40 score at 72 hours or pain VAS score AUC from 6 to 72 hours was detected between treatment groups. In addition, analysis of secondary outcomes, including QoR-40 scores, cumulative opioid consumption on days 0, 1, 2, and 3, and patient-reported functional outcomes at 6 weeks, 6 months, or 1 year found no significant differences in these outcomes. Aside from pain VAS score on the evening of surgery (day 0), where the liposomal bupivacaine intervention group was found to have lower scores (adjusted difference, −0.54 [97.5% CI, −1.07 to −0.02]; *P* = .02), there was no significant difference in outcomes at other time points. This difference on day 0 was not viewed to be of a clinically relevant magnitude, nor at a clinically relevant time point with respect to the mechanism of action of the investigational medicinal product. Cost-utility analysis found periarticular liposomal bupivacaine with bupivacaine hydrochloride not to be cost-effective compared with bupivacaine hydrochloride alone after knee replacement surgery.

We are aware of 17 previous randomized clinical trials^17,18,19,20,21,22,23,24,25,26,27,28,29,30,31,32,33^ that have compared periarticular infiltration with 266 mg of liposomal bupivacaine against periarticular infiltration with either bupivacaine hydrochloride or ropivacaine hydrochloride (eTable 27 in Supplement 2). Most were small trials with a heterogeneous range of interventions and controls. Only 1 study, the PILLAR trial,^28^ reported significantly better cumulative pain scores and lower opioid consumption across the duration of the study (12 to 48 hours), and 3 additional studies^22,23,31^ reported lower pain scores at isolated time points within the analysis, with only 1 study^22^ demonstrating concurrently lower opioid intake at this time point. All other studies^17,18,19,20,21,24,25,26,27,29,30,32,33^ found no difference in mean pain score or opioid consumption at the time points assessed.

In the PILLAR trial,^28^ opioid medication was restricted postoperatively, and staff and patients were educated about the risks associated with opioids, with opioids only given on request for breakthrough pain. Under these circumstances, the investigators found the opioid consumption in the liposomal bupivacaine arm (20.9 [8.7] mg) to be markedly lower than others reported in the literature, including the present study. Theoretically, high baseline levels of opioids may mask any effect of the treatment intervention; however, we identified 4 further trials with a restrictive opioid policy,^17,23,26,29^ but without patient and staff education, with none demonstrating any difference in pain scores or opioid consumption between the liposomal bupivacaine and control groups. In our trial, 456 of 459 participants (99.3%) received opioids, which is similar to the PILLAR trial,^28^ in which 90% of patients received opioids in the intervention group and 100% in the control group; however, in only 1 center, the mean cumulative opioid consumption was similar to that reported in the PILLAR trial (mean [SD], 24.6 [8.1] mg [n = 8]), with the remaining centers having a value of a minimum of 3-fold higher.

A balance must be found between preemptive and reactive prescribing. Current surgical practice in the UK is to prescribe preemptive opioid-based analgesia; therefore, the UK practice must be regarded as opioid-sparing, not opioid-free. In this setting, we found no benefit to the use of periarticular liposomal bupivacaine. Whether liposomal bupivacaine has a role in the setting of opioid-free knee replacement remains unknown, but at present opioid-free surgery is rare and not a reality for most patients.

Other potential reasons why a treatment effect was not seen with liposomal bupivacaine may relate to the source of pain as well as the pharmacokinetics of liposomal bupivacaine in knee replacement. Several studies^34,35^ have investigated the roles of continuous intra-articular infiltration after knee replacement. Although these studies appear to provide improved pain control, compared with single-shot local anesthetic infiltration, it must be noted that the pain scores remain above 0 and typically range from 2 to 4 of 10 from 12 to 72 hours after surgery, indicating that not all pain can be targeted by intra-articular local anesthetic injection. Another reason may relate to the pharmacokinetic profile of liposomal bupivacaine. Liposomal bupivacaine exhibits a bimodal, dose-related release profile with an initial peak release within 1 hour of administration related to extra liposomal bupivacaine, followed by a further peak 12 to 36 hours later, related to release from the liposomes.^17,36^ The rate at which liposomes release bupivacaine has been proposed to be related to the vascularity of the surrounding tissue, with knee replacement being the least vascular of the 4 surgical models assessed and having the slowest rate of release.^37^ In knee replacement, 30% of the bupivacaine is released in the first 24 hours, compared with the more vascular hemorrhoidectomy, in which 90% is released.^37^ This will have an effect on the periarticular local anesthetic concentration, and it may be that a higher dose of liposomal bupivacaine is required in knee replacement, with the original phase 2 dose-finding trial in knee replacement finding a significant reduction in pain score AUC at rest from days 2 to 5 with 532 mg, but not with lower doses, which have subsequently been licensed.^17^

To our knowledge, this is the largest study of liposomal bupivacaine for the management of postoperative recovery and pain. It addresses many of the limitations of previous studies in that it has a patient-centered outcome measure and is appropriately powered with a standardized intervention with good adherence to the injection technique. Conducted across 11 centers within the National Health Service, this pragmatic randomized clinical trial reflects current clinical practice in the UK, with the results being directly applicable to decision-making by patients, clinicians, and policy makers.

### Limitations

This study is not without limitations. As a pragmatic randomized clinical trial, it reflects real-world experience and is not subject to the same standardization as other trial designs. A consequence is that variation in anesthetic and surgical practice as well as patient selection for knee replacement, which may influence outcomes, is expected between trial sites. Although stratification of randomization and adjustment for potential confounders during statistical analysis is designed to minimize the influence of this variation, the results of this trial represent the outcomes of the population studied as a whole. Within this population, there may be subgroups of patients in whom liposomal bupivacaine is associated with a positive (or negative) treatment effect but that the study is not powered to assess. Another limitation of this study is that outside of those recorded using the Clavien-Dindo classification, opioid-related adverse events were not specifically recorded; however, because no difference in opioid consumption was detected between groups, and, given that opioid-related adverse events are known to be dose dependent, a difference would not be expected. Last, because pain score and opioid consumption have been reported to be positively correlated, opioid consumption may represent a confounding factor for our primary outcome.

## Conclusions

In this randomized clinical trial, periarticular liposomal bupivacaine together with bupivacaine hydrochloride did not improve postoperative recovery or pain compared with bupivacaine hydrochloride alone in patients who had undergone knee replacement surgery. In addition, the intervention was not found to be cost-effective.

## Notes

Supplement 1.
Trial Protocol

Supplement 2. **eAppendix 1.** Participating Centers **eAppendix 2.** Data and Safety Monitoring Committee **eMethods.** Health Economic Analysis Plan **eTable 1.** Unit Costs (UK 2019 £) of Health Care Services **eTable 2.** Costs for Hospital Admissions Undergoing Knee Replacement **eTable 3.** Cost of Hospital Admissions During Follow-up (2019 Prices) **eTable 4.** Unit Costs for Equipment (2019 Prices) **eTable 5.** Unit Costs (UK 2019 £) of Time Away From Paid Employment **eTable 6.** Stratification Factors at Baseline–Split by Treatment Group and Overall **eTable 7.** Baseline Characteristics of Participants–Split by Treatment Group and Overall **eTable 8.** Primary and Secondary Outcome Measures at Baseline–Split by Treatment Group and Overall **eTable 9.** Details of Surgery and Complications Experienced During Surgery Summarized by Treatment Group **eTable 10.** Details of Withdrawals From Follow-up (and Reasons) Split by Treatment Group **eTable 11.** Details of Interventions Received Split by Randomized Treatment Group **eTable 12.** Analysis of Coprimary Outcomes at Primary End Points (ITT Population) **eTable 13.** Analysis of Coprimary Outcomes at Primary End Points (PP Population) **eTable 14.** Secondary Analysis of Primary Outcomes (ITT Population) **eTable 15.** Oral Morphine Equivalent Daily and Cumulative Opiate Use by Treatment Group **eTable 16.** Analysis of OKS and AKSS Secondary Outcome **eTable 17.** Analysis of Categorical Secondary Outcomes **eTable 18.** Clavien-Dindo Classification of Surgical and Inpatient Complications by Treatment Arm **eTable 19.** Summary of Serious Adverse Events (SAEs) Including SARs/SUSARs **eTable 20.** Sensitivity Analysis for Pain VAS Coprimary Outcome (ITT Population) **eTable 21.** Missing Data on Resource Use and EQ-5D Utility by Treatment Allocation in Each Follow-up Period **eTable 22.** EQ-5D-5D Utility Scores, EQ-5D-Visual Analog Scale Score (VAS), and Quality-Adjusted Life-Years (QALYs) by Treatment Allocation (Imputed Data) **eTable 23.** Details of the Index Procedure (Only Including Participants Who Received the Surgery) **eTable 24.** Summary of Cost (£) Component Over the 1-Year Follow-up (Imputed Data) **eTable 25.** Life-Years, Quality-Adjusted Life-Years, Health Care Costs, and Cost-effectiveness for the Base-Case Analysis at 1 Year Following Multiple Imputation **eTable 26.** Total NHS and PSS Costs (Including Intervention) in US Dollars **eTable 27.** Previous RCT Comparing 266-mg Liposomal Bupivacaine Against Periarticular Infiltration With Local Anesthetic **eFigure 1.** Forest Plot Showing the Treatment Effect of the Intervention vs Control Based on the QoR-40 Coprimary Outcome Between Recruitment Sites **eFigure 2.** rctmiss Sensitivity Analysis for QoR-40 Coprimary Outcome **eFigure 3.** Cost-effective Scatterplot for the Base Case Analysis (Imputed Data and Using NHS and PSS Perspective) **eFigure 4.** Cost-effectiveness Acceptability Curve for the NHS and Personal and Social Services Perspective and the Societal Perspective for the Base Case Analysis (Imputed Data)
eReferences

Supplement 3.
Nonauthor Collaborators

Supplement 4.
Data Sharing Statement
