# Supplementary material for: Efficacy of Liposomal Bupivacaine and Bupivacaine Hydrochloride vs Bupivacaine Hydrochloride Alone as a Periarticular Anesthetic for Patients Undergoing Knee Replacement: A Randomized Clinical Trial
Source: JAMA Surg. 2022 Apr 6;157(6):481–9. doi: 10.1001/jamasurg.2022.0713 (PMC8988023; doi:10.1001/jamasurg.2022.0713)
Supplement: Supplement 1. — Trial Protocol [file jamasurg-e220713-s001.pdf]

Trial Title: SPAARK: Study of Peri-Articular Anaesthetic for Replacement of the Knee.

The clinical and cost effectiveness of peri-articular liposomal bupivacaine plus bupivacaine hydrochloride compared with bupivacaine hydrochloride alone for post-operative recovery after knee replacement surgery: A multi-centre, patient-blinded, randomised controlled trial.

Short title: Liposomal bupivacaine in knee replacement surgery

**Protocol Version & date:**	03Feb2020 V6.0
**Sponsor Protocol Number:**	OR16/88494
**EudraCT Number:**	2016-003154-32
**IRAS ID:**	197936
**ISRCTN:**	ISRCTN54191675
**NIHR portfolio number:**	32227

**Chief Investigator:**	Professor Hemant Pandit, Professor of Orthopaedic Surgery<br>Nuffield Department of Orthopaedics, Rheumatology and Musculoskeletal Sciences (NDORMS) and Leeds Institute of Rheumatic and Musculoskeletal Medicine (LIRMM)<br>University of Oxford and University of Leeds<br>Hemant.Pandit@ndorms.ox.ac.uk; h.pandit@leeds.ac.uk
**Investigators:**	Mr Thomas Hamilton, NIHR Clinical Research Fellow<br>Nuffield Department of Orthopaedics, Rheumatology and Musculoskeletal Sciences<br>Oxford University Hospitals NHS Foundation Trust / University of Oxford<br>Thomas.Hamilton@ndorms.ox.ac.uk<br><br>Professor David Murray, Professor of Orthopaedic Surgery<br>Nuffield Department of Orthopaedics, Rheumatology and Musculoskeletal Sciences<br>Oxford University Hospitals NHS Foundation Trust / University of Oxford<br>David.Murray@ndorms.ox.ac.uk<br><br>Professor David Beard, Professor of Musculoskeletal Sciences / Director RCS

© Copyright: The University of Oxford and Oxford University Hospitals NHS Trust 2016

	Surgical Trials Unit, Oxford<br>Surgical Intervention Trials Unit (SITU)<br>University of Oxford<br>David.Beard@ndorms.ox.ac.uk<br><br>Professor Jonathan Cook, Associate Professor / Deputy-Director RCS Surgical Trials Unit, Oxford<br>Surgical Intervention Trials Unit (SITU)<br>University of Oxford<br>Jonathan.Cook@ndorms.ox.ac.uk<br><br>Mrs Susan Dutton, Senior Medical Statistician / Lead Statistician Oxford Clinical Trials Research Unit<br>Centre for Statistics in Medicine<br>University of Oxford<br>Susan.Dutton@csm.ox.ac.uk<br><br>Dr Jose Leal, Senior Health Economist<br>Health Economics Research Centre<br>University of Oxford<br>Jose.Leal@dph.ox.ac.uk<br><br>Professor Sallie Lamb, Professor of Rehabilitation / Director of Oxford Clinical Trials Research Unit<br>Oxford Clinical Trials Research Unit<br>University of Oxford<br>Sarah.Lamb@ndorms.ox.ac.uk<br><br>Dr Karen Barker, Consultant Physiotherapist<br>Director Musculoskeletal Services<br>Nuffield Orthopaedic Centre<br>Oxford<br>Karen.Barker@ouh.nhs.uk<br><br>Professor Mansukh Popat, Professor of Anaesthetics<br>Nuffield Department of Anaesthetics<br>University of Oxford<br>Mansukh.Popat@ndcn.ox.ac.uk
**Sponsor:**	UoL/LTHT Joint Sponsor QA office (CTIMPs)<br>Research & Innovation Centre

© Copyright: The University of Oxford and Oxford University Hospitals NHS Trust 2016

	Leeds Teaching Hospitals NHS Trust/University of Leeds<br>St James University Hospital<br>Leeds<br>LS9 7TF
**Funder:**	Research for Patient Benefit (RfPB)<br>National Institute for Health Research<br>Central Commissioning Facility<br>Grange House, 15 Church Street<br>Twickenham, TW1 3NL
**Chief Investigator Signature:**<br><br>**Statistician Signature:**<br><br>**Disclosures**	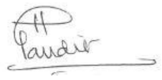<br>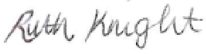<br>None of the applicants have any conflicts or potential conflicts of interest with the subject of this trial.

Confidentiality Statement

This document contains confidential information that must not be disclosed to anyone other than the Sponsor, the Investigator Team, host organisation, and members of the Research Ethics Committee, unless authorised to do so.

© Copyright: The University of Oxford and Oxford University Hospitals NHS Trust 2016

TABLE OF CONTENTS

1. KEY TRIAL CONTACTS 6
2. SYNOPSIS 8
3. ABBREVIATIONS 10
4. BACKGROUND AND RATIONALE 12
5. OBJECTIVES AND OUTCOME MEASURES 14
6. TRIAL DESIGN 15
6.1. Summary of Trial Design 15
6.1.1. Bupivacaine Hydrochloride versus Liposomal Bupivacaine Hydrochloride 15
6.2. Number of Centres 16
6.3. Patient Participation 16
7. PARTICIPANT IDENTIFICATION 17
7.1. Trial Participants 17
7.2. Inclusion Criteria 17
7.3. Exclusion Criteria 17
8. TRIAL PROCEDURES 18
8.1. Patient Identification and Recruitment 18
8.2. Informed Consent 18
8.3. Randomisation, Blinding and Code-breaking 19
8.4. Baseline Assessments 20
8.5. Subsequent Assessments & Visits 20
8.6. Discontinuation/Withdrawal of Participants from Trial 21
8.6.1. Withdrawal from Follow Up 21
8.6.2. Withdrawal of Consent 21
8.6.3. Change of Treatment 21
8.7. Definition of End of Trial 21
9. INVESTIGATIONAL MEDICINAL PRODUCT (IMP) 21
9.1. IMP Description 21
9.1.1. Name of IMP: 21
9.1.2. Control: 22
9.2. IMP Administration 22
9.2.1. Prior to implantation of definitive implants 22
9.2.2. After implantation of definitive implants 22
9.3. Storage of IMP 23
9.4. Compliance with Trial Treatment 23

© Copyright: The University of Oxford and Oxford University Hospitals NHS Trust 2016

9.5. Accountability of the Trial Treatment 23
9.6. Concomitant Medication 23
9.6.1. Post-Operative Analgesia 23
9.7. Post-trial Treatment 24
10. SAFETY REPORTING 24
10.1. Definitions 24
10.2. Causality 25
10.2.2 Events Related to Knee Replacement Surgery 26
10.3. Procedures for Recording Adverse Events & Reporting Serious Adverse Events 26
10.4. Assessment of Expectedness 27
10.5. SUSAR Reporting 27
10.6. Data Safety and Monitoring Committee & Trial Steering Committee 27
10.7. Development Safety Update Reports 28
11. STATISTICS 28
11.1. Description of Statistical Methods 28
11.2. The Number of Participants 29
11.3. The Level of Statistical Significance 30
11.4. Procedure for Accounting for Missing, Unused, and Spurious Data 30
11.5. Procedures for Reporting any Deviation(s) from the Original Statistical Plan 30
12. DATA MANAGEMENT AND SHARING PLAN 30
13. QUALITY ASSURANCE PROCEDURES 31
14. SERIOUS BREACHES 31
15. ETHICAL AND REGULATORY CONSIDERATIONS 31
15.1. Declaration of Helsinki 31
15.2. Guidelines for Good Clinical Practice 31
15.3. Approvals 31
15.4. Reporting 32
15.5. Participant Confidentiality 32
15.6. Expenses and Benefits 32
16. FINANCE AND INSURANCE 32
16.1. Funding 32
16.2. Insurance 32
17. PUBLICATION POLICY 32
18. APPENDIX A: TRIAL FLOW CHART 34
19. APPENDIX B: ASSESSMENT TABLE 35

© Copyright: The University of Oxford and Oxford University Hospitals NHS Trust 2016

20. APPENDIX C: AMENDMENT HISTORY 36
21. REFERENCES 37

1. KEY TRIAL CONTACTS

**Chief Investigator**	Hemant Pandit, Professor of Orthopaedic Surgery<br><br>Botnar Research Centre<br>Nuffield Department of Orthopaedics, Rheumatology & Musculoskeletal Sciences, University of Oxford<br>Windmill Road<br>Oxford OX3 7LD<br>Tel: 01865 227457<br>Fax: 01865 227671<br>Email: hemant.pandit@ndorms.ox.ac.uk<br>h.pandit@leeds.ac.uk
**Sponsor**	UoL/LTHT Joint Sponsor QA office (CTIMPs)<br>Research & Innovation Centre<br>Leeds Teaching Hospitals NHS Trust/University of Leeds<br>St James University Hospital<br>Leeds<br>LS9 7TF
**Clinical Trials Unit**	Oxford Clinical Trials Research Unit<br>Botnar Research Centre<br>Nuffield Orthopaedic Centre<br>Windmill Road<br>Oxford OX3 7LD<br>Tel: 01865 223463<br>Email: octrutrialshub@ndorms.ox.ac.uk
**Trial Manager**	Lisa Poulton<br><br>Surgical Intervention Trials Unit<br>Nuffield Department of Orthopaedics, Rheumatology & Musculoskeletal Sciences<br>University of Oxford<br>Oxford OX3 7LD<br>Tel: 01865 223665<br>Email: spaark@ndorms.ox.ac.uk
**Statistician**	Ruth Knight, Medical Statistician<br><br>Oxford Clinical Trials Research Unit<br>Centre for Statistics in Medicine<br>Nuffield Department of Orthopaedics, Rheumatology & Musculoskeletal Sciences

© Copyright: The University of Oxford and Oxford University Hospitals NHS Trust 2016

	University of Oxford<br>Oxford OX3 7LD<br>Tel: 01865 227982<br>Email: ruth.knight@csm.ox.ac.uk
**Committees**	Data Safety and Monitoring Committee:<br><br>Dr Vassilis Athanassoglou (Chair, Independent)<br>Dr Rajesh Rout (Independent)<br>Miss Katie Pike (Independent)<br><br>Trial Steering Committee:<br><br>Mr Stephen McDonnell (Chair, Independent)<br>Professor Hemant Pandit (Non-independent)<br>Dr Milica Bucknall (Independent)<br>Mrs Rosalind Clow (Independent)<br>Mrs Rosemary Wyber (Independent)<br>Trial Management Delegate(s) (Non-independent)<br>Mr Simon Newman (Independent)<br>Dr Sabeena Sharma (Independent)

© Copyright: The University of Oxford and Oxford University Hospitals NHS Trust 2016

2. SYNOPSIS

Trial Title	SPAARK: Study of Peri-Articular Anaesthetic for Replacement of the Knee.<br>The clinical and cost effectiveness of periarticular liposomal bupivacaine plus bupivacaine hydrochloride compared with bupivacaine hydrochloride alone for post-operative recovery after knee replacement surgery: A multi-centre, patient-blinded, randomised controlled trial.
Internal ref. no. (or short title)	Liposomal bupivacaine in knee replacement surgery
Clinical Phase	III
Trial Design	Randomised controlled trial
Trial Participants	Adult patients undergoing knee replacement surgery
Planned Sample Size	500
Treatment duration	Single dose of liposomal bupivacaine with bupivacaine hydrochloride or bupivacaine hydrochloride control given intra-operatively
Follow up duration	12 months
Planned Trial Period	1 March 2017 to 14Aug2021
Inclusion Criteria	• Unilateral primary knee replacement, including both total knee replacement (TKR) or unicompartmental knee replacement (UKR), for end stage osteoarthritis<br>• American Society of Anaesthesiologists (ASA) Grade I to III<br>• Participant is willing and able consent for themselves<br>• Male or Female, aged 18 years or above.<br>• In the Investigator's opinion, is able and willing to comply with all trial requirements.
Exclusion Criteria	• Allergy or intolerance to amide type local anaesthetics<br>• Objective evidence of nerve damage in the affected lower limb.<br>• Rheumatoid arthritis<br>• Any other significant disease, disorder or condition which, in the opinion of the Investigator, may either put the participants at risk because of participation in the trial, or may influence the results of the trial, or the participant's ability to participate in the trial.<br>• Participants who have participated in another research trial involving an investigational product in the past 6 months.<br>• Participants who have significant cognitive impairment or language issues<br>• Contra-lateral knee replacement within the trial or within 12 months prior to randomisation

© Copyright: The University of Oxford and Oxford University Hospitals NHS Trust 2016

	Objectives	Outcome Measures/Endpoints
Primary	Evaluate the effectiveness of liposomal bupivacaine with bupivacaine hydrochloride compared to bupivacaine hydrochloride alone on post-operative recovery. Post-operative recovery will be assessed both in terms of systemic recovery as well as local recovery of the operated joint.	Quality of Recovery 40 Score at 72 hours.<br><br>Cumulative daily pain score at rest using 0-10 VAS from 0 to 72 hours following surgery.
Secondary	To assess other markers of recovery both in the short term and long term.<br><br>To evaluate the cost effectiveness of the intervention compared with the current standard of care.<br><br>Safety	Mean pain score measured using a 0-10 VAS at: baseline, evening of surgery (day 0) and days 1, 2 and 3 following surgery.<br><br>Quality of Recovery 40 Score at baseline, evening of surgery (day 0) and days 1, 2 and 3 following surgery.<br><br>Cumulative opioid consumption over 72 hours.<br><br>Fitness for discharge against pre-defined criteria at: evening of surgery (day 0) and days 1, 2 and 3 following surgery.<br><br>Functional outcome at baseline, 6 weeks, 6 months and 1 year (Oxford Knee Score, American Knee Society Score)<br><br>Cost utility analysis using patient-reported quality of life as the main outcome, obtained using the EuroQol EQ-5D-5L questionnaire at baseline, 72 hours, 6 weeks, 6 months and 1 year.<br><br>Serious adverse events up to 12 months post-randomisation.<br><br>Related AEs up to 30 days post-surgery.<br><br>Intraoperative and inpatient complications, specifically cardiovascular or wound complications.
Investigational Medicinal Product	<u>Intervention:</u> Liposomal bupivacaine (EXPAREL, Pacira Pharmaceuticals Inc., San Diego, California, USA). Liposomal bupivacaine is a local anaesthetic injected to provide pain relief. Liposome encapsulated bupivacaine has been reported to have a longer activing effect than normal bupivacaine. The active component is held within multi-vesicular liposomes which permit prolonged release through diffusion, membrane breakdown and re-organisation. It is not yet licensed in the UK.	

© Copyright: The University of Oxford and Oxford University Hospitals NHS Trust 2016

	Control: Bupivacaine hydrochloride. This is currently the product of choice in the NHS for intra-articular injection and is widely available as a local anaesthetic in healthcare. It is not encapsulated in liposomes and its action is reportedly quicker than the intervention.
Formulation, Dose, Route of Administration	266mg injectable suspension for periarticular infiltration

3. ABBREVIATIONS

AE	Adverse Event
AR	Adverse Reaction
ASA	American Society of Anaesthesiologists
CI	Chief Investigator
CRA	Clinical Research Associate (Monitor)
CRF	Case Report Form
CT	Clinical Trials
CTA	Clinical Trials Authorisation
DSMC	Data Safety and Monitoring Committee
DSUR	Development Safety Update Report
GA	General Anaesthetic
GCP	Good Clinical Practice
GP	General Practitioner
HRA	Health Research Authority
IB	Investigators Brochure
ICF	Informed Consent Form
ICH	International Conference on Harmonisation
IMP	Investigational Medicinal Product
IRB	Independent Review Board
LA	Local Anaesthetic
MHRA	Medicines and Healthcare products Regulatory Agency
NHS	National Health Service
NIHR	National Institute for Health Research
NRES	National Research Ethics Service
NSAID	Non-steroidal Anti-inflammatory Drug
PI	Principal Investigator

© Copyright: The University of Oxford and Oxford University Hospitals NHS Trust 2016

PIL	Participant/ Patient Information Leaflet
QA	Quality Assurance
R&D	NHS Trust R&D Department
REC	Research Ethics Committee
RfPB	Research for Patient Benefit
RRAMP	Registration/Randomisation and Management of Product
SAE	Serious Adverse Event
SAR	Serious Adverse Reaction
SDV	Source Data Verification
SMPC	Summary of Medicinal Product Characteristics
SOP	Standard Operating Procedure
SUSAR	Suspected Unexpected Serious Adverse Reactions
TKR	Total Knee Replacement
TMF	Trial Master File
TSC	Trial Steering Committee
UKR	Unicompartmental Knee Replacement
VAS	Visual Analogue Scale

© Copyright: The University of Oxford and Oxford University Hospitals NHS Trust 2016

4. BACKGROUND AND RATIONALE

The treatment of acute post-operative pain remains an unmet health need. Despite the development of guidelines [1-3] to assist clinicians and allied health professionals to recognize and treat the so called 'fifth vital sign' it has been reported that up to three quarters of surgical patients receive inadequate pain relief with 40% of patients reporting severe pain following knee replacement [4-7].

Around 100,000 primary knee replacements were performed in the UK last year and optimizing the management of peri-operative pain and recovery has been identified as a patient priority [8]. Optimizing peri-operative pain management through the use of multi-modal analgesia reduces the surgical stress response and permits early rehabilitation. Optimizing recovery has many benefits both to the patient in terms of reduced morbidity and mortality and the healthcare system through reduced healthcare associated costs [9]. Current pain relief strategies use opiate base analgesia, however these drugs have significant side effects, which are seen in up to 50% of patients, and include nausea, vomiting, pruritus, drowsiness and hypotension with respiratory depression seen in severe cases. Using opiate sparing techniques minimizes these side effects, permitting patients to engage with early rehabilitation. Currently, managing post-operative pain as well as the side effects of morphine analgesia, which both delay early mobilization, account for a significant proportion of the average 3 to 6 day hospital stay. Opioid sparing techniques have been associated with enhanced patient satisfaction in the acute phase and there is increasing evidence about their longer term benefits with studies reporting a lowered incidence of chronic postsurgical pain, though the full effect on long term patient reported outcome measures is unknown [10-12].

The concept of multi-modal analgesia was introduced over twenty years ago and its use expanded to many areas of surgery [13]. Multi-modal analgesia employs a range of techniques to inhibit the multiple pathways of nociceptive stimuli along their path from the site of surgical injury, passing through the peripheral nervous system to the central nervous system. Using non-steroidal anti-inflammatory drugs (NSAIDs), cyclooxygenase-2 inhibitors, paracetamol, gabapentinoids, ketamine, and local and regional anaesthetic techniques reduce the need for oral or parenteral opioids in the post-operative period, and as a consequence their side effects, is reduced. Multiple studies report the superiority of multi-modal analgesia over single agent therapy [2].

Local anaesthetic incisional infiltration is commonly used as part of a multi-modal technique with the view that modification of pain stimuli at their origin will reduce the transmission of nociceptive stimuli, thereby reducing downstream organ dysfunction, pain and stress responses, including centrally mediated changes in the spinal cord or the cerebral cortex which all contribute to post-operative morbidity and mortality. The half-life of local anaesthetics is dependent on the rate at which they disassociate from voltage-gated sodium channels with bupivacaine being one of the longest acting local anaesthetics with a half-life of 120 to 240 minutes [14]. Whilst the clearance and subsequent hepatic metabolism of bupivacaine can be delayed by the addition of drugs such as epinephrine the length of their duration of action is a major limiting factor of current local anaesthetic techniques and despite multi-modal techniques a significant number of patients report severe post-operative pain following knee replacement. Current methods to address this, including continuous short acting local anaesthetic infusions, remain unsatisfactory due to the increased risk of infection, interference with early mobilization and discharge caused by the use of an indwelling infiltration catheter as well as additional cost of infusion devices. Due to the inherent benefits of targeting peripheral receptors a great deal of interest has been invested in developing long acting local anaesthetics such as liposomal bupivacaine.

© Copyright: The University of Oxford and Oxford University Hospitals NHS Trust 2016

Liposomal bupivacaine (EXPAREL, Pacira Pharmaceuticals Inc., San Diego, California 92121) is liposome encapsulated bupivacaine which has been reported to be effective for up to 72 hours [15, 16]. Using a proprietary technique known as DepoFoam (SkyePharma, London, UK) the local anaesthetic bupivacaine is held within multi-vesicular liposomes which permit prolonged release through diffusion, membrane breakdown and re-organisation [17].

Liposomal Bupivacaine was licensed by the Food and Drug Administration in October 2011 and large centres are able to discharge patients on the day of surgery following hip and knee replacement surgery with no reported incidents of increased readmission rates or wound issues [18-20]. Currently liposomal bupivacaine is not yet licensed in the UK for any indication and its use has been limited to phase II clinical trials. However the active component, bupivacaine hydrochloride, is licensed and widely used as local anaesthetic. Furthermore, liposomal formulations, utilising an identical liposome structure but different active ingredient, are licensed for use in infectious diseases and oncology and liposomal morphine is licensed for pain relief [21].

In small clinical trials conducted by Pacira Pharmaceuticals, liposomal bupivacaine administered as a single intra-operative dose by wound infiltration, is well tolerated and has been reported to be associated with significantly lower pain scores, opiate usage, opiate related adverse effects as well as a reduced length of stay. Liposomal bupivacaine (300 USD/patient) has been reported to be cost effective in gastrointestinal surgery in the US and a recent open label prospective case-controlled study comparing 1000 total joint replacements using liposomal bupivacaine based multimodal therapy to 1000 total joint replacements using standard pain management regimens found a savings of $420 per case associated with the use of liposomal bupivacaine [20].

A series of randomised controlled trials have investigated the use of Liposomal Bupivacaine administered either as a peripheral nerve block or via infiltration at the surgical site for post-operative pain. The results of these studies have been assessed as part of two Cochrane Reviews performed by ourselves. We concluded that there was not sufficient evidence to support the use of liposomal bupivacaine administered as a peripheral nerve block in the management of post-operative pain following knee replacement [22]. Liposomal bupivacaine at the surgical site does appear to reduce postoperative pain compared to placebo, however, at present the limited evidence does not demonstrate superiority to bupivacaine hydrochloride [23]. Given the potential impact of liposomal bupivacaine we have decided to conduct a large, high quality RCT, to establish whether liposomal bupivacaine has a role in the management of post-operative pain within the NHS.

This study is designed to assess the clinical and cost effectiveness of liposomal bupivacaine in patients undergoing knee replacement surgery. The rationale for assessing the efficacy in this population is that knee replacement is known to be a painful procedure and that poor post-operative pain management is associated with delayed mobility, knee stiffness, increased risk of Deep Vein Thrombosis (DVT) or Pulmonary Embolism (PE), increased length of stay, decreased satisfaction and poor long term functional outcome. Liposomal bupivacaine may allow early mobilisation and discharge bringing significant benefits to patients at a reduced costs to healthcare providers. Previous studies carried out in the USA are on heterogeneous groups of patients and the number of patients per intervention are small. The data from the USA on efficacy of liposomal bupivacaine is equivocal and past studies have been industry-led. The patient population, local practices, as well as perioperative managements differ in the USA and in the UK and it is important to establish whether this drug will be beneficial in the NHS to reduce length of hospital stay, improve pain scores and clinical outcomes.

© Copyright: The University of Oxford and Oxford University Hospitals NHS Trust 2016

In a multi-centre, patient-blinded, randomised controlled trial we will assess clinical and cost effectiveness of peri-articular liposomal bupivacaine and bupivacaine hydrochloride compared with peri-articular bupivacaine hydrochloride alone in patients undergoing knee replacement surgery within the NHS. The results of this study will be directly applicable to the 100,000 patients who undergo knee replacement surgery in the UK each year and the results will help to inform healthcare providers as well as healthcare payers about the potential role that liposomal bupivacaine has within the NHS. In addition the results of this study will provide key results and play a pivotal role in informing further surgical trials across a range of surgical specialties and surgical sites in the UK.

In summary:

- A Cochrane review has concluded that there is not sufficient evidence to support the use of Liposomal Bupivacaine in the management of post-operative pain. Also, further Randomised controlled trials are required due to the low quality and quantity of these studies.
- Anecdotal evidence from US and a large open label case-control study suggest clinical and cost effectiveness, with same day discharge following joint replacement being performed at several centres.
- There is a lack of evidence from appropriately designed trials of the clinical and cost effectiveness of liposomal bupivacaine in knee replacement surgery.
- This simple intervention has the potential to be both clinically and cost effective but requires further evaluation to establish its role within the setting of the NHS.

5. OBJECTIVES AND OUTCOME MEASURES

	Objectives	Outcome Measures/Endpoints
Primary	Evaluate the effectiveness of liposomal bupivacaine with bupivacaine hydrochloride compared to bupivacaine hydrochloride alone on post-operative recovery. Post-operative recovery will be assessed both in terms of systemic recovery as well as local recovery of the operated joint.	Quality of Recovery 40 Score at 72 hours. The QoR-40 is a six dimension validated patient reported peri-operative recovery score that has been widely used across a range of surgeries including knee replacement and assesses overall post-operative recovery[24].<br><br>Cumulative daily pain score at rest using a 0-10 VAS from 0 to 72 hours following surgery.
Secondary	To assess other markers of recovery both in the short term and long term.	Mean pain score measured using a 0-10 VAS at: baseline, evening of surgery (day 0) and days 1, 2 and 3 following surgery.<br><br>Quality of Recovery 40 Score at baseline, evening of surgery (day 0) and days 1, 2 and 3 following surgery.<br><br>Cumulative opioid consumption over 72 hours.

© Copyright: The University of Oxford and Oxford University Hospitals NHS Trust 2016

		Fitness for discharge against pre-defined criteria at: evening of surgery (day 0) and days 1, 2 and 3 following surgery.<br><br>Functional outcome using validated, patient reported (Oxford Knee Score[25]) and qualitative (American Knee Society Score[26]) outcome scores at baseline, 6 weeks, 6 months and 1 year following surgery.
	To evaluate the cost effectiveness compared with the current standard of care.	Cost utility analysis using patient-reported quality of life as the main outcome, obtained using the EuroQol EQ5D-5 Level questionnaire at baseline, 72 hours, 6 weeks, 6 months and 1 year.
	Safety	Serious adverse events up to 12 months post-randomisation.<br><br>Related AEs up to 30 days post-surgery.<br><br>Intraoperative and in-patient complications, specifically cardiovascular or wound complications. . These will be recorded using the Clavien Dindo Classification [27].

6. TRIAL DESIGN

6.1. Summary of Trial Design

The trial design is a patient-blinded multi-centre, active comparator, randomised controlled two-arm parallel group superiority trial (RCT) of liposomal bupivacaine plus bupivacaine hydrochloride versus bupivacaine hydrochloride alone for post-operative pain in patients undergoing knee replacement surgery. Patients will be randomised in a 1:1 ratio. The RCT design is robust and reduces any potential bias. Patients will be blinded as to which treatment they receive. Blinding of patients is possible as they will be under general anaesthetic for their knee replacement surgery at the time of the drug administration. Blinding of surgeons, who administer the medication and outcome assessors is not necessary as the primary outcome is a patient reported outcome measure.

6.1.1. Bupivacaine Hydrochloride versus Liposomal Bupivacaine Hydrochloride

Previous studies have demonstrated a delay between wound infiltration with liposomal bupivacaine and the active component, bupivacaine hydrochloride, being detected in peripheral blood. This delay appears to be associated with a delayed onset of analgesia with liposomal bupivacaine and has been proposed to be due to the time required for the active component to be released from liposomes and diffuse to the site of action. Since December 2015 it has

© Copyright: The University of Oxford and Oxford University Hospitals NHS Trust 2016

been recommended that liposomal bupivacaine is admixed with bupivacaine and co-administered in the same syringe to enhance early postoperative analgesia [28].

Amide type local anesthetics, including bupivacaine hydrochloride, have long been used for peri-articular infiltration. Historically bupivacaine hydrochloride was the drug of choice, and whilst this still remains the standard treatment in many centres, there is an increasing trend towards using ropivacaine hydrochloride. The reason for using ropivacaine hydrochloride is based on the lower cardiac and neurological toxicity seen in ropivacaine, in comparison to bupivacaine, and not due to differences in efficacy [29, 30].

We have chosen to use bupivacaine hydrochloride for this study as this represents the active component of liposomal bupivacaine, making the comparison more applicable, with the doses stated in this study well below established safety thresholds.

Bupivacaine hydrochloride, when injected immediately before or admixed with liposomal bupivacaine, may impact the pharmacokinetic and/or physicochemical properties of the drugs if the milligram dose of bupivacaine hydrochloride solution exceeds 50% of the liposomal bupivacaine dose. The doses proposed in this trial (100 mg bupivacaine hydrochloride added to 266 mg liposomal bupivacaine) falls well below this threshold so no significant interaction is expected.

6.2. Number of Centres

Participants will be recruited from approximately 10 NHS hospitals throughout the United Kingdom. The number of recruiting centres may be increased as the study progresses. Any additional sites will be added via a notification of amendment form to the REC and HRA, following approval by the Sponsor.

6.3. Patient Participation

Patients will be involved for a maximum of 12 months post-randomisation. Post operatively, patients will be asked to complete Quality of Recovery-40 assessment daily and a visual analogue scale (VAS) pain score 0-10 on the evening of surgery (day 0) and on days 1, 2 and 3 following surgery. The analgesia requirements of each patient will be recorded. The time of surgery for the patient will be noted. The day of surgery will be called Day 0.

If the patients are discharged home prior to 72 hours postoperatively, then a phone call will be made daily to the patient to collect pain VAS and QoR-40. Pain VAS will be collected by asking the question 'How painful has your operated knee been over the past 24 hours on a scale of 0-10?' The EQ-5D-5L section of the questionnaire will also be completed over the telephone if deemed appropriate. If the patient cannot be contacted between those hours, then an additional call will be made between 2pm and 6pm and this will be noted.

Fitness for discharge will be assessed as per routine clinical care on the evening of surgery (day 0) day 1, 2 and 3 following surgery, until the patient is deemed fit for discharge. This is assessed against the following criteria:

- Ability to mobilize independently
- Pain score less than or equal to 3cm on a 10cm VAS

© Copyright: The University of Oxford and Oxford University Hospitals NHS Trust 2016

- Ability to straight leg raise
- Ability to bend knee to 90 degrees

Once discharged, patients will be assessed as per routine clinical care. The Central Study Office will post questionnaires to patients for completion at 6 weeks post-surgery, and at 6 months and 1 year following randomisation. The local hospital research team should encourage timely completion of questionnaires if these time points coincide with the routine follow up appointments. Assessments will include completing the Quality of Recovery 40, Oxford Knee Score, American Knee Society Score and EQ-5D-5L. In addition, an analgesic history will be taken.

A trial flowchart is provided in appendix A.

7. PARTICIPANT IDENTIFICATION

7.1. Trial Participants

The Investigator will determine trial participant eligibility based on the criteria given below.

7.2. Inclusion Criteria

The participant may enter the trial if ALL of the following apply:

- Unilateral primary knee replacement, including both total knee replacement (TKR) or unicompartmental knee replacement (UKR), for end stage osteoarthritis
- American Society of Anaesthesiologists (ASA) Grade I to III
- Participant is willing and able consent for themselves
- Male or Female, aged 18 years or above.
- In the Investigator’s opinion, is able and willing to comply with all trial requirements

7.3. Exclusion Criteria

The participant may not enter the trial if ANY of the following apply:

- Allergy or intolerance to amide type local anaesthetics
- Objective evidence of nerve damage in the affected lower limb.
- Rheumatoid arthritis
- Any other significant disease, disorder or condition which, in the opinion of the Investigator, may either put the participants at risk because of participation in the trial, or may influence the results of the trial, or the participant’s ability to participate in the trial.
- Participants who have participated in another research trial involving an investigational product in the past 6 months.
- Participants who have significant cognitive impairment or language issues
- Contra-lateral knee replacement within the trial or within 12 months prior to randomisation

© Copyright: The University of Oxford and Oxford University Hospitals NHS Trust 2016

If the patient is receiving staged bilateral knee replacement, they may still participate in the trial but data will only be included from the first knee.

8. TRIAL PROCEDURES

8.1. Patient Identification and Recruitment

All patients listed for knee replacement, i.e. TKR and UKR, for end-stage osteoarthritis will be eligible. We expect to recruit a maximum of 10% of UKRs as this would reflect current practice in the UK.

Patients will be identified via two possible methods:

1. Patients will be identified at outpatient clinics by participating surgeons and their clinical care team. Potential patients will then be seen by the local research nurses/physiotherapist to discuss the study further and the PI or designee will confirm eligibility.

2. Potential patients may also be identified by the clinical team from surgical waiting lists. These patients will be sent an invitation letter and asked to contact the local team if they are willing to discuss the study further.

Potentially eligible patients will be given an invitation letter and information sheet explaining why they have been approached. The research team will discuss the study details with the patient. If patients are willing to proceed into the study, the research nurse or clinician will ask the patient whether they agree to consent at that time or need up to a week to discuss with family or friends and agree on an arrangement to confirm their decision.

If a further appointment is necessary for the patient to return to give consent and complete the baseline assessment, all efforts will be made to ensure this appointment is as convenient for the patient as possible and may coincide with their pre-operative assessment appointment. The pre-operative assessment appointments are routinely scheduled for a short time before the patient's scheduled operation date.

Data related to the number of potential patients identified and approached will be recorded on screening logs. The screening log will also depict the number of patients who, once assessed, are deemed ineligible and also those who decline to participate. Reasons for eligible patients not participating will be requested, but do not have to be given. Screening logs will not contain patient identifiable information.

8.2. Informed Consent

Informed consent will be taken by a medically-qualified and suitably experienced investigator. All delegates must be authorised by the Chief/Principal Investigator to obtain consent. The Investigator is responsible for ensuring that the trial consent procedures comply with current applicable GCP Regulatory and ethical requirements. The Investigator must be satisfied that the patient has made an informed decision before taking consent.

© Copyright: The University of Oxford and Oxford University Hospitals NHS Trust 2016

The participant must personally sign and date the latest approved version of the Informed Consent Form before any trial specific procedures are performed. Consent will be verbally re-confirmed on the day of surgery.

Written and verbal versions of the Participant Information and Informed Consent will be presented to the participants detailing no less than: the exact nature of the trial; what it will involve for the participant; the implications and constraints of the protocol; the known side effects and any risks involved in taking part. It will be clearly stated that the participant is free to withdraw from the trial at any time for any reason without prejudice to future care, and with no obligation to give the reason for withdrawal.

The participant will be allowed as much time as wished to consider the information, and the opportunity to question the Investigator, their GP or other independent parties to decide whether they will participate in the trial. The patient and the Investigator must personally sign and date the current approved version of the informed consent form in each other's presence. A copy of the signed Informed Consent will be given to the participant. The original signed form will be retained at the trial site.

8.3. Randomisation, Blinding and Code-breaking

Randomisation will be performed locally by the trial site using a secure online internet based system (RRAMP) provided by the Oxford Clinical Trials Research Unit (OCTRU).

Patients will be randomised 1:1 to the two treatment groups using stratified randomisation with variable block sizes, stratified by trial hospital and surgical procedure (TKR or UKR).

Due to local patient pathways, informed consent and baseline assessments may be completed at the same hospital visit however, it may be the case randomisation is not performed on the same day. Where possible, randomisation will occur on the day of surgery either by a member of the clinical research team or a member of the surgical team, where this is not possible randomisation may occur on the days prior to surgeryto allow for time to coordinate the drug supplies and inform the operative and anaesthetic team. If consent has occurred more than a day before randomisation, the local research team will verbally check the patient still agrees to participation before proceeding with randomisation.

Only trial team members listed on the delegation log are authorised to perform randomisations.

Patients will be blinded to the treatment allocation. Trial sites should make every effort to ensure that patient blinding is maintained throughout the duration of the trial so that no bias is introduced. The arrangements for maintaining patient blinding for the trial should be considered in detail and those arrangements carefully planned, documented and communicated to ensure accidental unblinding is avoided.

Methods for ensuring patient blinding should be discussed during the Site Initiation Visits, and may include only recording the drug given (active/control) in the surgeon notes as "as per protocol of SPAARK trial" but the actual drug given recorded in the anaesthetic chart. Local practice may vary and the trial sites can discuss with the central study team alternative ways to ensure that patient blinding is maintained.

Unblinding will be performed on an individual basis as clinical need dictates.

© Copyright: The University of Oxford and Oxford University Hospitals NHS Trust 2016

8.4. Baseline Assessments

Prior to randomisation the following information will be collected:

- Patient demographics (gender, age at surgery, body mass index)
- Summary medical history
- Summary medication history (current medication and duration of current medication, changes to current medication in the last 6 months, any history of prior opiate use)
- Quality of Recovery 40
- Pain Visual Analogue Scale (0-10) at rest
- Oxford Knee Score
- Clinical Assessment - American Knee Society Score
- Patient expectations of treatment
- Summary healthcare and social services use as well as informal care in the past 6 months due to knee (GP visits, outpatient visits, allied healthcare contacts, hospital admissions, medications, meals on wheels, and unpaid care)
- Summary employment history (in paid employment, not in employment, time away from work or usual activities)

  EuroQol EQ-5D-5L

8.5. Subsequent Assessments & Visits

Following surgery (day 0) and the 3 days following (days 1, 2 and 3) in the evening, the following information will be collected:

- Quality of Recovery 40
- Pain Visual Analogue Scale (0-10) at rest
- Cumulative opiate use (refer to patient medical notes)
- EuroQol EQ-5D-5L

Routine clinical assessments will also occur at the in-patient stage, including assessment of fitness to discharge. This information will be recorded for trial purposes.

Once discharged, patients will be seen again as per local routine clinical care. The Central Study Office will post questionnaires to patients for completion at 6 weeks post-surgery, and at 6 months and 1 year following randomisation. The following information will be collected:

- Summary medication history (since last assessment)
- Quality of Recovery 40
- Pain Visual Analogue Scale (0-10) at rest
- Oxford Knee Score
- Clinical Assessment - American Knee Society Score
- Summary healthcare and social services use as well informal care in past period due to knee (GP visits, outpatient visits, allied healthcare contacts, hospital admissions, medications, meals on wheels, and unpaid care)
- Summary employment history (in paid employment, not in employment, time away from work or usual activities)
- EuroQol EQ-5D-5L

© Copyright: The University of Oxford and Oxford University Hospitals NHS Trust 2016

If the patient does not return the postal questionnaire within 7 days, another questionnaire will be posted as a reminder. Following this, if we have not received a completed questionnaire within 14 days, all patient reported data will be recorded over the telephone.

An assessment table is provided in Appendix B.

8.6. Discontinuation/Withdrawal of Participants from Trial

Each participant has the right to withdraw from any aspect of the trial at any time.

8.6.1. Withdrawal from Follow Up

Participants may withdraw from the follow-up regime. If so, data up to the point of withdrawal will be collated and analysed accordingly.

8.6.2. Withdrawal of Consent

Randomisation will be undertaken as close to the surgery as possible. Therefore, it is unlikely patients will withdraw consent prior to surgery. If they do, this will be recorded and they can discuss treatment options with their surgeon.

8.6.3. Change of Treatment

For many reasons patients operations are cancelled, or patients withdraw themselves from the surgical waiting list. If patients have been randomised into the study and then do not go on to have surgery; reasons for cancellation/withdrawal will be recorded, if available and patients, if willing, will be followed up accordingly.

In the event of discontinuation or withdrawal from the trial the reason will be recorded on the Case Report Form (CRF). Analysis will be performed on an intention-to-treat basis.

8.7. Definition of End of Trial

The end of trial is 12 months after the last participant was randomised and once all data has been collected and all queries resolved.

9. INVESTIGATIONAL MEDICINAL PRODUCT (IMP)

9.1. IMP Description

Both the trial drug and active control are injectable solutions which are manufactured commercially. They will be supplied to theatre packaged in their original packaging. Following randomisation, the anaesthetist will prepare the drug in a standardised fashion and offer the drug to the scrub nurse to draw up into a syringe ready for administration.

9.1.1. Name of IMP:

Liposomal bupivacaine (EXPAREL, Pacira Pharmaceuticals Inc., San Diego, California)

© Copyright: The University of Oxford and Oxford University Hospitals NHS Trust 2016

266mg Liposomal bupivacaine will be mixed with 100mg bupivacaine hydrochloride. The volume will then be expanded to 120ml with normal saline and administered as a single dose intra-operatively by periarticular infiltration.

The IMP will be supplied to sites free of charge by Pacira Pharmaceuticals Inc. Initial supplies will be ordered for each site by the Central Study Team following site initiation and confirmation from the Sponsor that all approvals are in place and the site is open to recruitment. For re-ordering of supplies, contact the Central study Team who will arrange further supplies.

9.1.2. Control:

Bupivacaine hydrochloride (must be plain, with no adrenaline/epinephrine present)

100mg bupivacaine hydrochloride alone. The volume will then be expanded to 120ml with normal saline and administered as a single dose intra-operatively by periarticular infiltration.

The bupivacaine used in the control arm will not be standardised by brand. The brand used will be what is available to the local participating trusts.

As per standard practice, 100mg Bupivacaine hydrochloride is a flat dose and will not be altered according to BMI in either the intervention or treatment arm.

9.2. IMP Administration

The study drug will be administered using six 20 cc syringes with 21 or 22-gauge needles prior to wound closure.

Surgeons will be trained in the injection technique which will be as follows:

9.2.1. Prior to implantation of definitive implants

Syringe 1: Posterior capsule (8-10 injections medial and 8-10 injections lateral).

Syringe 2: Femur medial and lateral periosteum, posterior periosteum, suprapatellar/quadriceps tendon (20 injections).

Syringe 3: Tibia fat pad (5 injections); pes anserinus, medial collateral ligament, and gutter (15 injections).

Syringe 4: Circumferential periosteum (15-20 injections).

9.2.2. After implantation of definitive implants

Syringe 5: Midline quadriceps tendon (10 injections); retinaculum, medial gutter, femoral to tibia (10 injections).

Syringe 6: Lateral gutter, femoral to tibial (10 injections); subcutaneous/closure (10 injections).

Each injections should deliver approximately 1-1.5ml to the intended area. The tissue should visibly expand with minimal leakage.

© Copyright: The University of Oxford and Oxford University Hospitals NHS Trust 2016

9.3. Storage of IMP

Dependent on the standard practice and/or preference at each site, liposomal bupivacaine will be stored either in pharmacy or theatre refrigerators, temperature stipulated between 2°C and 8°C. Once removed from the refrigerator the drugs may be stored unopened at room temperature (20°C to 25°C) for up to 30 days but they should not be re-refrigerated. Vials should be visually inspected before use and must not be used if the stopper is bulging. Once opened, it must be used within 4 hours.

Bupivacaine hydrochloride should not be refrigerated or frozen as per the SmPC.

The appropriate pharmacy and/or theatre staff at all sites will be responsible for ensuring the drugs are kept at the correct temperature and where storage conditions are noted to be outside the recommended limits, the vials would need to be quarantined and disposed of as per standard practice at each site. Sites will be permitted to use their own temperature logs or one can be provided by the Oxford study office.

9.4. Compliance with Trial Treatment

The trial and control drugs are administered as a single intra-operative dose via peri-articular infiltration. As such, patient compliance with the treatment is not relevant in this study. However, surgical compliance with the intervention will be monitored on the Surgery CRF. Each treatment dose will be diluted to the same volume using normal saline as per the manufacturer's instructions. The injection technique of administration will be standardised and compliance with this confirmed on the Surgery CRF. Reasons for any deviation from the administration technique will also be recorded. The amount of drug administered will be recorded.

9.5. Accountability of the Trial Treatment

Accurate records of liposomal bupivacaine shipments, vials dispensed and destroyed must be maintained using the IMP Accountability Log provided in the Pharmacy File. This inventory record must be available for inspection at any time by the Trial Office. IMP supplies are to be used only in accordance with this protocol and under the supervision of the Investigator.

Accountability for the control drug will not be requested or monitored by the Trials Office.

9.6. Concomitant Medication

The trial and control drug are locally acting and no systemic interactions with other drugs have been reported. Local research teams will record any pain relief medication administered post-operatively. The opportunity to record any variants of the standard medications listed below will also be provided on the CRFs. Sites are not required to record concomitant medication outside of those listed below. Both the trial and control drug will be made up to volume using saline 0.9%. Liposomal bupivacaine must not be diluted with water or other hypotonic agents as it will result in disruption of the liposomal particles.

9.6.1. Post-Operative Analgesia

Post-operative opioid consumption will be captured on the In-patient CRF. The management of post-operative pain may vary across each participating centre. Local post-operative analgesic regimes must be discussed and approved by the Chief Investigator prior to site activation.

© Copyright: The University of Oxford and Oxford University Hospitals NHS Trust 2016

9.7. Post-trial Treatment

The trial and control drugs are administered as a single intra-operative dose via peri-articular infiltration and no further doses of either of the drugs will be administered during the study period.

10. SAFETY REPORTING

10.1. Definitions

Adverse Event (AE)	Any untoward medical occurrence in a participant to whom a medicinal product has been administered, including occurrences which are not necessarily caused by or related to that product.
Adverse Reaction (AR)	An untoward and unintended response in a participant to an investigational medicinal product which is related to any dose administered to that participant.<br>The phrase "response to an investigational medicinal product" means that a causal relationship between a trial medication and an AE is at least a reasonable possibility, i.e. the relationship cannot be ruled out.<br>All cases judged by either the reporting medically qualified professional or the Sponsor as having a reasonable suspected causal relationship to the trial medication qualify as adverse reactions.
Serious Adverse Event (SAE)	A serious adverse event is any untoward medical occurrence that:<br>• results in death<br>• is life-threatening<br>• requires inpatient hospitalisation or prolongation of existing hospitalisation<br>• results in persistent or significant disability/incapacity<br>• consists of a congenital anomaly or birth defect.<br>Other ‘important medical events’ may also be considered serious if they jeopardise the participant or require an intervention to prevent one of the above consequences.<br>NOTE: The term "life-threatening" in the definition of "serious" refers to an event in which the participant was at risk of death at the time of the event; it does not refer to an event which hypothetically might have caused death if it were more severe.
Serious Adverse Reaction (SAR)	An adverse event that is both serious and, in the opinion of the reporting Investigator, believed with reasonable probability to be due to one of the trial treatments, based on the information provided.
Suspected Unexpected Serious Adverse Reaction	A serious adverse reaction, the nature and severity of which is not consistent with the information about the medicinal product in

© Copyright: The University of Oxford and Oxford University Hospitals NHS Trust 2016

(SUSAR)	question set out:<br>• in the case of a product with a marketing authorisation, in the summary of product characteristics (SmPC) for that product<br>• in the case of any other investigational medicinal product, in the investigator's brochure (IB) relating to the trial in question.

NB: to avoid confusion or misunderstanding of the difference between the terms "serious" and "severe", the following note of clarification is provided: "Severe" is often used to describe intensity of a specific event, which may be of relatively minor medical significance. "Seriousness" is the regulatory definition supplied above.

Any pregnancy occurring during the clinical trial and the outcome of the pregnancy should be recorded and followed up for congenital abnormality or birth defect, at which point it would fall within the definition of "serious".

10.2. Causality

The relationship of each SAE to the trial medication must be determined by a medically qualified individual according to the following definitions:

Reasonable possibility: The adverse event follows a reasonable temporal sequence from trial medication administration. It cannot reasonably be attributed to any other cause.

No reasonable possibility: The adverse event is probably produced by the participant's clinical state or by other modes of therapy administered to the participant.

Assessment of causality (i.e. relatedness) will be recorded in the SAE form by the site PI or a delegate (medically qualified doctor). In addition, this will be independently reviewed by a nominated clinician.

10.2.1 Events related to IMP administration during surgery

Expected events related to the administration of the trial drug (EXPAREL) into surgical sites have been determined according to the FDA approved US Prescribing Information, found in the Appendix A of the IMPD or here: www.dailymed.nlm.nih.gov. This will be used as the Reference Safety Information (RSI) for all safety assessments and reporting, including SUSARs.

Reported AEs occur at a similar rate as the corresponding bupivacaine hydrochloride controls in the active comparator studies.

The surgery and the administration of the drugs are integrated components of the treatment arms. Events may not be linked to one specific component, however, those expected events related to knee replacement surgery are discussed in 10.2.2.

The most common adverse reactions (incidence greater than or equal to 10%) following EXPAREL administration in surgical sites were nausea, constipation, and vomiting.

The common adverse reactions (incidence greater than or equal to 2% to less than 10%) following EXPAREL administration were pyrexia, dizziness, edema peripheral, anemia, hypotension, pruritus,

© Copyright: The University of Oxford and Oxford University Hospitals NHS Trust 2016

tachycardia, headache, insomnia, anemia postoperative, muscle spasms, hemorrhagic anemia, back pain, somnolence, and procedural pain.

The less common/rare adverse reactions (incidence less than 2%) following EXPAREL administration were chills, erythema, bradycardia, anxiety, urinary retention, pain, edema, tremor, dizziness postural, paresthesia, syncope, incision site edema, procedural hypertension, procedural hypotension, procedural nausea, muscular weakness, neck pain, pruritus generalized, rash pruritic, hyperhidrosis, cold sweat, urticaria, bradycardia, palpitations, sinus bradycardia, supraventricular extrasystoles, ventricular extrasystoles, ventricular tachycardia, hypertension, pallor, anxiety, confusional state, depression, agitation, restlessness, hypoxia, laryngospasm, apnea, respiratory depression, respiratory failure, body temperature increased, blood pressure increased, blood pressure decreased, oxygen saturation decreased, urinary incontinence, vision blurred, tinnitus, drug hypersensitivity, and hypersensitivity.

10.2.2 Events Related to Knee Replacement Surgery

There will be many events expected in this patient population that are related specifically to their knee replacement surgery and the perioperative care they receive. All knee replacement procedures whether primary surgery or revision procedures carry a risk of death, morbidity including wound infection, bleeding intra and post operatively, thrombo-embolic complications and complications secondary to existing co-morbidity e.g. ischaemic heart disease [31].

Specific complications following knee replacement procedures include loosening of components – Tibia/Femur/both, dislocation of knee/bearing, superficial and deep infection, unexplained knee pain, knee stiffness, haematoma, mechanical failure of replacement, periprosthetic fracture. These complications may result in the need for further surgery such as revision operations, arthroscopy, washout, manipulation under anaesthetic, debridement (open), aspiration, above knee amputation, patella resurfacing [31].

Any expected complications outlined above will be documented on the Adverse Event form. If any complications become serious (i.e. meets the SAE criteria) the SAE CRF will be completed accordingly.

All SAEs will be coded using MedDRA. SAEs related to knee replacement surgery will be coded as a post-operative complication.

10.3. Procedures for Recording Adverse Events & Reporting Serious Adverse Events

Within 30 days of the surgery, Adverse Events need to be recorded if there is a reasonable possibility they are related to the:

- Administration of the IMP or control drug
- Knee replacement surgery

These will be recorded on Adverse Event form.

If the Adverse Event is deemed to be serious (i.e. meets the SAE criteria) then this will be reported on the SAE form. Serious Adverse Events must be reported throughout the duration of the study and follow up period (12months). The sites must notify the study office in Oxford of these events within 24 hours

© Copyright: The University of Oxford and Oxford University Hospitals NHS Trust 2016

following knowledge of the event. The completed form will be sent to the Oxford study office via secure nhs.net email (situ.oxford@nhs.net).

All patient deaths, regardless of cause/relatedness, will need to be reported on the SAE form.

Centres will routinely monitor patient records for any events that may have been missed.

The study office in Oxford will perform an initial check of SAE reports, request any additional information, and ensure it is reviewed by the trial's nominated person in a timely manner and according to the relevant SOP on safety reporting. The nominated person will also perform an independent review of the causality.

SAEs considered related to the trial medication as judged by a medically qualified investigator or the Sponsor will be followed either until resolution, or when the *event* is considered stable.

10.4. Assessment of Expectedness

The RSI (US Prescribing Information, found in the Appendix A of the IMPD or here: www.dailymed.nlm.nih.gov) lists all known expected events in relation to the administrations of the trial drug (EXPAREL) and the control (bupivacaine hydrochloride) during surgery (see section 10.2.1 above).

Assessment of expectedness will be performed against what is documented in the RSI and will be done centrally and independent of the site's PI.

If during the trial, a non-serious expected adverse reaction listed in the RSI is observed as serious then this will also be reported as a SUSAR. In addition, in the unlikely event that an adverse reaction is fatal or life-threatening this will also be reported as SUSAR.

10.5. SUSAR Reporting

All SUSARs will be processed by the Oxford study office to the relevant Competent Authority, REC, Sponsor QA office and other parties as applicable. For fatal and life-threatening SUSARS, this will be done no later than 7 calendar days after the Sponsor or delegate is first aware of the reaction. Any additional relevant information will be reported within 8 calendar days of the initial report. All other SUSARs will be reported within 15 calendar days.

Principal Investigators will be informed of all SUSARs for the relevant IMP for all studies with the same Sponsor, whether or not the event occurred in the current trial.

10.6. Data Safety and Monitoring Committee & Trial Steering Committee

A Data Safety and Monitoring Committee (DSMC) will be appointed to safeguard the interests of the trial participants, to assess the safety and efficacy of the interventions during the trial, and to monitor the overall conduct of the trial, protecting its validity and credibility. The DSMC will be independent of the trial investigators and sponsor, and will adopt a DAMOCLES charter that defines its terms of reference and operation in relation to oversight of the trial. It will meet at least every 12 months over the duration of the trial. The DSMC will not be asked to perform any formal interim analyses of effectiveness. It will, however, review accruing data and summaries of that data presented by the treatment group and will assess the screening algorithm against the eligibility criteria. It will also consider emerging evidence from

© Copyright: The University of Oxford and Oxford University Hospitals NHS Trust 2016

other related trials or research and review any related SAEs that have been reported. The DSMC may advise the chair of the Trial Steering Committee (TSC) at any time if, in its view, the trial should be stopped for ethical reasons, including concerns about participant safety or clear evidence of the effectiveness of one of the treatments. The DSMC will comprise of two independent medically qualified clinicians and a statistician.

In addition to a DSMC, a TSC will be appointed whose primary function is to act as an oversight body for the trial on behalf of the Sponsor and funding body. The TSC will be chaired by an independent member and will consider and act, as appropriate, upon the recommendations of the DSMC and ultimately carries the responsibility of deciding whether or not the trial needs to be stopped on the grounds of safety. The TSC will adopt a charter that defines its terms of reference and operation in relation to the oversight of the trial and will meet at least every 12 months over the duration of the study.

The Sponsor QA office will be provided will the trial committee charters and minutes.

10.7. Development Safety Update Reports

Where appropriate, the trial management team will submit Development Safety Update Reports (DSURs). These will be submitted (in addition to the expedited reporting above) once a year throughout the clinical trial, or on request, to the Competent Authority (Medicines and Healthcare products Regulatory Agency (MHRA) in the UK), Ethics Committee, Host NHS Trust and Sponsor.

11. STATISTICS

11.1. Description of Statistical Methods

This section provides a brief summary of the statistical and cost effectiveness analysis with full details being included in separate stand-alone Statistical Analysis Plan (SAP) and Health Economic Analysis Plans (HEAP). These will be drafted early in the trial and finalised prior to the final datalock and any formal analysis of primary outcomes.

The primary objective of improved patient recovery will be assessed by analysing the two primary endpoints: global QoR-40 scores at 72 hours and cumulative pain score from 6 to 72 hours following surgery. The QoR-40 is a global measure of quality of recovery. It incorporates five dimensions of health: patient support, comfort, emotions, physical independence, and pain; each item is graded on a five-point Likert scale. QoR-40 scores range from 40 (extremely poor quality of recovery) to 200 (excellent quality of recovery) [32]. The null hypothesis that there is no difference between the treatment arms will be rejected if either of these outcomes are statistically different at the 2.5% significance level. The trial will be considered to have a positive result if either of the dual primary outcomes return a positive result.

Pain scores will be assessed daily in the evening at approximately 6, 24, 48 and 72 hours post-surgery, using a 0-10 VAS and cumulative pain scores will be calculated as area under the curve.

Both of the primary outcomes will be analysed using multivariate linear regression adjusting for stratification factors and other important prognostic variables, including baseline scores in the case of the QoR-40. The adjusted mean difference between the two groups together with 97.5% confidence intervals will be reported for each of the primary outcomes. If either of the primary outcomes is not

© Copyright: The University of Oxford and Oxford University Hospitals NHS Trust 2016

normally distributed, transformation to normality will be the first approach. If this is not possible, then non-parametric techniques without adjustment will be used.

As secondary analyses both pain and QoR-40 measured at 6, 24, 48 and 72 hours will be assessed using longitudinal methods to take account of the multiple-time points and the correlation between them. Multi-level, multivariate linear regression modelling with adjustment for the same factors as the primary analysis will be used. Other continuous variables will be analysed using similar methodology. For binary variables the number and percentage of patients in each category will be reported for each treatment group and overall. Chi-squared tests will be utilised for comparing the treatments, with additional analyses being undertaken using multi-variable logistic regression with adjustment for stratification and other important prognostic factors if sufficient events have been observed.

Reporting of the results will be based on the CONSORT statement.

It is anticipated that all statistical analysis will be undertaken using Stata (StataCorp LP, www.stata.com) or other well-validated statistical packages.

Health Economic Analysis: A cost utility analysis will also be performed using quality-adjusted life years (QALYs) as the main health outcome, obtained using the EuroQol EQ5D-5L questionnaire at baseline, 72 hours, 6 weeks, 6 months and 1 year. The perspective of the analysis will be societal and costs will be informed by collecting data on the initial surgery, cost of treatment and analgesic medication, primary/hospital and social care resource use over the first year following surgery, out-of-pocket expenses (e.g. over the counter medications, travel costs to attend consultations, private practitioners, etc.), unpaid care from family members or friends and loss of earnings resulting from knee replacement. Such data will be collected from hospital medical records and using self-completed patient questionnaires at 6 weeks, 6 months and 12 months. Healthcare and social care utilisation will be valued using national databases [33, 34].

The results of the cost utility analysis will be presented using an incremental cost-effectiveness ratio expressed as cost per QALY gained and interpreted as the additional costs/savings associated to the additional QALY benefits of Liposomal bupivacaine compared to Bupivacaine hydrochloride.

11.2. The Number of Participants

The trial has been powered for two primary end-points, QoR-40 at 72 hours and cumulative pain score from 6-72 hours, with adjustment for multiplicity using the Bonferroni method [35]. The trial will be assessed as providing evidence of a difference if either of the two primary end-points is statistically and clinically significant.

The study requires 240 patients per treatment arm in order to be 90% powered to detect a 5 point difference in global QoR-40 score between groups at a significance level of 0.025 (2-sided, adjusted for multiplicity) assuming that the standard deviation is 15.5 [36]. To allow for 4% loss to follow-up this has been inflated to 500 patients (250 per treatment arm).

The study also requires a minimum of 225 patients per treatment arm in order to be 90% powered to detect a standardised difference of 33% between groups in cumulative pain score calculated as area under the curve from 6 to 72 hours post-surgery at a significance level of 0.025 (2-sided, adjusted for

© Copyright: The University of Oxford and Oxford University Hospitals NHS Trust 2016

multiplicity). Inflating the sample size to 500 patients (250 per treatment arm) will allow for 10% loss to follow-up on this variable.

Therefore a total of 500 patients will be randomised (250 per arm).

Randomisation between intervention and control will be 1:1, and will be stratified by centre and operation type (UKR or TKR). It is expected that a maximum of 10% will be UKR. Results of this trial will then be generalisable with the population of patients undergoing UKR, as well as those undergoing TKR, in the UK, which is currently 8%.

11.3. The Level of Statistical Significance

For the dual primary outcomes, a significance level of 0.025 (2.5%) will be used and 97.5% confidence intervals will be presented. All secondary analyses will be considered as supporting the primary analyses, and thus, a significance level of 0.05 (5%) will be used and 95% confidence intervals will be presented.

11.4. Procedure for Accounting for Missing, Unused, and Spurious Data

Missing data will be minimized. Missing data will be reported and summarized by treatment arm. The distribution of missing data will be explored to assess the assumption that data are missing at random and multiple imputation will be utilized as appropriate with sensitivity analyses undertaken to explore the missing assumptions.

A more detailed account of analysis will be available in the statistical analysis plan and the health economic analysis plan.

Intention-to-treat analysis will be used with all patients will analysed in the group they were randomised to, regardless of treatment actually received. Sensitivity analyses will be carried out on a per-protocol population, to be fully defined in the statistical analysis plan.

11.5. Procedures for Reporting any Deviation(s) from the Original Statistical Plan

Any changes or deviations from the original SAP will be described and justified in the protocol, final report and/or publications, as appropriate.

12. DATA MANAGEMENT AND SHARING PLAN

All data will be processed according to the Data Protection Act 1998 and all documents will be stored safely in confidential conditions. A Data Management and Sharing Plan will be produced for the trial and will include reference to confidentiality, access and security arrangements. All trial-specific documents, except for the signed consent form and follow-up contact details, will refer to the participant with a unique study participant number/code and not by name. Participant identifiable data will be stored separately from study data and in accordance with OCTRU SOPs. All trial data will be stored securely in offices only accessible by swipe card by the central coordinating team staff in Oxford and authorised personnel.

© Copyright: The University of Oxford and Oxford University Hospitals NHS Trust 2016

13. QUALITY ASSURANCE PROCEDURES

The CTU conducted a risk assessment prior to the study starting. Issues raised have been addressed within the final protocol and procedures have been planned to monitor the ongoing risks of the trial. A risk proportionate approach will be utilised within this trial. Central monitoring of trial procedures will be imbedded into the trial conduct and management. The trial will be subject to audit according to OCTRU's Audit Programme. It will undergo a process of review before the trial is granted the green light to begin recruiting patients.

14. SERIOUS BREACHES

The Medicines for Human Use (Clinical Trials) Regulations contain a requirement for the notification of "serious breaches" to the MHRA within 7 days of the Sponsor becoming aware of the breach.

A serious breach is defined as "A breach of GCP or the trial protocol which is likely to affect to a significant degree –

(a) the safety or physical or mental integrity of the subjects of the trial; or

(b) the scientific value of the trial".

In the event that a serious breach is suspected, the study office in Oxford must inform the Sponsor within 1 calendar day. The potential serious breach will be reviewed by the Sponsor and, if appropriate, the study office in Oxford will be responsible for onward reporting to the REC, MHRA and the NHS host organisation within seven calendar days. A detailed outline of this procedure will be available as a Trial Specific Instruction in the TMF.

15. ETHICAL AND REGULATORY CONSIDERATIONS

15.1. Declaration of Helsinki

The Investigator will ensure that this trial is conducted in accordance with the principles of the Declaration of Helsinki.

15.2. Guidelines for Good Clinical Practice

The Investigator will ensure that this trial is conducted in accordance with relevant regulations and with Good Clinical Practice.

15.3. Approvals

Having undergone Sponsor QA review and approval, the protocol, Informed Consent Form, Participant Information Sheet and any proposed advertising material will be submitted to the Health Research Authority (HRA), an appropriate REC, MHRA and host institution(s) for written approval.

© Copyright: The University of Oxford and Oxford University Hospitals NHS Trust 2016

The Investigator will submit and, where necessary, obtain approval from the above parties for all substantial amendments to the original approved documents.

15.4. Reporting

The CI will submit once a year throughout the clinical trial, or on request, an Annual Progress Report and DSUR to the MHRA, REC, host organisation and Sponsor. In addition, an End of Trial notification and final report will be submitted to the MHRA, the REC, host organisation and Sponsor.

The NIHR RfPB, as the study funder, will also require annual reports on study progress. The CI will be responsible for providing this.

15.5. Participant Confidentiality

The trial staff will ensure that the participants' anonymity is maintained. The participants will be identified only by a participant ID number on all trial documents and any electronic database. All documents including questionnaires, will be stored securely and only accessible by trial staff and authorised personnel. The trial will comply with the Data Protection Act, which requires data to be anonymised as soon as it is practical to do so. If patients are discharged early and telephone communication is required, consent will be obtained to do so.

Consent will be obtained from patients for personal information to be sent to the central study team in Oxford (ie: name, address, email, telephone number). This information is required to coordinate the postal follow up at 12 months.

15.6. Expenses and Benefits

Reasonable travel expenses for any visits additional to normal care will be reimbursed on production of receipts, or a mileage allowance provided as appropriate.

16. FINANCE AND INSURANCE

16.1. Funding

This study is funded by a Research for Patient Benefit (RfPB) grant from the National Institute for Health Research (NIHR). Ref# PB-PG-0215-36084.

16.2. Insurance

As the Sponsor, the University of Leeds are able to provide insurance to cover for liabilities and prospective liabilities arising from negligent harm. They are able to provide insurance cover, in certain circumstances, for claims arising from non-negligent harm including the design of the protocol. Clinical negligence indemnification will rest with the participating NHS Trust or Trusts under standard NHS arrangements.

17. PUBLICATION POLICY

© Copyright: The University of Oxford and Oxford University Hospitals NHS Trust 2016

Authorship will be in line with the International Committee of Medical Journal Editors recommendations. Namely that authorship be based on the following 4 criteria:

- Substantial contributions to the conception or design of the work; or the acquisition, analysis, or interpretation of data for the work; AND
- Drafting the work or revising it critically for important intellectual content; AND
- Final approval of the version to be published; AND
- Agreement to be accountable for all aspects of the work in ensuring that questions related to the accuracy or integrity of any part of the work are appropriately investigated and resolved.

Contributors who meet fewer than all four of the above criteria for authorship will be acknowledged as contributors to any publications.

© Copyright: The University of Oxford and Oxford University Hospitals NHS Trust 2016

18. APPENDIX A: TRIAL FLOW CHART

Inclusion (see Section 7.2 for full details)

- Unilateral primary knee replacement, including both total knee replacement (TKR) or unicompartmental knee replacement (UKR), for end stage osteoarthritis
- American Society of Anaesthesiologists (ASA) Grade I to III
- Willing and able to consent for themselves
- Male or Female, aged 18 or above
- Able and willing to comply with all trial requirements

Recruitment

Exclusion (see Section 7.3 for full details)

- Allergy or intolerance to amide-type local anesthetics
- Nerve damage in affected limb
- Rheumatoid arthritis
- Any significant disease, disorder or condition which may put the participant at risk
- Involvement in other research
- Significant cognitive impairment or language issues
- Contra-lateral knee replacement within the trial or within 12 months prior to randomisation

Baseline Assessments

Patient demographics
Summary medical history
Summary medication history
QoR-40
Pain VAS at rest
Oxford Knee Score
American Knee Society Score
Summary healthcare & social services use
Summary employment history
EQ-5D-5L

Randomisation

n=500

N=250 | N=250

Allocation

Liposomal bupivacaine + bupivacaine hydrochloride (intervention)

Bupivacaine hydrochloride (control)

Operation

Drug administered as single intra-operative peri-articular injection as part of multimodal analgesia

Follow Up

0 to 72 hours following surgery i.e. days 0, 1, 2 & 3 (in-patient questionnaires)

QoR-40
Pain VAS at rest
Cumulative opiate use
EQ-5D-5L
SAEs, if applicable

6 weeks (postal questionnaire)

Summary medication history
QoR-40
Pain VAS at rest
Oxford Knee Score
American Knee Society Score
Summary healthcare & social services use
Summary employment history
EQ-5D-5L
SAEs, if applicable

6 months (postal questionnaire) – same as 6 weeks (see above)

1 year (postal questionnaire)

Summary medication history
QoR-40
Pain VAS at rest
Oxford Knee Score
Summary healthcare & social services use
Summary employment history
EQ-5D-5L
American Knee Society score (function only)

© Copyright: The University of Oxford and Oxford University Hospitals NHS Trust 2016

19. APPENDIX B: ASSESSMENT TABLE

Assessment		Timing								
		Baseline	Operation	Post-op Day 0	Post-op Day 1	Post-op day 2	Post-op day 3	6 weeks (+/- 2 weeks)	6 months (+/- 6 weeks)	1 year (+/-1 month)
**Patient demographics** (gender, age at surgery, body mass index)	Informed Consent	✓								
**Summary medical history**		✓								
**Summary medication history** (current medication and duration of current medication, changes to current medication)		✓						✓	✓	✓
**QoR 40**		✓		✓	✓	✓	✓	✓	✓	✓
**Pain VAS (0-10) at rest**		✓		✓	✓	✓	✓	✓	✓	✓
**Cumulative opiate use**				✓	✓	✓	✓			
**Oxford Knee Score**		✓						✓	✓	✓
**American Knee Society Score**		✓						✓	✓	✓ (function only)
**Summary healthcare/social services use and informal care due to knee** (GP visits, outpatient visits, allied healthcare contacts, hospital admissions, medications, meals on wheels, and unpaid care)		✓						✓	✓	✓
**Summary employment history** (in paid employment, not in employment, time away from work or usual activities)		✓						✓	✓	✓
**EQ-5D-5L**		✓		✓	✓	✓	✓	✓	✓	✓
**Treatment**			✓							

© Copyright: The University of Oxford and Oxford University Hospitals NHS Trust 2016

20. APPENDIX C: AMENDMENT HISTORY

Amendment No.	Protocol Version No.	Date issued	Author(s) of changes	Details of Changes made
	V1.1	10Apr2017	Ivy Raymundo	Changes made in response to initial comments from the MHRA
	V2.0	11May2017	Ivy Raymundo	New toxicological or pharmacovigilance data or new interpretation of toxicological or pharmacovigilance data
	V3.0	11Apr2018	Lisa Poulton	Rewording of inclusion criteria, changes to section 11: statistics and other minor amendments
	V4.0	11Sep2018	Lisa Poulton	Revision of contra-lateral knee exclusion criteria so that only patients who have had surgery within 12 months of the trial are excluded
	V5.0	26Nov2018	Lisa Poulton	Removal of ASA III exclusion
	V6.0	03Feb2020	Lisa Poulton	Rewording of safety secondary endpoint and other minor administrative changes/clarifications

Protocol amendments must be submitted to the Sponsor for approval prior to submission to the REC committee or MHRA.

© Copyright: The University of Oxford and Oxford University Hospitals NHS Trust 2016

21. REFERENCES

1. Philips, D.M., *JCAHO pain management standards are unviled. Joint Commission on Accreditation of Healthcare Organizations.* JAMA, 2000. **284**(4): p. 428-9.
2. *Practice guidelines for acute pain management in the perioperative setting: an updated report by the American Society of Anesthesiologists Task Force on Acute Pain Management.* Anesthesiology, 2012. **116**(2): p. 248-73.
3. Carr, D.B., *The development of national guidelines for pain control: synopsis and commentary.* Eur J Pain, 2001. **5 Suppl A**: p. 91-8.
4. Chan, E.Y., et al., *Postoperative pain following hospital discharge after knee replacement surgery: a patient survey.* Pain Manag, 2013. **3**(3): p. 177-88.
5. Apfelbaum, J.L., et al., *Postoperative pain experience: results from a national survey suggest postoperative pain continues to be undermanaged.* Anesth Analg, 2003. **97**(2): p. 534-40, table of contents.
6. Moskovitz, B.L., et al., *Analgesic treatment for moderate-to-severe acute pain in the United States: patients' perspectives in the Physicians Partnering Against Pain (P3) survey.* J Opioid Manag, 2011. **7**(4): p. 277-86.
7. Gottschalk, A., et al., *Prospective evaluation of pain and analgesic use following major elective intracranial surgery.* J Neurosurg, 2007. **106**(2): p. 210-6.
8. *Top Ten Priorities for Hip and Knee Replacement*. 2014 [cited 2014 23/07/2014]; Available from: http://www.lindalliance.org.
9. Savaridas, T., et al., *Reduced medium-term mortality following primary total hip and knee arthroplasty with an enhanced recovery program. A study of 4,500 consecutive procedures.* Acta Orthop, 2013. **84**(1): p. 40-3.
10. Liu, S.S. and C.L. Wu, *The effect of analgesic technique on postoperative patient-reported outcomes including analgesia: a systematic review.* Anesth Analg, 2007. **105**(3): p. 789-808.
11. Liu, S.S., B.M. Block, and C.L. Wu, *Effects of perioperative central neuraxial analgesia on outcome after coronary artery bypass surgery: a meta-analysis.* Anesthesiology, 2004. **101**(1): p. 153-61.
12. Nishimori, M., J.C. Ballantyne, and J.H. Low, *Epidural pain relief versus systemic opioid-based pain relief for abdominal aortic surgery.* Cochrane Database Syst Rev, 2006(3): p. CD005059.
13. Kehlet, H. and J.B. Dahl, *The value of "multimodal" or "balanced analgesia" in postoperative pain treatment.* Anesth Analg, 1993. **77**(5): p. 1048-56.
14. BootsWebMD. *RxList*. Marcain 2016; Available from: http://www.rxlist.com/marcaine-drug/clinical-pharmacology.htm.
15. Bergese, S.D., et al., *Efficacy profile of liposome bupivacaine, a novel formulation of bupivacaine for postsurgical analgesia.* J Pain Res, 2012. **5**: p. 107-16.
16. Xu, C.P., et al., *Efficacy and safety of single-dose local infiltration of analgesia in total knee arthroplasty: A meta-analysis of randomized controlled trials.* Knee, 2014.
17. Angst, M.S. and D.R. Drover, *Pharmacology of drugs formulated with DepoFoam: a sustained release drug delivery system for parenteral administration using multivesicular liposome technology.* Clin Pharmacokinet, 2006. **45**(12): p. 1153-76.
18. Barrington, J., T. Hamilton, Editor. 2014: *Texas Center for Joint Replacement, Plano, Texas*.
19. Berend, K., T. Hamilton, Editor. 2014: *Joint Implant Surgeons Inc., New Albany, Ohio*.
20. Barrington, J. *Liposomal Bupivicaine: The First 1,000 Cases in a New Era*. in *American Association of Orthopaedic Surgeons Annual Proceedings 2014* 2014.
21. Allen, T.M. and P.R. Cullis, *Liposomal drug delivery systems: from concept to clinical applications.* Adv Drug Deliv Rev, 2013. **65**(1): p. 36-48.
22. Hamilton, T.W., et al., *Liposomal bupivacaine peripheral nerve block for the management of postoperative pain.* Cochrane Database Syst Rev, 2016(8): p. CD011476.
23. Hamilton, T., et al., *Liposomal bupivacaine infiltration at the surgical site for the management of postoperative pain.* Cochrane Database Syst Rev, 2017(2): p. CD011419.

© Copyright: The University of Oxford and Oxford University Hospitals NHS Trust 2016

24. Herrera, F.J., J. Wong, and F. Chung, *A systematic review of postoperative recovery outcomes measurements after ambulatory surgery.* Anesth Analg, 2007. **105**(1): p. 63-9.
25. Dawson, J., et al., *Questionnaire on the perceptions of patients about total knee replacement.* J Bone Joint Surg Br, 1998. **80**(1): p. 63-9.
26. Scuderi, G.R., et al., *The New Knee Society Knee Scoring System.* Clinical Orthopaedics and Related Research, 2012. **470**(1): p. 3-19.
27. Clavien, P.A., et al., *The Clavien-Dindo classification of surgical complications: five-year experience.* Ann Surg, 2009. **250**(2): p. 187-96.
28. Pacira Pharmaceuticals, I., *Investigator's Brochure, Exparal (bupivacaine liposome injectable suspension)*. Current version.
29. Arthur, G.R., H.S. Feldman, and B.G. Covino, *Comparative pharmacokinetics of bupivacaine and ropivacaine, a new amide local anesthetic.* Anesth Analg, 1988. **67**(11): p. 1053-8.
30. Scott, D.B., et al., *Acute toxicity of ropivacaine compared with that of bupivacaine.* Anesth Analg, 1989. **69**(5): p. 563-9.
31. Beard, D., Price, A., Cook, J., Fitzpatrick, R., Carr, A., Campbell, M., Doll, H., Campbell, H., Arden, N., Cooper, C., Davies, L., Murray, D., *Total or Partial Knee Arthroplasty Trial - TOPKAT: study protocol for a randomised controlled trial.* (1745-6215 (Electronic)).
32. Myles, P.S., et al., *Development and psychometric testing of a quality of recovery score after general anesthesia and surgery in adults.* Anesth Analg, 1999. **88**(1): p. 83-90.
33. Curtis, L., *Costs of Health and Social Care.*, U. Personal Social Services Research Unit, Editor. 2014.
34. Health, D.o. *National Schedule of Reference Costs - Year 2012-13 - NHS trusts and NHS foundation trusts*. 2013 [cited 2014 20 June 2014]; Available from: https://www.gov.uk/government/publications/nhs-reference-costs-2012-to-2013.
35. Hochberg, Y. and A. Tamhane, *Multiple comparison procedures.* 1987.
36. Poitras, S., P.E. Beaule, and G.F. Dervin, *Validity of A Short-Term Quality of Life Questionnaire in Patients Undergoing Joint Replacement: The Quality of Recovery–40.* J Arthroplasty, 2012. **27**(9): p. 1604-1608. e1.

© Copyright: The University of Oxford and Oxford University Hospitals NHS Trust 2016

Study of Peri-Articular Anaesthetic for Replacement of the Knee

The clinical and cost effectiveness of peri-articular liposomal bupivacaine with bupivacaine hydrochloride compared to bupivacaine hydrochloride alone for post-operative recovery after knee replacement surgery: A multi-centre, patient-blinded, randomised controlled trial.

Statistical Analysis Plan

Version 2.0 – 17Dec2020

Based on Protocol version 6.0 – 03Feb2020

Trial registration: ISRCTN 54191675

Role	Name	Title	Signature	Date
Author	Ariel Wang	Medical Statistician	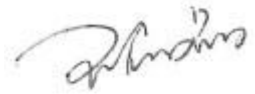	17Dec2020
Senior Statistician	Ruth Knight	Senior Medical Statistician		
Chief Investigator	Hemant Pandit	Chief Investigator		

Oxford Clinical Trials Research Unit (OCTRU)
Centre for Statistics in Medicine (CSM)

CONTENTS

1. INTRODUCTION ... 3
- 1.1 KEY PERSONNEL ... 3
- 1.2 CHANGES FROM PREVIOUS VERSION OF SAP ... 4

2. BACKGROUND AND OBJECTIVES ... 5
- 2.1 BACKGROUND AND RATIONALE ... 5
- 2.2 OBJECTIVES ... 5

3. STUDY METHODS ... 7
- 3.1 TRIAL DESIGN/FRAMEWORK ... 7
- 3.2 RANDOMISATION AND BLINDING ... 7
- 3.3 SAMPLE SIZE ... 7
- 3.4 STATISTICAL INTERIM ANALYSIS, DATA REVIEW AND STOPPING GUIDELINES ... 8
- 3.5 TIMING OF FINAL ANALYSIS ... 8
- 3.6 BLINDED ANALYSIS ... 8
- 3.7 STATISTICAL ANALYSIS OUTLINE ... 8

4. STATISTICAL PRINCIPLES ... 9
- 4.1 STATISTICAL SIGNIFICANCE AND MULTIPLE TESTING ... 9
- 4.2 DEFINITION OF ANALYSIS POPULATIONS ... 9

5. TRIAL POPULATION AND DESCRIPTIVE ANALYSES ... 9
- 5.1 REPRESENTATIVENESS OF STUDY SAMPLE AND PATIENT THROUGHPUT ... 9
- 5.2 WITHDRAWAL FROM TREATMENT AND/OR FOLLOW-UP ... 11
- 5.3 BASELINE COMPARABILITY OF RANDOMISED GROUPS ... 11
- 5.4 UNBLINDING ... 12
- 5.5 DESCRIPTION OF COMPLIANCE WITH INTERVENTION ... 12
- 5.6 RELIABILITY ... 14

6. ANALYSIS ... 14
- 6.1 OUTCOME DEFINITIONS ... 14
- 6.2 ANALYSIS METHODS ... 15
- 6.3 MISSING DATA ... 19
- 6.4 SENSITIVITY ANALYSIS ... 20
- 6.5 PRE-SPECIFIED SUBGROUP ANALYSIS ... 20
- 6.6 SUPPLEMENTARY/ ADDITIONAL ANALYSES AND OUTCOMES ... 20
- 6.7 HARMS ... 20
- 6.8 HEALTH ECONOMICS AND COST EFFECTIVENESS (WHERE APPLICABLE) ... 21
- 6.9 META-ANALYSES (IF APPLICABLE) ... 22

7. VALIDATION OF THE PRIMARY ANALYSIS ... 22

8. SPECIFICATION OF STATISTICAL PACKAGES ... 22

9. REFERENCES ... 23

APPENDIX: GLOSSARY OF ABBREVIATIONS ... 24

1. INTRODUCTION

This document details the proposed data presentation and analysis for the main paper(s) and final study reports from the ***Research for Patient Benefit (RfPB) funded, phase III, multi-centre, patient-blinded, randomised controlled trial assessing the clinical and cost effectiveness of peri-articular liposomal bupivacaine compared with bupivacaine hydrochloride alone for post-operative recovery after knee replacement surgery (SPAARK)***. The results reported in these papers should follow the strategy set out here. Subsequent analyses of a more exploratory nature will not be bound by this strategy, though they are expected to follow the broad principles laid down here. The principles are not intended to curtail exploratory analysis (for example, to decide cut-points for categorisation of continuous variables), nor to prohibit accepted practices (for example, data transformation prior to analysis), but they are intended to establish the rules that will be followed, as closely as possible, when analysing and reporting the trial. This document follows the published guidelines regarding the content of statistical analysis plans for clinical trials [1].

The analysis strategy will be available on request when the principal papers are submitted for publication in a journal. Suggestions for subsequent analyses by journal editors or referees, will be considered carefully, and carried out as far as possible in line with the principles of this analysis strategy; if reported, the source of the suggestion will be acknowledged.

Any deviations from the statistical analysis plan will be described and justified in the final report of the trial. The analysis should be carried out by an identified, appropriately qualified and experienced statistician, who should ensure the integrity of the data during their processing. Examples of such procedures include quality control and evaluation procedures.

1.1 Key personnel

Author(s) (Trial statistician(s))

Ruth Knight (until Jan2019)

ruth.knight@csm.ox.ac.uk

Jamie Stokes (Jan2019 – Oct2019)

jamie.stokes@ndorms.ox.ac.uk

Ariel Wang (Oct2019 – present)

ariel.wang@ndorms.ox.ac.uk

Reviewers (Trial Manager, DSMC, TSC, Statistician as appropriate)

Trial Manager

Lisa Poulton (until Feb2020)

Lisa.poulton@ndorms.ox.ac.uk

Yara Neves Silva (Feb2020 – present)

yara.nevessilva@ndorms.ox.ac.uk

DSMC

Dr Vassilis Athanassoglou (Chair)

Dr Rajesh Rout (Independent member)

Ms Katie Pike (Independent member)

TSC

Professor Hemant Pandit (Chief Investigator)

Mr Stephen McDonnell (Independent member)

Dr Milicia Bucknall (Independent member)

Dr Sabeena Sharma (Independent member)

Mr Simon Newman (Independent member)

Mrs Rosemary Wyber (Patient representative)

Mrs Rosalind Clow (Patient representative)

Approver (Senior Statistician, Chief Investigator)

Senior Statistician

Ines Rombach

Ines.rombach@ndorms.ox.ac.uk

Chief Investigator

Hemant Pandit

h.pandit@leeds.ac.uk

1.2 Changes from previous version of SAP

A summary of key changes from earlier versions of SAP, with particular relevance to protocol changes that have an impact on the design, definition, sample size, data quality/collection and analysis of the outcomes will be provided. Include protocol version number and date.

Version number<br>Issue date	Author of this issue	Protocol Version & Issue date	Significant changes from previous versions together with reasons
V1.0_24Mar2020	Ruth Knight	Protocol_V6.0_03Feb2020	Not applicable as this is the first issue
V2.0_17Dec2020	Ariel Wang	Protocol_V6.0_03Feb2020	Updates regarding the AKSS score and OKS score

2. BACKGROUND AND OBJECTIVES

2.1 Background and rationale

Around 100,000 primary knee replacements were performed in the UK last year and optimising the management of peri-operative pain and recovery has been identified as a patient priority [2]. It has been reported that up to three quarters of surgical patients receive inadequate pain relief, with 40% of patients reporting severe pain following knee replacement [3-6]. Optimising peri-operative pain management through the use of multi-modal analgesia reduces the surgical stress response and permits early rehabilitation whilst optimising recovery has many benefits both to the patients (reduced morbidity and mortality) and the healthcare system (reduced costs) [7]. Current pain relief strategies use opiate based analgesia; however, these drugs have significant side effects seen in up to 50% of patients. Opioid sparing techniques have been associated with enhanced patient satisfaction in the acute phase and there is increasing evidence about their longer term benefits.

The concept of multi-modal analgesia was introduced over twenty years ago and its use has expanded to many areas of surgery [8]. Multiple studies report the superiority of multi-modal analgesia over single agent therapy [9]. Local anaesthetic infiltration is commonly used as part of a multi-modal technique; however, the length of duration of action is a major limiting factor of current local anaesthetic techniques. Liposomal bupivacaine is liposome encapsulated bupivacaine which has been reported to be effective for up to 72 hours [10, 11]. Liposomal bupivacaine is not yet licensed in the UK for any indication; however it was licensed by the FDA in October 2011 and large centres are able to discharge patients on the day of surgery following knee and hip replacement with no reported incidents of increased readmission rates or wound issues [12-14]. A series of randomised controlled trials have investigated the use of Liposomal bupivacaine for post-operative pain; however, a recent Cochrane Review concluded that there was not sufficient evidence to support the use of liposomal bupivacaine in the management of post-operative pain following knee replacement [15]. Given the potential impact of liposomal bupivacaine, this multi-centre, patient-blinded RCT has been designed to assess the clinical and cost effectiveness of peri-articular liposomal bupivacaine and bupivacaine hydrochloride compared with peri-articular bupivacaine hydrochloride alone.

2.2 Objectives

The aim of this randomised controlled trial is to compare liposomal bupivacaine + bupivacaine hydrochloride versus bupivacaine hydrochloride alone on post-operative recovery assessed in terms of both systemic recovery as well as local recovery of the operated joint. The research hypothesis is that post-operative recovery will be better in those patients who receive liposomal bupivacaine + bupivacaine hydrochloride compared to those who receive bupivacaine hydrochloride alone. The primary and secondary endpoints and objectives for this study are as described in Table 1.

Table 1: Primary and secondary objectives and endpoints

	Objectives	Endpoints
**Primary**	Evaluate the effectiveness of liposomal bupivacaine with bupivacaine hydrochloride compared with bupivacaine hydrochloride alone on post-operative recovery. Post-operative recovery will be assessed both in terms of systemic recovery as well as local recovery of the operated joint	• Quality of Recovery 40 (QoR 40) Score at 72 hours.<br>• Cumulative daily pain score at rest using 0-10 Visual Analogue Scale (VAS) from 6 to 72 hours.
**Secondary**	To assess other markers of recovery both in the short term and the long term.	• Pain score measured using a 0-10 VAS at baseline and evening of surgery (day 0) and days 1, 2 and 3 following surgery and at 6 weeks, 6 months and 1 year post-randomisation.<br>• QoR 40 Score at baseline and evening of surgery (day 0) and days 1, 2 and 3 following surgery and at 6 weeks, 6 months and 1 year post-randomisation.<br>• Cumulative opioid consumption over 72 hours following surgery.<br>• Fitness for discharge against pre-defined criteria at evening of surgery (day 0) and days 1, 2 and 3 following surgery<br>• Functional outcome using validated, patient-reported (Oxford Knee Score) and qualitative (American Knee Society Score) outcome scores at baseline, 6 weeks, 6 months and 1 year post-randomisation.
	To evaluate cost-effectiveness compared with the current standard of care.	• Cost-utility analysis using patient-reported quality of life as the main outcome, obtained using the EuroQol EQ-5D-5L at baseline, 72 hours following surgery, 6 weeks, 6 months and 1 year post-randomisation.
	Safety	• Serious adverse events up to 12 months post-randomisation<br>• Related AEs up to 30 days post-surgery<br>• Intraoperative and inpatient complications.

3. STUDY METHODS

3.1 Trial Design/framework

The SPAARK trial is a patient-blinded, multi-centre, active comparator, randomised, controlled, two-arm, parallel group, superiority trial (RCT) comparing the effectiveness of liposomal bupivacaine hydrochloride + bupivacaine hydrochloride versus bupivacaine hydrochloride alone on post-operative recovery (systemic and local) in patients undergoing knee replacement surgery.

Liposomal bupivacaine hydrochloride is a local anaesthetic injected to provide long acting pain relief through encapsulating the active component (bupivacaine hydrochloride) within multi-vesicular liposomes. The intervention in this study is liposomal bupivacaine hydrochloride delivered together with bupivacaine hydrochloride, with a view of targeting both immediate and long lasting pain relief. This will be compared with a control of bupivacaine hydrochloride on its own.

3.2 Randomisation and Blinding

Randomisation between intervention and control will be 1:1, and will be stratified by study centre and operation type (Total Knee Replacement (TKR) or Unicompartmental Knee Replacement (UKR)). Randomisation will be performed using the RRAMP internet system provided by the Oxford Clinical Trials Research Unit (OCTRU). Where possible, randomisation will occur in theatre at the time of surgery either by a member of the clinical research team or a member of the surgical team. If not possible, randomisation can occur on days prior to the surgery to allow for time to coordinate the drug supplies and inform the operative and anaesthetic team. If consent has occurred more than a day before randomisation, the local research team will verbally check the patient still agrees to participation before proceeding with randomisation.

Full details of the randomisation are available in SPAARK_RBP_v3.0_19Apr2018, stored in the confidential statistical section of the TMF.

The patient will be blinded to the treatment allocation. Unblinding will be performed on an individual basis as clinical need dictates. Surgeons who administer the medication cannot be blinded to treatment allocation; however, blinding of surgeons and outcome assessors is not necessary as the two co-primary outcomes are patient reported measures.

3.3 Sample Size

The trial has been powered for two primary endpoints, QoR-40 at 72 hours and cumulative pain score from 6-72 hours, with adjustment for multiplicity using the Bonferroni method [16]. The trial will be assessed as providing evidence of a difference if either of the two primary endpoints is statistically and clinically significant.

The study requires 240 patients per treatment arm in order to be 90% powered to detect a 5 point difference in global QoR-40 score between groups at a significance level of 0.025 (2-sided, adjusted for multiplicity) assuming the standard deviation is 15.5 [17]. To allow for 4% loss to follow-up this has been inflated to 500 patients (250 per treatment arm).

The study also requires a minimum of 225 patients per treatment arm in order to be 90% powered to detect a standardised difference of 33% between groups in cumulative pain score calculated as area under the curve from 6 to 72 hours post-surgery at a significance level of 0.025 (2-sided, adjusted for multiplicity). Inflating the sample size to 500 patients (250 per treatment arm) will allow for 10% loss to follow up on this variable.

Therefore a total of 500 patients will be randomised (250 per treatment arm).

Checks of the sample size calculations are detailed in the documents PAIN_SS_mar2016.rtf and QoR40_SS_mar2016 both stored in the confidential statistical section of the TMF.

3.4 Statistical Interim Analysis, Data Review and Stopping guidelines

The Data and Safety Monitoring Committee (DSMC) is a group of independent experts external to the trial who will assess the progress, conduct, participant safety and, if required, critical endpoints. The DSMC follows the charter as described in the document SPAARK_DSMC_Charter_v1.0_18Jan2017 stored in the TMF. The DSMC will review accruing data and summaries of the data presented by treatment group. They will assess the screening algorithm against the eligibility criteria. They will also consider emerging evidence from other related trials or research and review related SAEs that have been reported. Full details of the interim analyses planned are available in the Interim Statistical Analysis Plan, SPAARK_Interim Statistical Analysis Plan_V2.0_19Jul2018 stored in the confidential statistical section of the TMF. Formal comparative interim analyses of the co-primary outcomes are not planned during the trial. The DSMC may advise the chair of the TSC at any time if, in their view, the trial should be stopped for ethical reasons, including concerns about participant safety. DSMC meetings will be held at least annually during the recruitment phase of the study.

3.5 Timing of Final Analysis

The final analysis of all primary and secondary endpoints will be conducted together when all recruited patients have completed all follow-up.

3.6 Blinded analysis

A blinded analysis of the data (not separated by treatment group) will be undertaken prior to the final data lock in order to look into the distribution of variables, missing data distributions, outliers, and to finalise the per protocol population.

3.7 Statistical Analysis Outline

The primary objective of improved patient recovering will be assessed by analysing the two primary endpoints: global QoR-40 scores at 72 hours and cumulative pain score from 6 to 72 hours following surgery. The QoR-40 is a global measure of quality of recovery. It incorporates five dimensions of health: patient support, comfort, emotions, physical independence, and pain; each item is graded on a five-point Likert scale. QoR-40 scores range from 40 (extremely poor quality of recovery) to 200 (excellent quality of recovery) [18]. The null hypothesis that there is no difference between the treatment arms will be rejected if either of these outcomes are statistically different at the 2.5% significance level. The trial will be considered to have a positive result if either of the dual primary outcomes returns a positive result.

Pain scores will be assessed daily in the evening at approximately 6, 24, 48, and 72 hours post-surgery, using a 0-10 VAS and cumulative pain scores will be calculated as area under the curve.

Both of the primary outcomes will be analysed using multivariate linear regression adjusting for stratification factors and other important prognostic variables, including baseline scores in the case of the QoR-40. The adjusted mean difference between the two groups together with 97.5% confidence intervals will be reported for each of the primary outcomes. If either of the primary outcomes is not normally distributed, transformation to normality will be the first approach. If this is not possible, then non-parametric techniques without adjustment will be used.

As secondary analyses both pain and QoR-40 measured at 6, 24, 48 and 72 hours will be assessed using longitudinal methods to take account of the multiple-time points and the correlation between them. Multi-level, multivariate linear regression modelling with adjustment for the same factors as the primary analysis will be used. Other continuous variables will be analysed using similar methodology. For binary variables the number and percentage of patients in each category will be reported for each treatment group and overall. Chi-squared tests will be utilised for comparing the treatments, with additional analyses being undertaken using multi-variate logistic regression with adjustment for stratification and other important prognostic factors if sufficient events have been observed.

Reporting of the results will be based on the CONSORT statement.

It is anticipated that all statistical analysis will be undertaken using Stata (StataCorp LP, www.stata.com) or other well-validated statistical packages.

4. STATISTICAL PRINCIPLES

4.1 Statistical Significance and Multiple Testing

A significance level of 0.025 will be used and 97.5% confidence intervals will be presented for each of the two primary outcomes. The trial will be considered to have a positive result if either of the dual primary outcomes returns a positive result at this significance level.

All secondary analyses will be considered as supporting the primary analysis and will be analysed using a significance level of 0.05 with 95% confidence intervals.

Interim analyses of primary and secondary endpoints will not be carried out unless requested by the DSMC. In this case, p-values of 0.001 will be used for significance.

4.2 Definition of Analysis Populations

Populations for analysis are defined as follows:

Intention to treat (ITT): all participants randomised in their randomised groups.

Per protocol (PP): participants who received the allocated intervention will be analysed according to the treatment they actually received. Participants will be excluded from the PP population if:

- They did not receive the treatment to which they were randomised
- They did not provide sufficient follow-up data for analysis
- They did not satisfy the eligibility criteria for the study

The definition of the PP analysis population will be finalised during a blinded analysis of the data (not separated by treatment group) prior to the final data lock.

5. TRIAL POPULATION AND DESCRIPTIVE ANALYSES

5.1 Representativeness of Study Sample and Patient Throughput

The flow of participants through the trial will be summarised as outlined in Figure 1. This will include the number of individuals screened, eligible, randomised to each group, receiving allocated treatment and included in the primary analysis as suggested in the CONSORT guidelines. Reasons for ineligibility, loss to follow-up and exclusion from the primary analysis will be summarised, as will the number of patients who withdraw before each analysis time point.

Figure 1: CONSORT flow diagram for participants in trial.

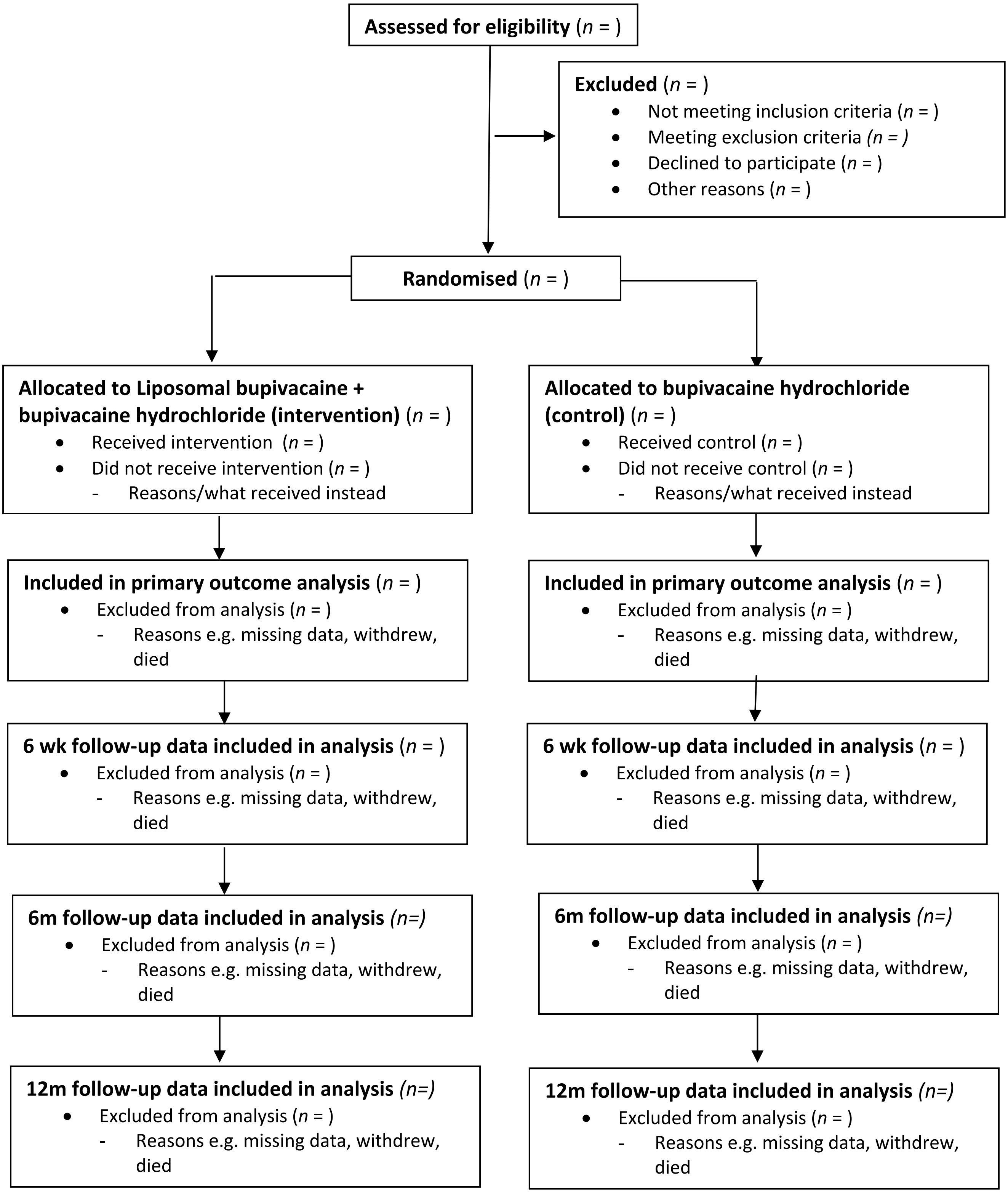

5.2 Withdrawal from treatment and/or follow-up

The numbers (and percentages) of withdrawals by each time point will be reported by treatment group along with reasons for these withdrawals. These will be summarised as outlined in Table 2. Any differential losses will be investigated. Any deaths (and their causes) will be reported separately.

Table 2: Details of withdrawals from follow-up (and reasons) split by treatment group

**Questionnaire**	**Intervention**		**Control**	
	**n**	**%**	**n**	**%**
**Withdrawals (before surgery)**	X	X	X	X
Reason 1	X	X	X	X
Reason 2	X	X	X	X
**…**	X	X	X	X
**Withdrawals (day 0)**	X	X	X	X
Reason 1	X	X	X	X
Reason 2	X	X	X	X
…	X	X	X	X
**Withdrawals (day 1)**	X	X	X	X
Reason 1	X	X	X	X
Reason 2	X	X	X	X
…	X	X	X	X
Repeat for subsequent time points				

5.3 Baseline Comparability of Randomised Groups

The baseline comparability of the two treatment groups (liposomal bupivacaine + bupivacaine hydrochloride versus bupivacaine hydrochloride alone) will be considered. The treatment groups will be compared in terms of stratification factors (see Table 3), baseline characteristics (see Table 4), and primary and secondary outcome measures at baseline (see Table 5). Numbers (with percentages) for binary and categorical variables and the number of available observations with means (and standard deviations), medians (with inter-quartile ranges) and range for continuous variables will be reported. These will be reported for each treatment group and overall. There will be no tests of statistical significance nor confidence intervals for differences between randomised groups on any baseline variable.

Table 3: Stratification factors at baseline – split by treatment group and overall

**Stratification factor**	**Intervention**		**Control**		**Total**	
	**n**	**%**	**n**	**%**	**n**	**%**
**Centre**						
Site 1	X	X	X	X	X	X
Site 2	X	X	X	X	X	X
Site 3	X	X	X	X	X	X
…	X	X	X	X	X	X
**Type of surgery**						
TKR	X	X	X	X	X	X
UKR	X	X	X	X	X	X

Table 4: Baseline characteristics of participants – split by treatment group and overall

	Intervention				Control				Total			
	n	Mean (SD)	Median (IQR)	Range	n	Mean (SD)	Median (IQR)	Range	n	Mean (SD)	Median (IQR)	range
**Age**	X	X (X)	X (X,X)	(X,X)	X	X (X)	X (X,X)	(X,X)	X	X (X)	X (X,X)	(X,X)
**BMI**	X	X (X)	X (X,X)	(X,X)	X	X (X)	X (X,X)	(X,X)	X	X (X)	X (X,X)	(X,X)
	n		%		n		%		n		%	
**Gender**												
Male	X		X		X		X		X		X	
Female	X		X		X		X		X		X	
**Knee**												
Left	X		X		X		X		X		X	
Right	X		X		X		X		X		X	

Table 5: Primary and secondary outcome measures at baseline – split by treatment group and overall

	Intervention	Control	Total
	**N, mean (SD), median (IQR), range**	**N, mean (SD), median (IQR), range**	**N, mean (SD), median (IQR), range**
**QoR 40**	X, X (X), X (X, X), (X, X)	X, X (X), X (X, X), (X, X)	X, X (X), X (X, X), (X, X)
**Pain VAS (0-10)**	X, X (X), X (X, X), (X, X)	X, X (X), X (X, X), (X, X)	X, X (X), X (X, X), (X, X)
**EQ-5D utility**	X, X (X), X (X, X), (X, X)	X, X (X), X (X, X), (X, X)	X, X (X), X (X, X), (X, X)
**EQ-5D VAS**	X, X (X), X (X, X), (X, X)	X, X (X), X (X, X), (X, X)	X, X (X), X (X, X), (X, X)
**OKS**	X, X (X), X (X, X), (X, X)	X, X (X), X (X, X), (X, X)	X, X (X), X (X, X), (X, X)
**AKSS**	X, X (X), X (X, X), (X, X)	X, X (X), X (X, X), (X, X)	X, X (X), X (X, X), (X, X)

5.4 Unblinding

Participants will be blinded to their treatment allocation. Blinding of surgeons, who administer the medication, and outcome assessors will not be necessary since the co-primary outcomes are patient-reported. Unblinding of patients will be performed on an individual basis as clinical need dictates. All cases of treatment unblinding will be summarised by treatment group (numbers and percentages) together with reasons.

5.5 Description of Compliance with Intervention

The trial and control drugs are administered as a single intra-operative dose via peri-articular infiltration. Amount of drug administered will be recorded. As such patient compliance is not relevant to these drugs. If, for any reason, the randomised intervention is not delivered, this will be recorded along with the reason for not receiving the randomised treatment. The instances will be summarised by treatment group as outlined in Table 6. Any differences between the two treatment groups will be explored. In addition, the number of participants experiencing complications during surgery and the total number of complications during surgery will be summarised by treatment group (Table 7), and any differences will be investigated. The types of complications as well as their Clavien-Dindo classification will be summarised.

Summaries will also be provided on time from randomisation to surgery, time in theatre, type of surgery performed and whether this matched the type of surgery planned at randomisation, number of anaesthetists, surgeons and nursing staff present and their grades, ASA grade and anaesthetic used (see Table 7).

Table 6: Details of interventions received split by randomised treatment group

	Intervention		Control	
	n	%	n	%
Received allocated intervention as planned [a]	X	X	X	X
Didn't receive allocated intervention as planned	X	X	X	X
Did not have surgery	X	X	X	X
Reason(s) (repeat as needed)	X	X	X	X
Received alternate trial treatment	X	X	X	X
Reason(s) (repeat as needed)	X	X	X	X
Received a non-trial treatment [b]	X	X	X	X
Reasons(s) (repeat as need)	X	X	X	X
Did not receive all syringes as planned [c]	X	X	NA	NA

[a] *Participants are classed as having received their allocated intervention if for those randomised to Liposomal bupivacaine + bupivacaine hydrochloride all six syringes of Liposomal bupivacaine were administered as planned, and those who were randomised to Bupivacaine hydrochloride only received this*

[b] *Details of non-trial treatments will also be provided*

[c] *Further details regarding this will be provided in Table 8*

Table 7: Details of surgery and complications experienced during surgery summarised by treatment group

	Intervention	Control
Time from randomisation to surgery (days; median, IQR, range)		
Time in theatre (minutes; median, IQR, range)		
TKR performed (n, %)		
Type of surgery different from planned at randomisation (n, %)		
Number of anaesthetists present (median, IQR, range)*		
Number of surgeons present (median, IQR, range)*		
Number of nursing staff present (median, IQR, range)*		
ASA Grade (n, %)		
Grade I		
Grade II		
Grade III		
Anaesthetic used (n, %)		
General		
Neuraxial – spinal		
Neuraxial – epidural		
Block – femoral		
Block – sciatic		
Block – adductor canal		
Block – lumbar plexus		
Patients with complications during surgery (n, %)		
Total no. of complications during surgery (n, %)		

**Further break down by grade will be provided if appropriate.*

The process for administering the IMP is more complex than the control. Compliance with this procedure will be summarised (Table 8), as will the surgeon perception of the difficulty of this procedure (Table 9).

Table 8: Compliance with the IMP administration procedure for those participants receiving liposomal bupivacaine

	Yes		No	
	n	%	n	%
Syringe 1				
Syringe 2				
Syringe 3				
Syringe 4				
Syringe 5				
Syringe 6				
Injections 14pprox.. 1-1.5ml to intended area				
Tissue visibly expanded with minimal leakage				

Table 9: Difficulty of IMP administration for those participants receiving liposomal bupivacaine

	n	%
Simple		
Moderate		
Difficult		

5.6 Reliability

To ensure consistency, validation checks of the data will be conducted. This will include checking for duplicate records, checking the range of variable values and validating potential outliers by comparing with CRFs and referring back to sites if necessary. Calculations and processes performed by a computer program, including the construction of derived data such as total scores on various outcomes, will be checked by hand calculations (where possible). This check will be conducted for a minimum of 5% of the available data or 20 participants randomly sampled from the dataset. These checks will also confirm whether the data has been imported into the statistical software correctly and will check any merging of different datasets. Clarification will be sought from the trial office in the case of discrepancies.

For each variable, missing value codes will be checked for consistency and the proportion of missing values per variable will be presented. Patterns of missing data will be explored. Where missing data imputation is used, imputed values will also be verified using the validation techniques described above. Sensitivity analyses will be conducted to explore the missing data assumptions used.

6. ANALYSIS

6.1 Outcome Definitions

The ***co-primary outcome measures*** in this trial are the Quality of Recovery 40 score and the cumulative daily pain score both measured at 72 hours post-surgery.

Quality of Recovery (QoR) 40 score – a five dimension validated patient reported peri-operative recovery score that has been widely used across a range of surgeries including knee replacement and assesses overall post-operative recovery [19]. It consists of 40 items each of which is graded on a five-point Likert scale. Total scores are calculated by summing the individual items. QoR-40 scores range from 40 (extremely poor quality of recovery) to 200 (excellent quality of recovery) [18]. The primary time point will be 72 hours post-surgery.

This outcome will also be measured at 6, 24 and 48 hours post-surgery and at 6 weeks, 6 and 12 months post-randomisation.

Cumulative daily pain score – pain at rest will be measured using a visual analogue scale (VAS) ranging from 0 (no pain) to 10 (worst possible pain). Measurements taken as close as possible to 6, 24, 48, and 72 hours post-surgery will be included in the primary outcome assessment. This outcome will also be measured at 6 weeks, 6 and 12 months post-randomisation.

The ***secondary outcome measures*** in this trial are cumulative opiate use, the Oxford Knee Score, the American Knee Society Score, fitness for discharge, and the co-primary outcome measures at additional time points. The health economics outcomes in this trial include summary medication history, EQ-5D-5L, summary healthcare and social services use, and summary employment history.

Cumulative opiate use – all opioids used on evening of surgery (day 0) and days 1, 2, and 3 will be recorded and a running total of cumulative opioid use calculated. A list of acceptable opioids is provided in the trial protocol.

Fitness for discharge – this will be assessed against pre-defined criteria on evening of surgery (day 0) and days 1, 2, and 3 following surgery as per routine clinical care. Patients will be considered fit for discharge when they meet four criteria: (i) ability to mobilise independently; (ii) pain score less than or equal to 3 on a 10 score VAS scale; (iii) ability to straight leg raise; and (iv) ability to bend knee to 90 degrees. It will also be recorded whether patients were discharged, and if they were not discharged when fit for discharge the reasons for this will be collected.

Oxford Knee Score (OKS) – a 12-item patient-reported outcome measure designed to measure pain and function after knee replacement surgery [20]. Each question is scored from 0 (worst outcome) to 4 (best outcome). A total score is obtained by summing across all 12 items to give a single score with a range from 0 (worst outcome) to 48 (best outcome). This outcome will be assessed at 6 weeks, 6 and 12 months post-randomisation.

American Knee Society Score – patient reported outcomes from the AKSS will be collected at baseline, 6 weeks, 6 months and 1 year post-surgery. The AKSS is split into categories where patients record details of satisfaction with treated knee, expectations from their surgery, and ability to perform functional activities. Total scores will be calculated using the scoring manual [21]. The patient satisfaction score composes 5 questions with a maximum score of 40. The patient expectation score composes 3 questions with a maximum score of 15. The functional score is composed of 4 subgroups (walking and standing; standard activities; advanced activities and discretionary activities) with a maximum score of 100. Higher scores indicate better performance. This outcome will be assessed at 6 weeks, 6 and 12 months post-randomisation.

EQ-5D – a validated measure of health-related quality of life consisting of a five dimension health status classification system with 5 response levels (EQ-5D-5L) and a separate visual analogue scale [22, 23]. Responses to the health status classification system will be converted to multi-attribute utility scores, scaled such that 1 is equivalent to perfect health, 0 is equivalent to death, and scores worse than death are possible. The UK value set will be used to generate the EQ-5D-5L index. This outcome will be assessed at 72 hours following surgery, 6 weeks, 6 months and 1 year post-randomisation.

Summary healthcare, social services use and employment history – will be summarised and analysed by the trial health economist. A separate health economics analysis plan will be prepared.

6.2 Analysis Methods

Primary outcomes

Primary analysis

QoR-40 scores at 72 hours post-surgery will be summarised by treatment group using unadjusted means and associated SDs (see Table 10). QoR-40 scores from baseline to 12 months post-randomisation will also be summarised by treatment group using a boxplot.

A mixed effects linear regression model adjusting for type of surgery, baseline QoR-40 scores, age, and gender as fixed effects and recruitment centre as a random effect will be used compare QoR-40 scores at 72 hours post-surgery. The adjusted mean difference between the arms, associated 97.5% confidence interval and associated p-value will be reported (Table 10). The assumption of approximate normality of the residuals will be assessed graphically.

Pain scores from baseline to 12 months post-randomisation will be summarised by treatment group using a boxplot.

A summary statistics approach will be used to calculate the area under the curve (AUC) of cumulative daily pain scores from 6 to 72 hours post-surgery [24]. Specifically, parameters from a repeated measures mixed effects linear regression model will be used to calculate the pain AUC for each treatment group. The model will include repeated measures of the pain scores (level 1) nested within participants (level 2) and adjusted for recruitment centre as a random effect (level 3). Time will be treated as categorical and a treatment by time interaction will be included. The model will be adjusted for type of surgery, baseline pain score, age, gender, and the use of pre-operative opiate pain medication as fixed effects. The AUC for each treatment group and associated SD will be calculated following the formula lay out by Bell et al. [24] and using the lincom command in Stata. Time will be treated linearly and AUCs will be calculated for the median values of continuous covariates and most common value of categorical covariates. These results will be presented as shown in Table 10. Similar methods will be used to calculate the difference between the two groups which will be compared using a t-test. The adjusted difference (97.5% CI) and associated p-value will be presented (Table 10). The assumption of approximate normality of the residuals of the mixed effects model will be assessed graphically.

If, in either case, approximate normality of the residuals is not appropriate, the first approach will be to consider a transformation of the data. If approximate normality cannot be achieved by transformation, the data will be analysed using a non-parametric equivalent with no adjustment. In the case of the AUC analysis, this may result in a return to the summary measures approach (see below). Medians and inter-quartile ranges will be reported for each treatment group.

Note that the above described approach differs from that described in the trial protocol, whereby individual AUCs were calculated separately for each participant, and then analysed using a multivariate regression model, also referred to as summary measures approach. The summary statistics approach described here has been shown to be superior to the summary measures approach in terms of precision, bias and handling of missing observations [24]. The original analysis approach will be performed as a sensitivity analysis as part of the secondary analyses (see below).

These analyses will be conducted for the ITT population as defined in Section 4.2.

Table 10: Analysis of co-primary outcomes at primary endpoints (ITT population)

	Intervention		Control		Adjusted diff	p-value
	**n**	**mean (SD)**	**n**	**mean (SD)**	**(97.5% CI)**	
**QoR 40 (72 hours)**	X	X (X)	X	X (X,X)	X (X)	X
**Pain (AUC 6-72 hours)**	X	X (X)	X	X (X,X)	X (X)	X

Secondary analysis

Repeated measures of each of the primary outcomes (at 6, 24, 48, 72 hours, 6 weeks, 6 months and 1 year) will be modelled using multi-level mixed-effects linear regression models. The assumption of approximate normality of the residuals will be assessed graphically. These models will use repeated measures (level 1) nested within participants (level 2) and will include a random effect to account for heterogeneity in response due to recruitment centre (level 3). The models will also include fixed effects for type of surgery (TKR versus UKR), baseline values, age, gender and, in the case of the pain scores, use of pre-operative opiate pain medication. Time will be treated as categorical and interactions between treatment and time will be included in the model to allow for treatment effects to vary over the follow-up. The adjusted difference between treatment groups (and associated 97.5% confidence interval) will be reported (see Table 11). This analysis will be conducted for the ITT population (see Section 4.2).

If, in either case, approximate normality of the residuals is not appropriate, the first approach will be to consider a transformation of the data. If approximate normality cannot be achieved by transformation, the data will be analysed separately at each time point using a non-parametric equivalent with no adjustment. Medians and inter-quartile ranges will be reported for each treatment group.

In addition, a summary measures approach to cumulative pain will also be used as a supporting analysis for this outcome. For each participant, cumulative pain score from 6 to 72 hours post-surgery will be calculated as the area under the curve. Participants with missing pain scores at either 6 or 72 hours will be excluded from this analysis. The potential impact of missing data will be investigated separately. Pain score AUCs will be summarised by treatment group using unadjusted means and associated SDs (Table 11). A multivariate linear regression will be used to compare the AUCs between the treatment groups, adjusted as described previously. The adjusted difference (97.5% CI) and associated p-value will be presented (Table 11).

Table 11: Secondary analysis of primary outcomes (ITT population)

		Intervention		Control		Adjusted diff (97.5% CI)	p-value
		n	mean (SD)	n	mean (SD)		
**QoR-40**	**6 hours**	X	X (X)	X	X (X)	X (X, X)	X
	**24 hours**	X	X (X)	X	X (X)	X (X, X)	X
	**48 hours**	X	X (X)	X	X (X)	X (X, X)	X
	**72 hours**	X	X (X)	X	X (X)	X (X, X)	X
	**6 weeks**	X	X (X)	X	X (X)	X (X, X)	X
	**6 months**	X	X (X)	X	X (X)	X (X, X)	X
	**1 year**	X	X (X)	X	X (X)	X (X, X)	X
**Pain VAS**	**6 hours**	X	X (X)	X	X (X)	X (X, X)	X
	**24 hours**	X	X (X)	X	X (X)	X (X, X)	X
	**48 hours**	X	X (X)	X	X (X)	X (X, X)	X
	**72 hours**	X	X (X)	X	X (X)	X (X, X)	X
	**6 weeks**	X	X (X)	X	X (X)	X (X, X)	X
	**6 months**	X	X (X)	X	X (X)	X (X, X)	X
	**1 year**	X	X (X)	X	X (X)	X (X, X)	X
**Pain VAS**	**Summary measures AUC**	X	X (X)	X	X (X)	X (X, X)	X

Secondary outcomes

Continuous outcomes (OKS and AKSS) will be summarised over time (from baseline to 12 months post-randomisation) by treatment groups using boxplots and will be analysed using similar methods to those outlined for the secondary analysis of the primary outcomes. Multi-level, mixed-effects linear regression

models analogous to those described previously will be used. The assumption of approximate normality of the residuals will be assessed graphically. The adjusted difference between treatment groups (and associated 95% confidence interval) will be reported (see Table 12).

Cumulative opiate use will be approximately continuous; however, it is unlikely to be normally distributed. As described above the possibility of transformation to achieve approximate normality will be considered. If normality can be achieved linear regression will be used as previously described. If this cannot be achieved a non-parametric analysis with no adjustment will be used. The results will be summarised as outlined in Table 12.

Table 12: Analysis of continuous secondary outcomes

**Scale**		**Intervention**		**Control**		**Adjusted diff (95% CI)**	**p-value**
		**Mean (SD)[1]**	**N**	**Mean (SD)[1]**	**N**		
**OKS**	**6 weeks**	X (X)	X	X (X)	X	X (X,X)	X
	**6 months**	X (X)	X	X (X)	X	X (X,X)	X
	**1 year**	X (X)	X	X (X)	X	X (X,X)	X
**AKSS - Expectations**	**6 weeks**	X (X)	X	X (X)	X	X (X,X)	X
	**6 months**	X (X)	X	X (X)	X	X (X,X)	X
	**1 year**	X (X)	X	X (X)	X	X (X,X)	X
**AKSS - Satisfaction**	**6 weeks**	X (X)	X	X (X)	X	X (X,X)	X
	**6 months**	X (X)	X	X (X)	X	X (X,X)	X
	**1 year**	X (X)	X	X (X)	X	X (X,X)	X
**AKSS - Function**	**6 weeks**	X (X)	X	X (X)	X	X (X,X)	X
	**6 months**	X (X)	X	X (X)	X	X (X,X)	X
	**1 year**	X (X)	X	X (X)	X	X (X,X)	X
**Cumulative opiate use**		X (X)	X	X (X)	X	X (X,X)	X

[1]Or Median (IQR)

Fitness for discharge is a binary variable. The number and percentage of patients fit for discharge at days 0, 1, 2 and 3 following surgery will be summarised by treatment group (see Table 13). Any instances where someone is fit for discharge at one time point and not at some subsequent one will be summarised. The two treatment groups will be compared using a multi-level logistic regression model with repeated measures (level 1) nested within participants (level 2) and adjusted for recruiting centre as a random effect (level 3). The model will also be adjusted for type of knee replacement, age and gender as fixed effects. Time will be included as a categorical covariate and a treatment-by-time interaction will be included. Adjusted ORs and associated 95% confidence intervals will be reported at each time point (see Table 13). Unadjusted ORs will also be calculated.

Fitness for discharge and actual discharge status will also be summarised by treatment group at each time point (see table 14). Reasons for discrepancies will also be summarised. The average lengths of time from surgery to being determined fit for discharge and from being determined fit for discharge to being discharged will be summarised by treatment group using medians and IQRs.

Table 13: Analysis of secondary outcomes (categorical)

		**Intervention**		**Control**		**Difference (95% CI)**	
		**N**	**%**	**N**	**%**	**Unadj.**	**Adj.**
**Fitness for discharge**	**Day 0**	X	X	X	X	X (X,X)	X (X,X)
	**Day 1**	X	X	X	X	X (X,X)	X (X,X)
	**Day 2**	X	X	X	X	X (X,X)	X (X,X)

	**Day 3**	X	X	X	X	X (X,X)	X (X,X)
**Complications**	**N overall**	X	-	X	-	-	-
	**Participants**	X	X	X	X	X (X,X)	X (X,X)

Table 14: Comparison of fitness for discharge and actual discharge status

		**Intervention (n, %)**		**Control (n, %)**	
		**Discharged**	**Not discharged**	**Discharged**	**Not discharged**
**Day 0**	**Fit for discharge**	X (X)	X (X)	X (X)	X (X)
	**Not fit for discharge**	X (X)	X (X)	X (X)	X (X)
**Day 1**	**Fit for discharge**	X (X)	X (X)	X (X)	X (X)
	**Not fit for discharge**	X (X)	X (X)	X (X)	X (X)
**Day 2**	**Fit for discharge**	X (X)	X (X)	X (X)	X (X)
	**Not fit for discharge**	X (X)	X (X)	X (X)	X (X)
**Day 3**	**Fit for discharge**	X (X)	X (X)	X (X)	X (X)
	**Not fit for discharge**	X (X)	X (X)	X (X)	X (X)

The total number of complications experienced will be summarised by treatment group, as well as the number of participants experiencing at least one complication. The number of participants experiencing a complication will be compared using a logistic regression model adjusted as outlined previously (see Table 13). Descriptive data on the types of complications experienced, Clavien-Dindo classification of complications and timing of complications will also be presented.

6.3 Missing Data

For each of the co-primary and secondary outcomes, the number and percentages of individuals in the missing category will be presented by treatment group, as well as reasons for missingness if known. The pattern of missingness will also be explored.

Based on the available data, the possibility of informative missingness, or data being missing not at random cannot be ruled out. Therefore, sensitivity analysis for missing data will examine the effect on the trial conclusions assuming that data are missing not at random, that is that those with missing data may have different outcomes than those with observed follow-up data.

Specifically, the potential effect of informatively missing data on the QoR-40 will be assessed using Stata's *rctmiss* command. Hereby outcomes for all of those with missing outcome data, and separately for those with missing outcome data in the treatment group, will be assumed to be, on average, up to 5 points worse than under a missing at random assumption. 5 points on the QoR-40 were chosen as this was the target difference used in the sample size calculation.

For the cumulative pain AUC analysis, the summary measures approach will be repeated based on the following imputations:

- Missing data at 6, 24, 48 and 72 hours for those in the whole sample known to have been discharged from hospital at the relevant timepoint will be replaced by a pain score of 3, i.e. the cut-off for participants being deemed fit for discharge.
- Missing pain scores at 6, 24, 48 and 72 hours for those in the whole sample not known to have been discharged from hospital by the relevant time point will be imputed with the worst pain score observed across both trial groups at this time point.

The main purpose of the sensitivity analysis for missing data is to examine if the change in the assumptions made about missing data change the trial conclusions.

For individual missing items on a questionnaire, the imputation rules will be as follows:

- QoR-40: if 3 or fewer answers are missing median imputation based on the other questions in the same domain will be used. If more than 3 answers are missing, the total score will be treated as missing.
- OKS: mean imputation based on the response to all other questions will be used for missing items on the OKS if 2 or fewer answers are missing. If more than 2 answers are missing, the total score will be treated as missing.
- AKSS: mean imputation will be applied to missing items in the patient satisfaction and expectations domains. If fewer than 50% of answers are missing, mean imputation based on the response to all other questions in the same domain will be used. If 50% or more of the answers are missing, the total score will be treated as missing. The functional score domain composes of four subgroups, including: walking and standing, standard activities, advanced activities and discretionary activities. No imputation will be undertaken for the walking and standing domain. Mean imputation will be used for standard and advanced activities domains. In the discretionary activities domain where participants are asked to select up to 3 activities and rate how much bother their knee gives them during this activity, if a patient indicates less than three activities, two assumptions will be investigated: (i) assume that where a discretionary activity is missing this doesn't necessarily mean they couldn't do any, therefore mean imputation will be used. If no activities are given, the discretionary activities score will be treated as missing. (ii) assume that where a discretionary activity is missing this is because the participant couldn't do any further discretionary activities, and a score of zero will be given for this activity (worst score). If no activities are given, the discretionary activities score will be treated as zero.

6.4 Sensitivity Analysis

For the co-primary outcomes, a sensitivity analysis will be carried out on a per-protocol basis using the definition of the PP population given in Section 4.2. This will examine the robustness of conclusions to different assumptions about departures from randomised policies. Sensitivity analyses for checking the validity of MI assumptions, if applicable, will be performed as described in Section 6.3.

The forest plots will be undertook to compare the QoR40 among different sites.

6.5 Pre-specified Subgroup Analysis

No formal subgroup analyses are planned.

6.6 Supplementary/ Additional Analyses and Outcomes

No further analyses are planned.

6.7 Harms

A serious adverse event (SAE) is any untoward medical occurrence that results in death, is life-threatening, requires inpatient hospitalisation or prolongation of existing hospitalisation, results in persistent or significant disability/incapacity, consists of a congenital anomaly or birth defect or is otherwise considered medically significant by the investigator.

Any adverse event (AE) occurring within 30 days of surgery will be recorded if there is a reasonable possibility it is related to either: (i) the administration of the investigational medical product (IMP) or the

control drug; or (ii) the knee replacement surgery. Expectedness and causality of AEs and SAEs will also be determined. AEs occurring more than 30 days following surgery will only be recorded if (i) the event is deemed to be serious (meets SAE criteria) and there is a reasonable possibility it is related to the knee replacement surgery; or (ii) there is a patient death.

Details of expected events related to either the administration of the IMP during surgery or the knee replacement surgery itself are provided in the trial protocol. It is noted that reported AEs related to the IMP have been shown to occur at a similar rate as those in the corresponding bupivacaine hydrochloride controls in active comparator studies.

The number of AEs occurring up to 30 days post-surgery will be reported by treatment group. The number of participants experiencing an AE will also be reported by treatment group, and the two groups will be compared. The number of SAEs, SARs, and SUSARs occurring up to 12 months post-randomisation will be reported by treatment group (Table 15). The numbers of participants experiencing an SAE, SAR or SUSAR will also be summarised by treatment group and will be compared between the liposomal bupivacaine + bupivacaine hydrochloride and bupivacaine hydrochloride alone groups by examination of the 95% confidence intervals for the difference in incidence. The analysis will be conducted for the ITT population as defined in Section 7. The number of deaths per arm will also be compared. Descriptive data on the type of AE reported, as well as MedDRA codes for the SAEs will also be presented.

Table 15: Summary of Serious Adverse Events (SAEs) including SARs/SUSARs

	**Intervention**	**Control**	**Total**
Number of SAEs, SARs and SUSARs	X	X	X
SAEs	X	X	X
SARs	X	X	X
SUSARs	X	X	X
Number of participants with SAEs, SARs and SUSARs	X	X	X
Average number of SAEs, SARs and SUSARs per participant (if ≥1)	X	X	X
Number of participants without SAE, SAR or SUSAR	X	X	X
Number of deaths	X	X	X
Number of AEs	X	X	X
Number of participants with AEs	X	X	X

6.8 Health Economics and Cost Effectiveness (where applicable)

Health Economic Analysis: A cost utility analysis will also be performed using quality-adjusted life years (QALYs) as the main health outcome, obtained using the EuroQol EQ5D-5L questionnaire at baseline, 72 hours, 6 weeks, 6 months and 1 year. The perspective of the analysis will be societal and costs will be informed by collecting data on the initial surgery, cost of treatment and analgesic medication, primary/hospital and social care resource use over the first year following surgery, out-of-pocket expenses (e.g. over the counter medications, travel costs to attend consultations, private practitioners, etc.), unpaid care from family members or friends and loss of earnings resulting from knee replacement. Such data will be collected from hospital medical records and using self-completed patient questionnaires at 6 weeks, 6 months and 12 months. Healthcare and social care utilisation will be valued using national databases [25, 26].

The results of the cost utility analysis will be presented using an incremental cost-effectiveness ratio expressed as cost per QALY gained and interpreted as the additional costs/savings associated to the additional QALY benefits of Liposomal bupivacaine compared to bupivacaine hydrochloride.

The statistician is not undertaking this analysis. A separate Health Economic Analysis Plan (HEAP) will be drafted by the trial health economist.

6.9 Meta-analyses (if applicable)

There is no planned meta-analysis in this study.

7. VALIDATION OF THE PRIMARY ANALYSIS

To validate the co-primary outcomes a statistician not involved in the trial will independently repeat the analyses detailed in this SAP, by using different statistical software (if possible). The results will be compared and any discrepancies will be reported in the Statistical report (See OCTRU SOP STATS-005 Statistical Report).

8. SPECIFICATION OF STATISTICAL PACKAGES

All analysis will be carried out using appropriate validated statistical software such as STATA, SAS, SPLUS or R. The relevant package and version number will be recorded in the Statistical report.

9. REFERENCES

1. Gamble, C., et al., *Guidelines for the Content of Statistical Analysis Plans in Clinical Trials.* Jama, 2017. **318**(23): p. 2337-2343.
2. *Top Ten Priorities for Hip and Knee Replacement.* 2014 23 Jul 2014]; Available from: http://www.lindlliance.org.
3. Chan, E.-Y., et al., *Postoperative pain following hospital discharge after knee replacement surgery: a patient survey.* Pain Management, 2013. **3**(3): p. 177-188.
4. Apfelbaum, J.L., et al., *Postoperative pain experience: results from a national survey suggest postoperative pain continues to be undermanaged.* Anesthesia & Analgesia, 2003. **97**(2): p. 534-540.
5. Moskovitz, B., et al., *Analgesic treatment for moderate-to-severe acute pain in the United States: patients' perspectives in the Physicians Partnering Against Pain (P3) survey.* Journal of opioid management, 2011. **7**(4): p. 277-286.
6. Gottschalk, A., et al., *Prospective evaluation of pain and analgesic use following major elective intracranial surgery.* Journal of neurosurgery, 2007. **106**(2): p. 210-216.
7. Savaridas, T., et al., *Reduced medium-term mortality following primary total hip and knee arthroplasty with an enhanced recovery program: a study of 4,500 consecutive procedures.* Acta orthopaedica, 2013. **84**(1): p. 40-43.
8. Kehlet, H. and J.B. Dahl, *The value of "multimodal" or "balanced analgesia" in postoperative pain treatment.* Anesthesia & Analgesia, 1993. **77**(5): p. 1048-1056.
9. American Society of Anesthesiologists Task Force on Acute Pain Management, *Practice guidelines for acute pain management in the perioperative setting: an updated report by the American Society of Anesthesiologists Task Force on Acute Pain Management.* Anesthesiology, 2012. **116**(2): p. 248.
10. Bergese, S.D., et al., *Efficacy profile of liposome bupivacaine, a novel formulation of bupivacaine for postsurgical analgesia.* Journal of pain research, 2012. **5**: p. 107-16.
11. Xu, C.-P., et al., *Efficacy and safety of single-dose local infiltration of analgesia in total knee arthroplasty: a meta-analysis of randomized controlled trials.* The Knee, 2014. **21**(3): p. 636-646.
12. Barrington, J., *Texas Center for Joint Replacement*. 2014, Plano, Texas.
13. Berend, K., *Joint Implant Surgeons Inc*. 2014, New Albany, Ohio.
14. Barrington, J. and R. Emerson. *Liposomal bupivacaine: the first 1,000 cases in a new era*. in *Podium Presentation AAOS annual meeting*. 2014.
15. Hamilton, T., et al., *Liposomal bupivacaine infiltration at the surgical site for the management of postoperative pain.* Cochrane Database Syst Rev, 2017(2): p. CD011419.
16. Hochberg, Y. and A. Tamhane, *Multiple comparison procedures.* 1987.
17. Poitras, S., P.E. Beaule, and G.F. Dervin, *Validity of A Short-Term Quality of Life Questionnaire in Patients Undergoing Joint Replacement: The Quality of Recovery–40.* J Arthroplasty, 2012. **27**(9): p. 1604-1608. e1.
18. Myles, P.S., et al., *Development and psychometric testing of a quality of recovery score after general anesthesia and surgery in adults.* Anesthesia & Analgesia, 1999. **88**(1): p. 83-90.
19. Herrera, F.J., J. Wong, and F. Chung, *A systematic review of postoperative recovery outcomes measurements after ambulatory surgery.* Anesthesia & Analgesia, 2007. **105**(1): p. 63-69.
20. Dawson, J., et al., *Questionnaire on the perceptions of patients about total knee replacement.* J Bone Joint Surg Br, 1998. **80**(1): p. 63-69.
21. *The 2011 Knee Society Knee Scoring System - LICENCED USER MANUAL.* 25 October 2019]; Available from: http://kneesociety.org/wp-content/uploads/2017/08/2011-KSS-User-Manual_FINAL_12-2012.pdf.
22. Brooks, R. and E. Group, *EuroQol: the current state of play.* Health policy, 1996. **37**(1): p. 53-72.
23. Herdman, M., et al., *Development and preliminary testing of the new five-level version of EQ-5D (EQ-5D-5L).* Quality of life research, 2011. **20**(10): p. 1727-1736.
24. Bell, M.L., M.T. King, and D.L. Fairclough, *Bias in area under the curve for longitudinal clinical trials with missing patient reported outcome data: summary measures versus summary statistics.* SAGE Open, 2014. **4**(2): p. 2158244014534858.
25. Curtis, L., *Unit Costs of Health and Social Care 2014.* Personal Social Services Research Unit, University of Kent, Canterbury, 2014.
26. Health, D.o. *National Schedule of Reference Costs - Year 2012-2013*. 2013 June 2014]; Available from: http://www.gov.uk/givernment/publications/nhs-reference-costs-2012-to-2013.

APPENDIX: GLOSSARY OF ABBREVIATIONS

SAP	Statistical Analysis Plan
DSMC	Data and Safety Monitoring Committee
TSC	Trial Steering Committee
CI	Chief Investigator
AE	Adverse Event
SAE	Serious Adverse Event
SAR	Serious Adverse Reaction
SUSAR	Suspected Unexpected Serious Adverse Reaction
AR	Adverse Reaction
RCT	Randomised Controlled Trial
QoR-40	Quality of Recovery 40 Score
VAS	Visual Analogue Scale
OKS	Oxford Knee Score
AKSS	American Knee Society Score
TMF	Trial Master File
TKR	Total Knee Replacement
UKR	Unilateral Knee Replacement
SD	Standard Deviation
OCTRU	Oxford Clinical Trials Research Unit
SITU	Surgical Interventional Trials Unite
SOP	Standard Operating Procedure
ITT	Intention-to-treat
PP	Per Protocol
MAR	Missing at random
MI	Multiple Imputation
