# Supplementary material for: Efficacy of Liposomal Bupivacaine and Bupivacaine Hydrochloride vs Bupivacaine Hydrochloride Alone as a Periarticular Anesthetic for Patients Undergoing Knee Replacement: A Randomized Clinical Trial
Source: JAMA Surg. 2022 Apr 6;157(6):481–9. doi: 10.1001/jamasurg.2022.0713 (PMC8988023; doi:10.1001/jamasurg.2022.0713)
Supplement: Supplement 2. — eAppendix 1. Participating Centers eAppendix 2. Data and Safety Monitoring Committee eMethods. Health Economic Analysis Plan eTable 1. Unit Costs (UK 2019 £) of Health Care Services eTable 2. Costs for Hospital Admissions Undergoing Knee Replacement eTable 3. Cost of Hospital Admissions During Follow-up (2019 Prices) eTable 4. Unit Costs for Equipment (2019 Prices) eTable 5. Unit Costs (UK 2019 £) of Time Away From Paid Employment eTable 6. Stratification Factors at Baseline–Split by Treatment Group and Overall eTable 7. Baseline Characteristics of Participants–Split by Treatment Group and Overall eTable 8. Primary and Secondary Outcome Measures at Baseline–Split by Treatment Group and Overall eTable 9. Details of Surgery and Complications Experienced During Surgery Summarized by Treatment Group eTable 10. Details of Withdrawals From Follow-up (and Reasons) Split by Treatment Group eTable 11. Details of Interventions Received Split by Randomized Treatment Group eTable 12. Analysis of Coprimary Outcomes at Primary End Points (ITT Population) eTable 13. Analysis of Coprimary Outcomes at Primary End Points (PP Population) eTable 14. Secondary Analysis of Primary Outcomes (ITT Population) eTable 15. Oral Morphine Equivalent Daily and Cumulative Opiate Use by Treatment Group eTable 16. Analysis of OKS and AKSS Secondary Outcome eTable 17. Analysis of Categorical Secondary Outcomes eTable 18. Clavien-Dindo Classification of Surgical and Inpatient Complications by Treatment Arm eTable 19. Summary of Serious Adverse Events (SAEs) Including SARs/SUSARs eTable 20. Sensitivity Analysis for Pain VAS Coprimary Outcome (ITT Population) eTable 21. Missing Data on Resource Use and EQ-5D Utility by Treatment Allocation in Each Follow-up Period eTable 22. EQ-5D-5D Utility Scores, EQ-5D-Visual Analog Scale Score (VAS), and Quality-Adjusted Life-Years (QALYs) by Treatment Allocation (Imputed Data) eTable 23. Details of the Index Procedure (Only Including Participants Who Received the S [file jamasurg-e220713-s002.pdf]

Supplementary Online Content

Hamilton TW, Knight R, Stokes JR, et al; Study of Peri-Articular Anaesthetic for Replacement of the Knee (SPAARK) Study Group. Efficacy of liposomal bupivacaine and bupivacaine hydrochloride vs bupivacaine hydrochloride alone as periarticular anesthetic for patients undergoing knee replacement: a randomized clinical trial. *JAMA Surg.* Published online April 6, 2022. doi:10.1001/jamasurg.2022.0713

eAppendix 1. Participating Centers

eAppendix 2. Data and Safety Monitoring Committee

eMethods. Health Economic Analysis Plan

eTable 1. Unit Costs (UK 2019 £) of Health Care Services

eTable 2. Costs for Hospital Admissions Undergoing Knee Replacement

eTable 3. Cost of Hospital Admissions During Follow-up (2019 Prices)

eTable 4. Unit Costs for Equipment (2019 Prices)

eTable 5. Unit Costs (UK 2019 £) of Time Away From Paid Employment

eTable 6. Stratification Factors at Baseline–Split by Treatment Group and Overall

eTable 7. Baseline Characteristics of Participants–Split by Treatment Group and Overall

eTable 8. Primary and Secondary Outcome Measures at Baseline–Split by Treatment Group and Overall

eTable 9. Details of Surgery and Complications Experienced During Surgery Summarized by Treatment Group

eTable 10. Details of Withdrawals From Follow-up (and Reasons) Split by Treatment Group

eTable 11. Details of Interventions Received Split by Randomized Treatment Group

eTable 12. Analysis of Coprimary Outcomes at Primary End Points (ITT Population)

eTable 13. Analysis of Coprimary Outcomes at Primary End Points (PP Population)

eTable 14. Secondary Analysis of Primary Outcomes (ITT Population)

eTable 15. Oral Morphine Equivalent Daily and Cumulative Opiate Use by Treatment Group

eTable 16. Analysis of OKS and AKSS Secondary Outcome

eTable 17. Analysis of Categorical Secondary Outcomes

eTable 18. Clavien-Dindo Classification of Surgical and Inpatient Complications by Treatment Arm

eTable 19. Summary of Serious Adverse Events (SAEs) Including SARs/SUSARs

© 2022 Hamilton TW et al. *JAMA Surgery*.

eTable 20. Sensitivity Analysis for Pain VAS Coprimary Outcome (ITT Population)

eTable 21. Missing Data on Resource Use and EQ-5D Utility by Treatment Allocation in Each Follow-up Period

eTable 22. EQ-5D-5D Utility Scores, EQ-5D-Visual Analog Scale Score (VAS), and Quality-Adjusted Life-Years (QALYs) by Treatment Allocation (Imputed Data)

eTable 23. Details of the Index Procedure (Only Including Participants Who Received the Surgery)

eTable 24. Summary of Cost (£) Component Over the 1-Year Follow-up (Imputed Data)

eTable 25. Life-Years, Quality-Adjusted Life-Years, Health Care Costs, and Cost-effectiveness for the Base-Case Analysis at 1 Year Following Multiple Imputation

eTable 26. Total NHS and PSS Costs (Including Intervention) in US Dollars

eTable 27. Previous RCT Comparing 266-mg Liposomal Bupivacaine Against Periarticular Infiltration With Local Anesthetic

eFigure 1. Forest Plot Showing the Treatment Effect of the Intervention vs Control Based on the QoR-40 Coprimary Outcome Between Recruitment Sites

eFigure 2. rctmiss Sensitivity Analysis for QoR-40 Coprimary Outcome

eFigure 3. Cost-effective Scatterplot for the Base Case Analysis (Imputed Data and Using NHS and PSS Perspective)

eFigure 4. Cost-effectiveness Acceptability Curve for the NHS and Personal and Social Services Perspective and the Societal Perspective for the Base Case Analysis (Imputed Data)

eReferences

This supplementary material has been provided by the authors to give readers additional information about their work.

© 2022 Hamilton TW et al. *JAMA Surgery*.

eAppendix 1. Participating Centers

1. CI/ PI: Professor Hemant Pandit. Leeds Teaching Hospitals NHS Trust. Chapel Allerton Hospital, Leeds, Chapeltown Rd, Leeds LS7 4SA.

2. PI: Mr Kirti Mohalkar. Royal Orthopaedic Hospital NHS Foundation Trust. The Royal Orthopaedic Hospital, Bristol Road South, Northfield, Birmingham B31 2AP.

3. PI: Mr Simon Jones. Mid Yorkshire NHS Trust Hospitals. Pinderfields Hospital, Rowan House, Aberford Road, Wakefield, West Yorkshire, WF1 4DG.

4. PI: Mr Alexander Anderson. The Rotherham NHS Foundation Trust. Rotherham Hospital, Moorgate Road, Rotherham S60 2UD.

5. PI: Mr Anthony Smith. Robert Jones and Agnes Hunt Orthopaedic NHS Foundation Trust. Robert Jones and Agnes Hunt Orthopaedic Hospital, Gobowen, SY10 7AG.

6. PI: Mr Sushrut Kulkarni. Sherwood Forest Hospitals NHS Foundation Trust. Kingsmill Hospital, Mansfield Road, Sutton in Ashfield, NG17 4JL.

7. PI: Mr David Isaac. Torbay and South Devon NHS Foundation Trust. Torbay Hospital, Lowes Bridge, Torquay, Devon, TQ12 5HN.

8. PI: Mr William Bartlett. Whittington Health NHS Trust. The Whittington Hospital, Magdala Avenue, London, N19 5NF.

9. PI: Mr Rohit Rambani. United Lincolnshire Hospitals NHS Trust. Pilgrim Hospital, Sibsey Road Boston Lincs PE21 9QS.

10. PI: Benedict Lankester. Yeovil District Hospital NHS Foundation Trust. Yeovil District Hospital, Higher Kingston, Yeovil. BA21 4AT.

11. PI: Mr Mark Andrews. Bridlington Hospital. York and Scarborough Teaching Hospitals NHS Foundation Trust. Bridlington Hospital, Bridlington, East Yorkshire, YO16 4QP.

© 2022 Hamilton TW et al. *JAMA Surgery*.

eAppendix 2. Data and Safety Monitoring Committee

TSC Members:

1. Mr Stephen McDonnell (Chair), Cambridge Hospitals University Foundation Trust
   Division of Orthopaedic & Trauma Surgery
   Centre for Musculoskeletal Repair Regeneration & Replacement
   Box 180, Addenbrooke's Hospital
   Cambridge CB2 2QQ

2. Dr Milica Bucknall, Keele University.
   Arthritis Research UK Primary Care Centre
   Research Institute for Primary Care & Health Sciences
   Keele University, Staffordshire, ST5 5BG

3. Dr Sabeena Sharma, Oxford University Hospitals NHS Foundation Trust
   John Radcliffe Hospital, Headley Way
   Headington, Oxford OX3 9DU

4. Mr Simon Newman, Oxford University Hospitals NHS Foundation Trust
   Nuffield Orthopaedic Centre, Windmill Rd
   Oxford OX3 7LD

5. Mrs Rosemary Wyber (Patient Representative)
   Surgical Intervention Trials Unit (SITU)
   Botnar Research Centre
   Nuffield Department of Orthopaedics, Rheumatology & Musculoskeletal Sciences
   University of Oxford
   Oxford, OX3 7LD

6. Mrs Rosalind Clow (Patient Representative)
   Surgical Intervention Trials Unit (SITU)
   Botnar Research Centre
   Nuffield Department of Orthopaedics, Rheumatology & Musculoskeletal Sciences
   University of Oxford
   Oxford, OX3 7LD

© 2022 Hamilton TW et al. *JAMA Surgery*.

DSMC Members:

1. Dr Vass Athanassoglou (Chair), Oxford University Hospitals NHS Foundation Trust
   John Radcliffe Hospital, Headley Way
   Headington Oxford OX3 9DU

2. Dr Rajesh Rout, Sanofi Genzyme, UK
   410 Thames Valley Park Drive, Reading, Berkshire, RG6 1PT

3. Ms Katie Pike, University of Bristol.
   Clinical Trials and Evaluation Unit, School of Clinical Sciences
   University of Bristol, Level 7, Queens Building
   Bristol Royal Infirmary

© 2022 Hamilton TW et al. *JAMA Surgery*.

eMethods. Health Economic Analysis Plan

Here, we describe the methods used in the within-trial cost utility analysis in more detail. We adopted the NHS and personal social services (PSS) perspective in the analysis. Our aim is to provide evidence to UK decision makers concerning the implementation of the intervention in the NHS.

Quality of Life Assessment

Participants were asked to complete the EQ-5D-5L questionnaire at randomisation, 6 weeks post-surgery and at six and twelve months after randomisation, and also at days 0,1,2,3 post-operatively. Responses to EQ-5D-5L questionnaires were converted into utility scores using the cross-walk to the 3-level version (1). QALYs were calculated using the area under the curve approach, which involves estimating the average EQ-5D utility between each follow-up time, and weighting by survival time. Partially completed EQ-5D-5L questionnaires will be considered missing.

Costs Assessment

General

To estimate health care costs, unit cost data were applied to resource use (Appendix 5:Table e1). Cost data were sourced from NHS Reference Costs, English Prescription Cost Analysis, Unit Costs of Health and Social Care (2-4), websites or as self-reported by the participants. All unit costs were inflated, where necessary, to 2018-19 prices using the healthcare and community health services inflation index.

Resource use was obtained from questionnaires at 6 weeks, 6 and 12 months. Here participants were asked to report their use of health and social services, informal care and time-off work, between randomisation and six weeks, six weeks and six months, and six months and twelve months. Data were collected on visits to and from healthcare practitioners (NHS and private), admissions to hospital, medication use, changes to their home and equipment provided or purchased, informal care received, travel costs, social care received, and time away from paid employment and time at work affected by their knee. Each of these components of resource use is described in much greater detail below. Patients' receipt of the allocated intervention was also recorded.

© 2022 Hamilton TW et al. *JAMA Surgery*.

eTable 1. Unit Costs (UK 2019 £) of Health Care Services

Resource use type	Unit cost (£)	Source/details
Theatre time (per minute)	16.49	Table R142X from Scottish cost tables 2019 (https://beta.isdscotland.org/find-publications-and-data/healthcare-resources/finance/scottish-health-service-costs)
Hospital bed day	407	Weighted average cost per excess bed data for elective HRG HN22 (very major knee procedures) from the NHS Reference Cost schedule 2017-18, where the weights reflect national activity levels. Inflated to 2019.
GP – surgery*	39	Cost GP patient contact consultation: PSSRU 2019-20 (chapter 10, page 125). Average consultation length of 9 minutes(5) (5)
GP – home*	90	Cost GP patient contact consultation (including qualification costs and direct care staff costs): PSSRU 2019-20 (chapter 10, page 124). Average consultation length of 9.22 minutes (5); assume average 12 minutes travel time for home visits: PSSRU 2009.
GP – telephone*	21	Cost GP patient contact consultation: PSSRU 2019-20 (chapter 10, page 124). Average consultation length of 5 minutes(5)
Nurse in GP surgery**	6	£42 per hour, PSSRU 2019-20 (chapter 10, page 125). Average consultation length of 9.22 minutes(5)
Physiotherapist	63	NHS Reference Cost schedule 2018-19, tab CHS, service code A08A1
Occupational therapist	83	NHS Reference Cost schedule 2018-19, tab CHS, currency code A06A1
A&E	192	NHS Reference Cost schedule 2018-19, tab AE, weighted average of all attendances to a type 1 A&E unit with the exception of dental care and dead on arrival attendances.
Outpatient visit	127	NHS Reference Cost schedule 2018-19, tab total outpatient attendances, weighted average of all service codes
Social services	45	PSSRU 2019-20 (chapter 11, page 138) (social care worker, adult services). Used cost per hour.
Acupuncture/ Chiropractor/Osteopath	94	Estimated as average of costs for acupuncture, chiropractor and osteopath sessions.<br>Acupuncture: NHS Reference Cost schedule 2018-19, tab OPROC, service code 191 (pain management), currency code AB23Z<br>Chiropractor: assumed to cost the same as physiotherapy session (£63)<br>Osteopath: assumed to cost the same as physiotherapy session (£63)
*including direct care staff costs and including qualification costs<br>**including qualification costs		

© 2022 Hamilton TW et al. *JAMA Surgery*.

Methods for assigning UK-based cost estimates

Intervention and index admission costs

The cost per vial of LB was £241.80 and each participant allocated to the intervention arm received a vial. This cost was provided by the manufacturer. Usual care (UC) arm (i.e. control arm) included use of 100 mg plain bupivacaine (diluted with normal saline) used for peri-articular infiltration. The full cost per vial was applied, even if participants did not receive the protocol stipulated dose because the medication is prepared for the surgery and cannot be re-used if only partially used.

No additional costs for normal saline or plain bupivacaine or use of needles / syringes were taken into consideration as these are minimal, were similar across both the study arms and were assumed to be included in the HRG code. The index hospital stay was converted into a Healthcare Resource Group (HRG) and valued using NHS Reference Costs(6). HRGs are groups of ICD-10 diagnoses and OPCS procedures which use comparable levels of healthcare resources. Based on previous research(6), we used the most common ICD-10 code reported in England for knee replacements: M179 (gonarthrosis, unspecified). We also used the most common OPCS procedure reported in that work that was associated with primary knee replacements: W401 (Primary total prosthetic replacement of knee joint using cement)(6) for total knee replacements, and W581 (Primary resurfacing arthroplasty of joint) and Z845 (Tibiofemoral joint, which the study Chief Investigator confirmed was the most common procedure for unicompartmental knee replacements [UKR] in SPAARK) for all UKRs. Both procedures resulted in the same HRGs (see Appendix 5:Table e2). In England, the large majority of index knee replacements consisted of a single finished consultant episode (FCE) and we assumed the same to apply in this trial. If participants reported post-op complications during their index hospital stay, we coded these as additional secondary ICD-10 diagnosis in the index FCE (finished consultant episode), resulting in differences in the CC scores between patients, which were reflected in the costing. This assumption was also based on the analysis of knee replacements in England(6).

Furthermore, we compared theatre time and length of stay during index admission as well as opioids taken after surgery in the two trial arms. We added cost differences to the HRG-based outlined above. This was done as follows:

- Length of stay: we compared the length of stay of each participant with the trial average for the relevant procedure (total or unicompartmental knee replacement). Differences (in days) relative to the trial average were valued using the cost of an excess bed day;
  Note: the length of stay excluded days in intensive care or high dependency units, which were be costed separately.

- Theatre time: we compared theatre time of each participant with the trial average for the relevant procedure (total or unicompartmental knee replacement). Differences (in minutes) relative to the trial average were valued using the average cost per minute in theatre;

- Opioids: we valued opioids taken during hospital stay and added these to the total hospital stay costs.

© 2022 Hamilton TW et al. *JAMA Surgery*.

eTable 2. Costs for Hospital Admissions Undergoing Knee Replacement

HRG	Currency description	National average unit cost (EL tab) (reference costs 2018/19)	Cost of excess bed day (reference costs 2017/18)	Cost of excess bed day adjusted to 2018/19 costs
HN22D	Very Major Knee Procedures for Non-Trauma with CC Score 2-3	£6,334	£415	£425
HN22E	Very Major Knee Procedures for Non-Trauma with CC Score 0-1	£5,882	£430	£440

- *Unit costs were the NHS Reference Cost schedule 2018-19. Excess bed day costs were derived from the NHS Reference Cost schedule 2017-18 and inflated to 2019, as newer versions no longer include information on excess bed day costs*

© 2022 Hamilton TW et al. *JAMA Surgery*.

GP, community and outpatient visits

Participants were asked to record their attendances with a GP (surgery, home, or telephone), physiotherapist or occupational therapist (NHS or Private), outpatient clinic (NHS or Private), home care worker or social worker (NHS or Private) and other types of healthcare received as free text (these were allocated to existing resource use categories or additional categories where possible). Information on contacts with accidents and emergency departments were also collected. Participants were asked to report visits as a results of something to do with their knee. We costed all these visits using the national databases (see Appendix 5:Table e3).

Hospital admissions

Hospital admissions during the trial follow-up (i.e. admissions subsequent to the index admission) were costed based on the trial's readmission case report form. This was completed by research staff, and included ICD-10 codes for the hospitalisations, or, if these were not available, details on the reasons for admission, date of admission and discharge, time in intensive care and high dependency unit, and procedures performed during the intervention, were given. We used these data as the primary source for costing relevant admissions during the trial follow-up. Inpatient days beyond the trim point were costed as excess bed days. NHS Reference costs were applied (see Appendix 5:Table e3), and all admissions were assumed to have taken place in an NHS setting.

For hospital admissions recorded without sufficient information to attach an HRG, we valued these using weighted averages of admissions in the relevant trial arm by type (day case, emergency and planned admissions).

This analysis was based on hospital admissions as reported by sites. Patient-reported hospital admissions were monitored to ensure no relevant hospital stays had been missed.

© 2022 Hamilton TW et al. *JAMA Surgery*.

eTable 3. Cost of Hospital Admissions During Follow-up (2019 Prices)

HRG	Currency description	Cost of admission (EL)	Cost of excess bed day (costs 2017/18; EL)	Cost of excess bed day (2017/18; EL) adjusted to 2018-19 values	Cost of admission (NEL)	Cost of excess bed day (2017/18; NEL)	Cost of excess bed day adjusted to 2018/19 costs
HD23J	Inflammatory, Spine, Joint or Connective Tissue Disorders, with CC Score 0-2	£1,187	£413	£423	£1,970	£375	£384
HD26G	Musculoskeletal Signs or Symptoms, with CC Score 0-3	£2,065	£569	£582	£1,606	£333	£341
HE81C	Infection or Inflammatory Reaction, due to, Internal Orthopaedic Prosthetic Devices, Implants or Grafts, with CC Score 0-2	£3,953	£288	£295	£4,089	£298	£305
HE82D	Other Complications of, Internal Orthopaedic Prosthetic Devices, Implants or Grafts with CC Score 0-1	£3,005	£404	£413	£2,861	£350	£358
WH07B	Infections or Other Complications of Procedures, with Multiple Interventions, with CC Score 0-1	£5,483	£296	£303	£5,574	£329	£337
WH07G	Infections or Other Complications of Procedures, without Interventions, with CC Score 0-1	£1,606	£370	£379	£2,056	£359	£367

EL: elective admission; NEL: non-elective admission. Unit costs were the NHS Reference Cost schedule 2018-19. Excess bed day costs were derived from the NHS Reference Cost schedule 2017-18 and inflated to 2019, as newer versions no longer include information on excess bed day costs

© 2022 Hamilton TW et al. *JAMA Surgery*.

Medications

Patients were asked to report any medications taken, whether they were purchased or prescribed, and the dosage, duration, and frequency. Data on dosage, duration, and frequency is often missing. Each self-reported medication was categorised according to its chemical name where possible. Using all data from the 2019 Prescription Cost Analysis(4), the most common medication within each chemical name was identified, and the cost per item prescribed extracted (net ingredient cost per item).

Each medication was classified as likely to be one-off prescription or a long term prescription by physicians. For those drugs considered long-term, we identified the typically number of prescriptions per year based on recommended use and standard pack sizes from the British National Formulary(7).

Equipment and home changes

Participants were asked to report details of any changes to their home/equipment purchased or provided following the index surgery as well as their financial contribution to it. Self-reported equipment types were allocated into a category, and costs attached to each category (see Appendix 5:Table e4); the same unit costs was assumed regardless of whether the equipment was purchased or provided. Home changes were valued using the self-reported financial contribution and published estimates, if appropriate or missing.

© 2022 Hamilton TW et al. *JAMA Surgery*.

eTable 4. Unit Costs for Equipment (2019 Prices)

Equipment category	***Cost per item (£)***	***Source***
Arm crutch	20.76	NHS Supply chain
Bath board	16.08	NHS Supply chain
Bath lift	232.80	NHS Supply chain
Bath mat	10.54	www.completecareshop.co.uk
Bath sponge	2.63	NHS Supply chain
Bath step	13.14	NHS Supply chain
Bath stool/chair	21.54	NHS Supply chain
Bed cradle	19.20	NHS Supply chain
Bed raiser	18.98	www.completecareshop.co.uk
Bed wedge	21.84	NHS Supply chain
Chair	214.84	NHS Supply chain
Chair raises	19.98	www.completecareshop.co.uk
Chair riser	496.07	NHS Supply chain
Cold knee wrap	26.54	NHS Supply chain
Commode	27.60	NHS Supply chain
Crutch	9.36	NHS Supply chain
Cushion	24.99	www.completecareshop.co.uk
Frame	17.91	NHS Supply chain
Half step	27.23	www.completecareshop.co.uk
Knee brace	20.14	NHS Supply chain
Knee cage	74.40	NHS Supply chain
Knee massager	26.34	www.completecareshop.co.uk
Leg lifter	6.68	NHS Supply chain
Pedal exerciser	24.98	www.completecareshop.co.uk
Pick up	4.28	NHS Supply chain
Rail	8.82	NHS Supply chain
Rollator	45.60	NHS Supply chain
Mobility scooter	722.14	www.completecareshop.co.uk
Shoe horn	2.56	NHS Supply chain
Stick	8.04	NHS Supply chain
Stool	29.99	NHS Supply chain
Toilet frame	21.60	NHS Supply chain
Trolley	21.82	NHS Supply chain
Toilet seat	16.69	NHS Supply chain
Urine bottle	3.14	www.completecareshop.co.uk
Wheelchair	133.32	www.completecareshop.co.uk
Wiper	3.95	NHS Supply chain

© 2022 Hamilton TW et al. *JAMA Surgery*.

Travel costs

Participants were asked to report costs of travelling to attend healthcare visits related to their knee.

Social services

Participants were asked to report details on use of social services (meals on wheels, home care, personal care assistant (£24 per hour), laundry services (£10.5 per service) and other), the number of weeks for which they used the service, and whether they were still using it. These are referred to as personal and social services (PSS).

Informal care

Participants were asked to report whether they received unpaid care from family or friends, the number of weeks for which any care was received, the number of hours of care provided per week, whether their carer took time away from paid work, and whether they were still receiving it. Minimum hourly wage (£8.21) were used if carer did not take time away from paid work. Mean hourly wage for all employee jobs (£18.03) were used to value the time of carers who took time away from paid work. Where data on weeks or hours per week of care were missing (but care is reported to have been received), mean imputation was used.

Lost productivity costs

Participants were asked to report whether they had to take time off paid employment due to their knee and, if so, how many days, if they lost pay and, if so, how much pay they have lost. Self-reported occupation were translated into a SOC category (https://onsdigital.github.io/dp-classification-tools/standard-occupational-classification/ONS_SOC_occupation_coding_tool.html) and the days off work were costed using mean hourly wages for all employee jobs by SOC category (see Appendix 5:Table e5).

Participants were also asked to report how many days at work were affected by their knee and if so, to rank how much they were affected on a scale of 0 to 10. Participants not in paid employment were asked how many days did their knee affect their ability to carry out usual activities and, if so, to rank how much they were affected on a scale of 0 to 10. The self-reported rank was divided by 10 and multiplied by the number of days reported to ascertain the total days at work of lost productivity. These days were costed using mean hourly wages by SOC for those employed full-time and minimum hourly wage for those unemployed/retired.

© 2022 Hamilton TW et al. *JAMA Surgery*.

eTable 5. Unit Costs (UK 2019 £) of Time Away From Paid Employment

Description	SOC 2010 Code	Unit cost	Source/details
Corporate managers and directors	11	28.97	Mean hourly pay (gross) for all employee jobs
Other managers and proprietors	12	17.40	
Science, research, engineering and technology professionals	21	22.99	
Health professionals	22	22.19	
Teaching and educational professionals	23	23.91	
Business, media and public service professionals	24	23.49	
Science, engineering and technology associate professionals	31	15.56	
Health and social care associate professionals	32	14.27	
Protective service occupations	33	18.01	
Culture, media and sports occupations	34	17.12	
Business and public service associate professionals	35	20.33	
Administrative occupations	41	13.68	
Secretarial and related occupations	42	11.99	
Skilled agricultural and related trades	51	10.89	
Skilled metal, electrical and electronic trades	52	15.13	
Skilled construction and building trades	53	13.86	
Textiles, printing and other skilled trades	54	10.92	
Caring personal service occupations	61	10.59	
Leisure, travel and related personal service occupations	62	11.03	
Sales occupations	71	10.08	
Customer service occupations	72	12.09	
Process, plant and machine operatives	81	12.00	
Transport and mobile machine drivers and operatives	82	12.56	
Elementary trades and related occupations	91	10.54	
Elementary administration and service occupations	92	10.03	

© 2022 Hamilton TW et al. *JAMA Surgery*.

Missing data

We followed best practice methods for addressing missing data in cost-effectiveness studies (8). Missing baseline data were imputed using unconditional mean imputation. Data on receipt of allocated interventions and deaths were considered to be complete, and so no imputation were performed. For components of resource use where participants provided responses to any questions in the resource diary, we imputed missing values as zero.

We used multiple imputation by chained equations to impute missing data on EQ-5D-5L utility scores, and cost components (except costs related to the allocated intervention), at each follow-up time point. Each missing value was imputed as a function of follow-up period, sex, age, recruitment site, and baseline EQ-5D score, updated EQ-5D score and components of costs, and the imputation model was run separately by randomised treatment. We used predictive mean matching to create a total number of 30 imputed datasets (i.e. the proportion of data missing across all time periods times 100). We imputed costs and EQ-5D-5L utility score in each period. In periods where death was observed we adjusted these. For costs, we assumed that they were incurred linearly over time, such that, if an individual died half way into a period, they incurred half the predicted costs.

Within-trial analysis

We reported descriptive statistics (means, SD as a minimum) for resource use, costs, and EQ-5D utilities at each follow-up time point using only complete data. Differences between arms for the EQ-5D-5L utilities were estimated using multi-level mixed effects linear regression models, to allow for multiple follow-ups clustered within participant. The model was adjusted for treatment allocation, an interaction between follow-up time and treatment allocation, recruitment site, and, in the case of EQ-5D, baseline utility score. Clustering by site was accounted for using robust standard errors, using the 'cluster' option in Stata. Other outcomes were analysed using simple regression models or t-tests, as appropriate.

Following multiple imputation, we estimated total costs and QALYs for all participants in the SPAARK study from the date of study recruitment until the earliest of death, from study, or the end of follow-up at 1 year. Our analysis followed intent-to-treat principles wherein healthcare resource use, costs and EQ-5D scores were analysed according to treatment allocation, regardless of the treatment actually received. We did not discount total costs and QALYs as the time horizon of the analysis is 12 months.

On each imputed dataset, we estimated mean costs (by type) and QALYs using separate analysis models, as described above. Estimates derived from each imputed dataset were combined using Rubin's rule to estimate the adjusted mean difference and standard error for each outcome. As a sensitivity analyses, we performed a complete case analysis, including only individuals who provided complete data over the 12 month trial duration.

We estimated the Incremental Cost Effectiveness Ratio (ICER) by dividing the mean cost difference between LB and usual care by the mean QALY difference.

We estimated the joint uncertainty around incremental total costs and QALYs (i.e. the difference between LB and usual care), and in the cost-effectiveness, by bootstrapping 1,000 times from each of the n imputed datasets (creating at least 30,000 bootstraps), running the estimation model on each bootstrapped dataset and extracting the estimated treatment effects. From these bootstrapped results, we calculated the probability that LB was more cost-effective than usual care for different threshold values per QALY gained (9). These were calculated by estimating the proportion of bootstrap replicates with a net monetary benefit (NMB) above 0 for each threshold value, where the NMB was given by the product of the mean difference in QALYs and the threshold value minus the mean difference in costs.

© 2022 Hamilton TW et al. *JAMA Surgery*.

eTable 6. Stratification Factors at Baseline–Split by Treatment Group and Overall

**Factor**	**Intervention (n = 267)**		**Control (n = 266)**		**Total (n = 533)**	
	**n**	**%**	**n**	**%**	**n**	**%**
**Centre**						
Centre 1	129	48.3	129	48.5	258	48.4
Centre 2	17	6.4	14	5.3	31	5.8
Centre 3	5	1.9	5	1.9	10	1.9
Centre 4	30	11.2	33	12.4	63	11.8
Centre 5	15	5.6	16	6.0	31	5.8
Centre 6	3	1.1	3	1.1	6	1.1
Centre 7	32	12.0	33	12.4	65	12.2
Centre 8	2	0.7	1	0.4	3	0.6
Centre 9	10	3.7	10	3.8	20	3.8
Centre 10	17	6.4	16	6.0	33	6.2
Centre 11	7	2.6	6	2.3	13	2.4
**Type of surgery**						
TKR	236	88.4	235	88.3	471	88.4
UKR	31	11.6	31	11.7	62	11.6

© 2022 Hamilton TW et al. *JAMA Surgery*.

eTable 7. Baseline Characteristics of Participants–Split by Treatment Group and Overall

	Intervention (n=267)	Control (n=266)	Total (n=533)
**Age[1]**	68.9 (10.1), (39.4, 91.4), 267	69.0 (9.3), (43.5, 90.5), 266	69.0 (9.7), (39.4,91.4), 533
**BMI[1]**	32.0 (6.4), (9.2, 49.7), 263	31.6 (5.9), (10.0, 53.9), 263	31.8 (6.1), (9.2, 53.9), 526
**Gender[2]**			
Male	116 (43.4)	130 (48.9)	246 (46.2)
Female	151 (56.6)	136 (51.1)	287 (53.8)
**Knee[2]**			
Left	115 (43.1)	114 (42.9)	229 (43.0)
Right	149 (55.8)	151 (56.8)	300 (56.3)
**ASA Grade[2]**			
I	18 (7.0)	17 (6.7)	35 (6.8)
II	187 (72.8)	174 (68.2)	361 (70.5)
III	52 (20.2)	64 (25.1)	116 (22.7)

[1] *Summaries are mean (SD), (range), N*
[2] *Summaries are n (%)*

© 2022 Hamilton TW et al. *JAMA Surgery*.

eTable 8. Primary and Secondary Outcome Measures at Baseline–Split by Treatment Group and Overall

	Intervention				Control				Total			
	**n**	**Mean (SD)**	**Median (IQR)**	**Range**	**n**	**Mean (SD)**	**Median (IQR)**	**Range**	**n**	**Mean (SD)**	**Median (IQR)**	**Range**
**QoR-40**	257	183.3 (12.8)	187.0 (178.0, 192.0)	(104.0, 200.0)	249	184.2 (12.0)	189.0 (180.0, 192.0)	(133.0, 199.0)	506	183.7 (12.4)	188.0 (179.0, 192.0)	(104.0, 200.0)
**Pain VAS (0-10)**	256	5.6 (2.5)	6.0 (4.0, 7.1)	(0.0, 10.0)	252	5.2 (2.6)	5.0 (3.0, 7.0)	(0.0, 10.0)	508	5.4 (2.5)	6.0 (4.0, 7.0)	(0.0, 10.0)
**OKS**	263	17.5 (6.8)	17.0 (13.0, 22.0)	(0.0, 36.0)	254	19.2 (7.4)	19.0 (14.0, 25.0)	(4.0, 40.0)	517	18.4 (7.1)	18.0 (13.0, 23.0)	(0.0, 40.0)
**AKSS**	239	53.6 (20.8)	51.0 (38.0, 68.0)	(12.0, 112.0)	232	58.1 (21.2)	58.5 (42.0, 73.0)	(11.0, 131.0)	471	55.8 (21.1)	55.0 (40.0, 71.0)	(11.0, 131.0)

© 2022 Hamilton TW et al. *JAMA Surgery*.

eTable 9. Details of Surgery and Complications Experienced During Surgery Summarized by Treatment Group

	**Intervention**	**Control**
**Participants with surgery form**	**258[1]**	**256[2]**
**Time from randomisation to surgery (days; n, median, IQR, range)**	258, 0.0, (0.0, 5.0), (0.0, 63.0)	256, 0.0, (0.0, 7.0), (0.0, 90.0)
**Time in theatre (minutes; n, median, IQR, range)**	258, 111.0, (90.0, 129.0), (35.0, 270.0)	256, 116.0, (90.0, 131.0), (45.0, 276.0)
**TKR performed, n (%)**	229 (88.8)	232 (90.6)
Missing	0 (0.0)	0 (0.0)
**Type of surgery different from planned at randomisation, n (%)**	11 (4.3)	9 (3.5)
**Number of anaesthetists present (n, median, IQR, range)**	258, 1.0, (1.0, 1.0), (0.0, 4.0)	256, 1.0, (1.0, 1.0), (1.0, 3.0)
**Number of surgeons present (n, median, IQR, range)**	258, 2.0, (2.0, 2.0), (0.0, 4.0),	256, 2.0, (1.0, 2.0), (0.0, 4.0)
**Number of nursing staff present (n, median, IQR, range)**	258, 3.0, (2.0, 4.0), (0.0, 8.0)	256, 3.0, (2.0, 4.0), (0.0, 7.0)
**ASA Grade, n (%)**		
Grade I	18 (7.0)	17 (6.6)
Grade II	187 (72.5)	174 (68.0)
Grade III	52 (20.2)	64 (25.0)
Missing	1 (0.4)	1 (0.4)
**Anaesthetic used, n (%)**		
General	4 (1.6)	9 (3.5)
Neuraxial - spinal	214 (82.9)	210 (82.0)
Neuraxial - epidural	3 (1.2)	2 (0.8)
Block - femoral	11 (4.3)	11 (4.3)
Block - sciatic	2 (0.8)	0 (0.0)
Block - adductor canal	23 (8.9)	24 (9.4)
Block - lumbar plexus	0 (0.0)	0 (0.0)
Missing	1 (0.4)	0 (0.0)
**Patients with complications during surgery, n (%)**	1 (0.4)	3 (1.2)
**Total no. of complications during surgery, n (%)**	1 (0.4)	3 (1.2)

[1]8 participants withdrew from the intervention arm before surgery; 1 patient had their surgery delayed due to the COVID-19 pandemic

[2]8 participants withdrew from the control arm before surgery; 2 patients had their surgeries delayed due to the COVID-19 pandemic

© 2022 Hamilton TW et al. *JAMA Surgery*.

eTable 10. Details of Withdrawals From Follow-up (and Reasons) Split by Treatment Group

**Questionnaire**	**Intervention (n = 267)**		**Control (n = 266)**	
	**n**	**%**	**n**	**%**
**Total**	**10**	**3.7**	**13**	**4.9**
**Before surgery**	**8**	**3.0**	**8**	**3.0**
Consultant no longer participating in study.	2	0.7	1	0.4
Knee replacement surgery cancelled.	0	0.0	1	0.4
Knee replacement surgery postponed.	0	0.0	1	0.4
Knee replacement surgery postponed. No longer eligible to receive IMP (ASA Grade III & contralateral knee).	1	0.4	0	0.0
No longer eligible to receive IMP (ASA Grade III).	1	0.4	1	0.4
Patient became anxious and left prior to surgery	1	0.4	0	0.0
Patient felt length of recovery would affect work.	1	0.4	0	0.0
Patient relisted as PFJ replacement +/- total knee - no guarantee of TKR.	0	0.0	1	0.4
Personal reason (no further details).	2	0.7	2	0.8
Study closing at site before patient will be seen again in November 2019.	0	0.0	1	0.4
**Day 0**	**0**	**0.0**	**1**	**0.4**
Felt angry at level of post pain. Thought study involvement was to blame.	0	0.0	1	0.4
**Day 1**	**0**	**0.0**	**0**	**0.0**
**Day 2**	**0**	**0.0**	**1**	**0.4**
Personal reason (no further details).	0	0.0	1	0.4
**Day 3**	**2**	**0.7**	**1**	**0.4**
No longer eligible to receive IMP (ASA Grade III).	1	0.4	0	0.0
No longer wants to complete questionnaires. Scrub nurse dropped the IMP at time of surgery.	1	0.4	0	0.0
Struggling to complete questionnaires due to partial blindness.	0	0.0	1	0.4
**6 weeks**	**0**	**0.0**	**1**	**0.4**
Pt moving house - too busy to complete questionnaires. Pt broke ankle and finds it too difficult to answer the questions just about pain in her knee.	0	0.0	1	0.4
**6 months**	**0**	**0.0**	**0**	**0.0**
**12 months**	**0**	**0.0**	**1**	**0.4**
Personal reason (no further details).	0	0.0	1	0.4

eTable 11. Details of Interventions Received Split by Randomized Treatment Group

	Intervention		Control	
	n	%	n	%
**Received allocated intervention as planned***	238	89.1	251	94.4
**Didn't receive allocated intervention as planned**	29	10.9	15	5.6
Withdrawn before surgery	8	3.0	8	3.0
Received alternate trial treatment	7	2.6	0	0.0
Did not receive all syringes as planned	10	3.7	-	-
Did not receive IMP or control	3	1.1	5	1.9
Surgery delayed due to COVID-19	1	0.4	2	0.7

* Participants are classed as having received their allocated intervention if for those randomised to Liposomal bupivacaine + bupivacaine hydrochloride all six syringes of Liposomal bupivacaine were administered as planned, and those who were randomised to Bupivacaine hydrochloride only received this

© 2022 Hamilton TW et al. *JAMA Surgery*.

eTable 12. Analysis of Coprimary Outcomes at Primary End Points (ITT Population)

**Outcome**	**Intervention**		**Control**		**Adjusted diff (97.5% CI)**	**P-value**
	**n**	**Mean (SD)**	**n**	**Mean (SD)**		
**QoR 40 (72 hours)**	234	184.72 (13.93)	219	184.41 (15.31)	0.54 (-2.05, 3.13)	0.643
**Pain (AUC 6-72 hours) ***	226	361.8 (307.7)	218	383.2 (306.3)	-21.5 (-46.8, 3.8)	0.057

* To calculate the AUC, categorical covariates were set as the most commonly occurring group (TKR for surgery type, female for gender, no for preoperative opiate use) and as the median value for continuous covariates (70.1 years for age and 6 for baseline pain VAS scores)

© 2022 Hamilton TW et al. *JAMA Surgery*.

eTable 13. Analysis of Coprimary Outcomes at Primary End Points (PP Population)

**Outcome**	**Intervention**		**Control**		**Adjusted diff (97.5% CI)**	**P-value**
	**n**	**Mean (SD)**	**n**	**Mean (SD)**		
**QoR 40 (72 hours)**	218	184.81 (14.01)	215	184.38 (15.37)	0.52 (-2.14, 3.17)	0.663
**Pain (AUC 6-72 hours)***	211	359.0 (296.2)	213	380.6 (299.6)	-21.6 (-47.4, 4.2)	0.060

* To calculate the AUC, categorical covariates were set as the most commonly occurring group (TKR for surgery type, female for gender, no for preoperative opiate use) and as the median value for continuous covariates (70.2 years for age and 6 for baseline pain VAS scores)

© 2022 Hamilton TW et al. *JAMA Surgery*.

eTable 14. Secondary Analysis of Primary Outcomes (ITT Population)

Outcome	Timepoint	Intervention		Control		Adjusted diff (97.5% CI)	P-value
		n	Mean (SD)	n	Mean (SD)		
**QoR-40**	**6 hours**	236	180.5 (15.2)	234	180.1 (14.8)	0.98 (-2.06, 4.02)	0.471
	**24 hours**	243	176.0 (16.1)	237	177.4 (14.5)	-0.19 (-3.20, 2.82)	0.890
	**48 hours**	239	180.5 (16.3)	232	180.7 (15.0)	0.71 (-2.32, 3.75)	0.599
	**72 hours**	234	184.7 (13.9)	219	184.4 (15.3)	0.41 (-2.68, 3.49)	0.768
	**6 weeks**	202	176.9 (18.5)	207	178.1 (15.9)	-0.51 (-3.73, 2.71)	0.721
	**6 months**	191	182.5 (19.0)	184	181.5 (17.5)	1.27 (-2.06, 4.61)	0.393
	**12 months**	184	182.0 (17.7)	175	183.3 (17.7)	-0.98 (-4.40, 2.43)	0.519
**Pain VAS**	**6 hours**	244	4.1 (3.0)	241	4.7 (3.0)	-0.54 (-1.07, -0.02)	0.021
	**24 hours**	247	5.9 (2.5)	242	6.0 (2.4)	-0.12 (-0.64, 0.41)	0.620
	**48 hours**	245	5.0 (2.4)	234	5.2 (2.4)	-0.36 (-0.88, 0.17)	0.131
	**72 hours**	244	4.3 (2.4)	223	4.5 (2.3)	-0.27 (-0.80, 0.26)	0.256
	**6 weeks**	200	3.9 (2.5)	204	4.1 (2.3)	-0.33 (-0.90, 0.24)	0.198
	**6 months**	190	2.8 (2.6)	193	2.6 (2.4)	0.06 (-0.52, 0.64)	0.820
	**12 months**	187	2.2 (2.4)	179	2.2 (2.4)	-0.13 (-0.73, 0.46)	0.611
**Pain VAS**	**Summary measures AUC (6-72 hours)**	236	333.0 (131.5)	217	345.3 (122.6)	-19.9 (-45.6, 5.8)	0.082

© 2022 Hamilton TW et al. *JAMA Surgery*.

eTable 15. Oral Morphine Equivalent Daily and Cumulative Opiate Use by Treatment Group

		Intervention		Control		Adj diff (95% CI)	p-value
		Summary	N	Summary	N		
**Daily opiate use (mg)**[1]	Day 0	33.1 (29.6), 20 (20, 40), (0, 200)	257	33.6 (28.0), 20 (20, 40), (0, 186)	255	-	-
	Day 1	51.1 (35.7), 41 (30, 70), (0, 198)	256	51.5 (47.3), 40 (22, 70), (0, 530)	252	-	-
	Day 2	30.1 (29.9), 20 (10, 40), (0, 200)	229	31.1 (44.9), 20 (10, 40), (0, 520)	217	-	-
	Day 3	20.1 (29.2), 10 (0, 21), (0, 220)	137	23.8 (52.3), 10 (0, 20), (0, 510)	130	-	-
**Cumulative opiate use from day 0 to 3 (mg)**[2]	Best-case[3]	126.5 (88.9)	233	127.4 (132.6)	226	-3.06 (-22.32, 16.19)	0.76
	Worst-case[4]	135.6 (91.4)	233	139.2 (135.9)	226	-6.83 (-26.09, 12.42)	0.49

[1] *Summaries are mean (SD), median (IQR), (min, max).* Opioids were converted to oral morphine equivalent doses using standardized conversion tables

[2] *Summaries are mean (SD)*

[3] *For participants discharged prior to day 3, it is assumed they took no opioids following discharge*

[4] *For participants discharged prior to day 3, it is assumed they took the maximum daily dose of the opioids prescribed at discharge until discharge*

© 2022 Hamilton TW et al. *JAMA Surgery*.

eTable 16. Analysis of OKS and AKSS Secondary Outcome

Scale	Timepoint	Intervention		Control		Adjusted diff (95% CI)	P-value
		Mean (SD)	n	Mean (SD)	n		
**OKS**	**6 weeks**	25.8 (9.6)	207	26.1 (8.6)	216	0.43 (-1.23, 2.10)	0.610
	**6 months**	34.3 (10.0)	199	34.3 (9.3)	199	0.56 (-1.14, 2.25)	0.518
	**12 months**	36.3 (9.4)	191	36.3 (9.0)	185	0.58 (-1.15, 2.30)	0.513
**AKSS – Expectations**	**6 weeks**	8.5 (3.2)	209	8.3 (2.9)	218	0.18 (-0.41, 0.77)	0.549
	**6 months**	9.2 (3.3)	198	9.0 (3.0)	199	0.20 (-0.40, 0.81)	0.508
	**12 months**	9.5 (3.5)	192	9.7 (3.0)	180	-0.14 (-0.76, 0.48)	0.659
**AKSS – Satisfaction**	**6 weeks**	24.0 (9.1)	209	24.4 (8.1)	217	0.09 (-1.59, 1.78)	0.915
	**6 months**	27.7 (9.8)	199	28.4 (8.7)	200	-0.38 (-2.09, 1.34)	0.666
	**12 months**	29.4 (9.9)	194	30.0 (9.0)	180	-0.13 (-1.89, 1.62)	0.881
**AKSS – Function**	**6 weeks**	57.3 (19.6)	207	60.3 (18.2)	212	-1.45 (-5.23, 2.33)	0.452
	**6 months**	60.3 (22.1)	189	61.0 (22.3)	193	2.38 (-1.52, 6.28)	0.232
	**12 months**	65.5 (22.1)	190	64.9 (22.1)	178	2.92 (-1.05, 6.89)	0.149

© 2022 Hamilton TW et al. *JAMA Surgery*.

eTable 17. Analysis of Categorical Secondary Outcomes

	Timepoint	Intervention		Control		Difference (95% CI)	
		n	%	n	%	Unadj.	Adj.
**Fitness for discharge**	**Day 0**	5	1.9	0	0	0.91 (0.42, 2.00)	0.96 (0.44, 2.11)
	**Day 1**	20	7.5	31	11.7	0.50 (0.21, 1.23)	0.51 (0.20, 1.25)
	**Day 2**	77	28.8	70	26.3	1.01 (0.52, 1.97)	1.04 (0.53, 2.02)
	**Day 3**	66	24.7	64	24.1	0.91 (0.42, 2.00)	0.96 (0.44, 2.11)
**Complications**	**Participants***	22	8.2	25	9.4	0.84 (0.44, 1.61)	0.95 (0.48, 1.86)
	Missing	9	3.4	10	3.8	-	-

*The number of patients not experiencing complications was 236 in the intervention arm and 231 in the control arm

© 2022 Hamilton TW et al. *JAMA Surgery*.

eTable 18. Clavien-Dindo Classification of Surgical and Inpatient Complications by Treatment Arm

Clavien-Dindo Classification	Intervention	Control	Total
Inpatient Complications			
**I**	14	20	34
**II**	10	16	26
**III-a**	3	2	5
**III-b**	1	0	1
**IV-a**	0	0	0
**IV-b**	0	0	0
**V**	0	0	0
Surgical Complications			
**I**	1	2	3
**II**	0	0	0
**III-a**	0	0	0
**III-b**	0	1	1
**IV-a**	0	0	0
**IV-a**	0	0	0
**IV-a**	0	0	0

© 2022 Hamilton TW et al. *JAMA Surgery*.

eTable 19. Summary of Serious Adverse Events (SAEs) Including SARs/SUSARs

	Intervention	Control	Total
Number of SUSARS	0	0	0
Number of SARS	0	0	0
Number of SAEs	30	28	58
Number of participants with SAEs	26	26	52
Average number of SAEs per participants (for those with at least one SAE)	1.2	1.1	1.1
Number of participants without SAE, SAR or SUSAR	241	240	481
Number of deaths	3	0	3
Number of AEs	51	56	107
Number of participants with AEs	43	44	87

© 2022 Hamilton TW et al. *JAMA Surgery*.

eTable 20. Sensitivity Analysis for Pain VAS Coprimary Outcome (ITT Population)

**Outcome**	**Timepoint**	**Intervention**		**Control**		**Adjusted diff (97.5% CI)**	**P-value**
		**n**	**Mean (SD)**	**n**	**Mean (SD)**		
**Pain VAS**	**Summary measures AUC (6-72 hours)**	267	355.3 (146.7)	266	375.5 (137.4)	-26.3 (-53.4, 0.9)	0.030

© 2022 Hamilton TW et al. *JAMA Surgery*.

eTable 21. Missing Data on Resource Use and EQ-5D Utility by Treatment Allocation in Each Follow-up Period

	**Healthcare resource use data**			**EQ-5D-5L utility data**	
**Follow-up time**	**Intervention**	**Control**		**Intervention**	**Control**
Baseline	265 (99%)	262 (98%)		262 (98%)	249 (94%)
Post operation: day 0*	n/a	n/a		246 (92%)	236 (89%)
Post operation: day 1*	n/a	n/a		247 (93%)	240 (90%)
Post operation: day 2*	n/a	n/a		249 (93%)	232 (87%)
Post operation: day 3*	n/a	n/a		244 (91%)	219 (82%)
6 weeks*	210 (81%)	218 (85%)		208 (78%)	217 (82%)
6 months	201 (75%)	200 (75%)		198 (74%)	197 (74%)
1 year	194 (73%)	187 (70%)		191 (72%)	182 (68%)

**Note: only expected for participants who underwent surgery*

Self-reported healthcare resource use is classed as missing if no EQ-5D-5L questions, no resource use, and no personal costs were reported.

© 2022 Hamilton TW et al. *JAMA Surgery*.

eTable 22. EQ-5D-5D Utility Scores, EQ-5D-Visual Analog Scale Score (VAS), and Quality-Adjusted Life-Years (QALYs) by Treatment Allocation (Imputed Data)

	Intervention n=267	Control n=266	Treatment effect	
	**mean (SE)**	**mean (SE)**	**Difference (95% CI)**	**p-value**
**EQ-5D-5L utility[1]**				
**Baseline**	0.431 (0.238)	0.458 (0.231)		
**day of surgery***	0.146 (0.017)	0.144 (0.018)	0.006 (-0.040, 0.052)	0.797
**1 day post surgery***	0.324 (0.018)	0.340 (0.018)	-0.013 (-0.063, 0.038)	0.623
**2 days post surgery***	0.444 (0.016)	0.449 (0.017)	-0.001 (-0.038, 0.035)	0.944
**3 days post surgery***	0.518 (0.015)	0.543 (0.015)	-0.021 (-0.080, 0.037)	0.476
**6 weeks post surgery***	0.636 (0.015)	0.637 (0.013)	0.003 (-0.039, 0.045)	0.885
**6 months**	0.723 (0.015)	0.738 (0.013)	-0.011 (-0.055, 0.033)	0.627
**1 year**	0.754 (0.016)	0.766 (0.014)	-0.007 (-0.052, 0.037)	0.747
**EQ-VAS[1]**				
**Baseline**	70.9 (18.8)	70.2 (19.5)		
**day of surgery***	68.7 (1.2)	68.7 (1.2)	-0.4 (-3.0, 2.2)	0.745
**1 day post surgery***	66.2 (1.3)	67.8 (1.2)	-1.7 (-4.4, 0.9)	0.205
**2 days post surgery***	70.2 (1.2)	71.3 (1.1)	-1.4 (-4.1, 1.2)	0.291
**3 days post surgery***	72.6 (1.2)	73.3 (1.2)	-1.2 (-4.7, 2.4)	0.520
**6 weeks post surgery***	73.7 (1.4)	75.0 (1.2)	-2.0 (-5.0, 1.1)	0.205
**6 months**	77.0 (1.4)	78.5 (1.3)	-2.5 (-6.5, 1.6)	0.232
**1 year**	79.0 (1.5)	79.9 (1.3)	-1.4 (-5.8, 3.0)	0.544
**QALY[2]**				
**Baseline to 6 months[3]**	0.320 (0.093)	0.323 (0.081)	-0.001 (-0.022, 0.020)	0.927
**6 to 12 months**	0.368 (0.108)	0.375 (0.095)	-0.004 (-0.028, 0.020)	0.693
**Baseline to 12 months**	0.689 (0.187)	0.698 (0.164)	-0.005 (-0.048, 0.038)	0.793

[1]Differences between treatment arms are obtained from multilevel mixed-effects models, adjusted for baseline utility, type of surgery performed (total vs. partial knee replacement); robust standard errors were used to account for clustering by site; a time by treatment interaction was included in the model; the follow-up time point was used as a categorical variable.
[2]Differences between treatment arms are obtained from regression models, adjusted for baseline utility, type of surgery performed (total vs. partial knee replacement); robust standard errors were used to account for clustering by site.
[3] The QALY data for the baseline to six months period included EQ-5D-5L utility data collected at days zero, one, two, three and 42 days (6 weeks) post-operatively for participants who received their surgery. This is because these assessments varied in terms of their timing from randomisation.
**data only expected for those who had surgery*
Missing baseline data were mean-imputed.

© 2022 Hamilton TW et al. *JAMA Surgery*.

eTable 23. Details of the Index Procedure (Only Including Participants Who Received the Surgery)

	**Intervention (n=267)**				**Control (n=266)**				**Treatment effect[+]**	
	**n**	**mean (SD)**	**median (IQR)**	**range**	**n**	**mean (SD)**	**median (IQR)**	**range**	**Difference (95% CI)**	**p-value**
**Participants receiving surgery[1]**		258/267 (97%)				256/266 (96%)				
**Time in theatre (minutes)**	258	111.5 (33.0)	111 (90, 129)	35, 270	256	113.9 (32.6)	116 (90, 131)	45, 276	-2.4 (-8.1, 3.3)	0.408
**Length of stay (days)**	258	4.1 (1.8)	4 (3, 5)	1, 15	256	4.1 (2.4)	4 (3, 5)	1, 22	-0.1 (-0.5, 0.3)	0.642
**Cost of surgery and length of stay (£)***	258	5939.0 (956.1)	5808 (5354, 6360)	4112, 11365	256	6012.4 (1188.4)	5800 (5360, 6366)	4145, 14412	-73.4 (-260.2, 113.5)	0.441
**Cost of the LB drug (£)[&]**	258	232.4 (46.8)	242 (242, 242)	0, 242	256	0.0 (0.0)	0 (0, 0)	0, 0	232.4 (226.7, 238.2)	0.000
**Cost for Opioids (£)**	258	3.3 (2.7)	3 (1, 4)	0, 21	256	3.2 (2.9)	2 (1, 4)	0, 28	0.2 (-0.3, 0.7)	0.486
**Total cost of hospital stay (£)**	258	6174.8 (961.7)	6039 (5577, 6591)	4357, 11611	256	6015.6 (1189.6)	5802 (5361, 6373)	4145, 14425	159.2 (-28.2, 346.6)	0.096

[+]treatment effects obtained from t-test
The cost for the hospital stay includes: HRG cost, and adjustment for length of stay, critical care bed days (ICU/HDU), adjustment for theatre time and cost of Liposomal bupivacaine if appropriate.
[1]showing n/N (%)

*including procedure costs, adjustment for length of stay and time in theatre

[&]The cost of the bupivacaine hydrochloride is assumed to be included in the HRG code for the primary procedure cost.

© 2022 Hamilton TW et al. *JAMA Surgery*.

eTable 24. Summary of Cost (£) Component Over the 1-Year Follow-up (Imputed Data)

	Intervention (n=267)	Control (n=266)	Treatment effect	
	**Mean (SE)**	**Mean (SE)**	**Difference (95% CI)**	**p-value**
**Total NHS and PPS costs (over one year)**	6779.8 (112.0)	6757.2 (147.7)	22.4 (-410.0, 454.9)	0.908
Primary hospital stay (excluding intervention & opioid costs)	5738.8 (87.3)	5786.4 (100.2)	-47.6 (-290.1, 194.9)	0.700
Intervention[1]	224.6 (3.8)	0.0 (0.0)	224.6 (211.6, 237.6)	<0.001
Opioids	3.2 (0.2)	3.0 (0.2)	0.2 (-0.1, 0.5)	0.262
All hospital readmissions	89.3 (31.8)	118.7 (51.3)	-29.5 (-146.1, 87.1)	0.577
Primary care (NHS)	658.5 (43.4)	718.6 (44.6)	-60.1 (-193.0, 72.8)	0.310
Equipment (NHS)	12.9 (1.4)	14.2 (1.9)	-1.3 (-6.0, 3.4)	0.502
Prescribed medication for knee	43.8 (4.4)	36.9 (4.4)	7.0 (-6.7, 20.6)	0.269
Personal social services	8.6 (5.1)	79.4 (38.3)	-70.8 (-147.1, 5.5)	0.064
**Total non-NHS costs (over one year)**	1068.1 (195.1)	1012.1 (198.6)	56.0 (-515.4, 627.3)	0.817
Healthcare resource use (private)	58.8 (14.6)	53.7 (14.0)	5.1 (-35.1, 45.2)	0.775
Equipment (private)	121.1 (41.6)	79.4 (37.8)	41.7 (-66.7, 150.2)	0.374
Over the counter medication for knee	10.4 (3.6)	4.5 (1.0)	5.9 (-3.4, 15.1)	0.181
Other medication	76.7 (12.8)	66.8 (9.2)	9.9 (-20.0, 39.7)	0.461
Travel costs	23.3 (3.6)	27.8 (3.4)	-4.5 (-17.7, 8.8)	0.460
Informal care	618.5 (176.4)	648.2 (184.8)	-29.8 (-572.0, 512.4)	0.899
Productivity loss	159.4 (53.6)	131.7 (41.6)	27.7 (-88.1, 143.5)	0.549
**Societal costs (over one year)***	7847.9 (227.5)	7769.3 (256.4)	78.4 (-690.5, 847.3)	0.816

Note: missing baseline values mean imputed
Note: treatment effects obtained from linear regression model adjusted for type of surgery performed (total vs. partial knee replacement) and baseline values (where available); robust standard errors were used to account for clustering by site.
**sum of NHS and non-NHS cost*

© 2022 Hamilton TW et al. *JAMA Surgery*.

eTable 25. Life-Years, Quality-Adjusted Life-Years, Health Care Costs, and Cost-effectiveness for the Base-Case Analysis at 1 Year Following Multiple Imputation

	Intervention Mean (SE)	Control Mean (SE)	Difference (Intervention vs control) Mean difference (95% CI)
**N**	267	266	-
**Life-years[1]**	1.0 (0.0)	1.0 (0.0)	0.0 (0.0, 0.0)
**QALYs[$]**	0.689 (0.187)	0.698 (0.164)	-0.005 (-0.048, 0.038)
**Total NHS & PSS costs (including intervention)***	£6779.8 (112.0)	£6757.2 (147.7)	£22.4 (-410.0, 454.9)
**LB drug costs***	£224.6 (3.8)	£0.0 (0.0)	£224.6 (211.6, 237.6)
**Total non-NHS costs***	£1068.1 (195.1)	£1012.1 (198.6)	£56.0 (-515.4, 627.3)
**Total societal costs***	£7847.9 (227.5)	£7769.3 (256.4)	£78.4 (-690.5, 847.3)
**Incremental cost-effectiveness ratios (ICER)****			
**Total NHS & PSS costs**	-	-	Dominated[+]
**Total societal costs**			Dominated[+]
**Probability of cost-effectiveness at willingness to pay threshold of £20,000 per QALY (NHS & PSS perspective)**	-	-	37%

[1]*All but three participants in the intervention arm who died prior to the one-year follow-up were followed up for one year. Means and SD are provided.*
[$]*Differences derived from linear regression model of each treatment allocation against each outcome adjusted for recruitment site and, for QALYs, baseline utility score.*
[+]*Dominated: Intervention is less effective but more costly than control.*
**Differences derived unadjusted linear regression models.*
Abbreviations: CI –confidence interval; PSS – personal and social services; QALY – quality adjusted life year; SE – standard error

© 2022 Hamilton TW et al. *JAMA Surgery*.

eTable 26. Total NHS and PSS Costs (Including Intervention) in US Dollars

	**Original prices (£)**	**GDP PPPs** 2019 (x1.45)**	**Health PPPs** 2017 (x1.33)**	**Exchange rate** 2019 (x1.28)**
**Intervention, mean (SE)**	£6779.8 (112.0)	$9830.7 (162.4)	$9017.1 (149.0)	$8678.1 (143.4)
**Control, mean (SE)**	£6757.2 (147.7)	$9797.9 (214.2)	$8987.1 (196.4)	$8649.2 (189.1)
**Mean difference*, 95% CI**	£22.4 (-410.0, 454.9)	$32.5 (-594.5, 659.6)	$29.8 (-545.3, 605.0)	$28.7 (-524.8, 582.3)

**Differences derived unadjusted linear regression models.*
***PPPs and exchange rate obtained from OECD*
Abbreviations: CI –confidence interval; PSS – personal and social services; SE – standard error

© 2022 Hamilton TW et al. *JAMA Surgery*.

eTable 27. Previous RCT Comparing 266-mg Liposomal Bupivacaine Against Periarticular Infiltration With Local Anesthetic

**Author**	**NCT number**	**Intervention/ Control (*n*)**	**Intervention**	**Control**	**Pain Assessment**	**Opioid Assessment**	**Post Operative Routine Opioids**	**Mean cumulative inpatient opioid mg (SD)[1]: Intervention**	**Mean cumulative inpatient opioid mg (SD)[1]: Control**
Bramlett 2012	NCT00485693	25/35	266mg LB	150mg BHCl	No difference	No difference	No	NS	NS
Schroer 2015	Not stated	58/53	266mg LB + 75mg BHCl	150mg BHCl	No difference	No difference	Yes	52 (32)	54 (33)
Jain 2016	Not stated	63/62	266mg LB	75mg BHCl	No difference	No difference	Yes	NS	NS
Schwarzkopf 2016	Not stated	20/18	266mg LB + 100mg BHCl	250mg RHCl	No difference	No difference	Yes	NS	NS
Collis 2016	Not stated	54/51	266mg LB	250mg RHCl	No difference	No difference	Yes	NS	NS
Snyder 2016	NCT02299349	35/35	266mg LB	400mg RHCl	Lower pain VAS with LB (PACU, d1, d2)	Lower mean opioid with LB (PACU, d3)	Not stated	NS	NS
Barrington 2017	Not stated	40/38	266mg LB + 125mg BHCl	250mg RHCl	Lower pain VAS with LB (6h, 12h)	No difference	No	71 (93)	75 (85)
DeClaire 2017	NCT02808728	47/49	266mg LB +BHCl	RHCl	No difference	No difference	Not stated	98 (43)	90 (59)
Amundson 2017	NCT02223364	55/55	266mg LB + 125mg BHCl	200mg - 400mg Ropivacaine	No difference	No difference	Yes	NS	NS
Alijanipour 2017	NCT02060591	87/75	266mg LD	50mg BHCl	No difference	No difference	No	110(50-185)[2]	90(45-180)[2]
clinicaltrials.gov 2017	NCT02682498	18/20	266mg LB	250mg RHCl	No difference	No difference	Not stated	NS	NS
Mont 2018	NCT02713490	70/69	266mg LB + 100mg BHCl	100mmg BHCl	Lower pain VAS AUC with LB (12 to 48)	Lower cumulative opioid with LB (0 to 48)	No	21 (9)[3]	94 (39)[3]
Suarez 2018	Not stated	52/52	266mg LB + 75mg BHCl	75mg BHCl	No difference	Higher mean opioid with LB (d1)	No	NS	NS

© 2022 Hamilton TW et al. *JAMA Surgery*.

Schumer 2018	Not stated	67/64	266mg LB + BHCl	BHCl	No difference	No difference	Yes	NS	NS
Zlotnicki 2018	Not stated	38/40	266mg LB	100mg BHCl	Lower pain VAS with LB (d1)	No difference	Yes	NS	NS
Hyland 2019	Not stated	30/29	266mg LB	40mg RHCl	No difference	No difference	Yes	275 (121)	305 (143)
clinicaltrials.gov 2019	NCT02543801	59/59	266mg LB	RHCl	No difference	No difference	Not stated	52 (28)[4]	50 (22)[4]

[1]Oral Morphine Equivalent [2]Median (IQR) [3]0 to 72hours [4]0 to 48 hours

© 2022 Hamilton TW et al. *JAMA Surgery*.

eFigure 1. Forest Plot Showing the Treatment Effect of the Intervention vs Control Based on the QoR-40 Coprimary Outcome Between Recruitment Sites

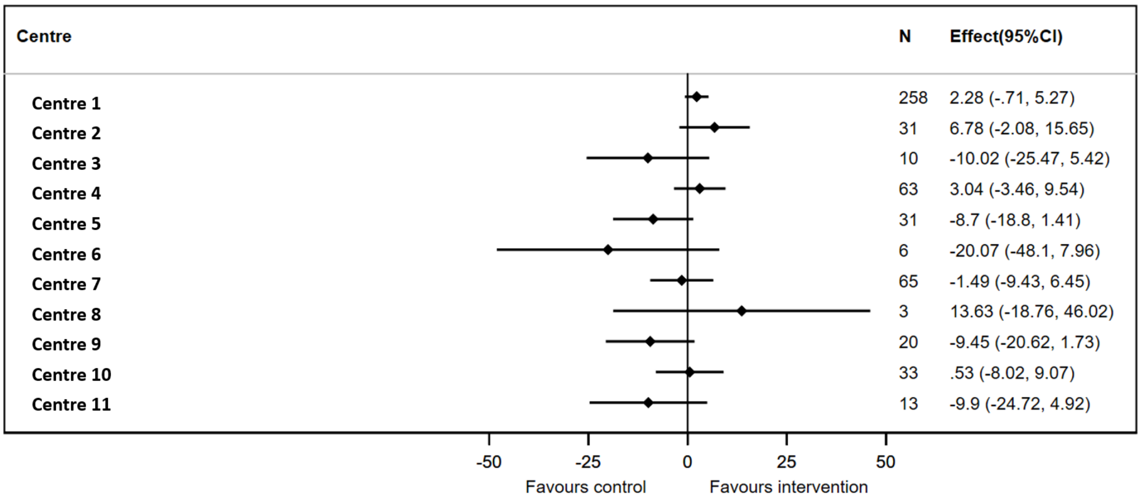

© 2022 Hamilton TW et al. *JAMA Surgery*.

eFigure 2. rctmiss Sensitivity Analysis for QoR-40 Coprimary Outcome

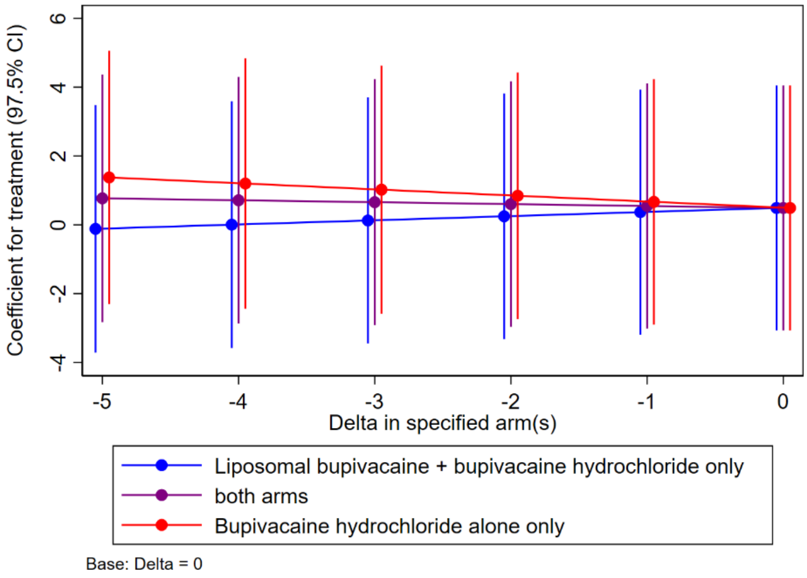

A pattern-mixture model is used to extend a linear regression allowing for a clustering effect for recruitment site. Model is further adjusted for type of surgery (TKR vs UKR), baseline QoR-40 scores, participant age at randomisation and gender.

© 2022 Hamilton TW et al. *JAMA Surgery*.

eFigure 3. Cost-effective Scatterplot for the Base Case Analysis (Imputed Data and Using NHS and PSS Perspective)

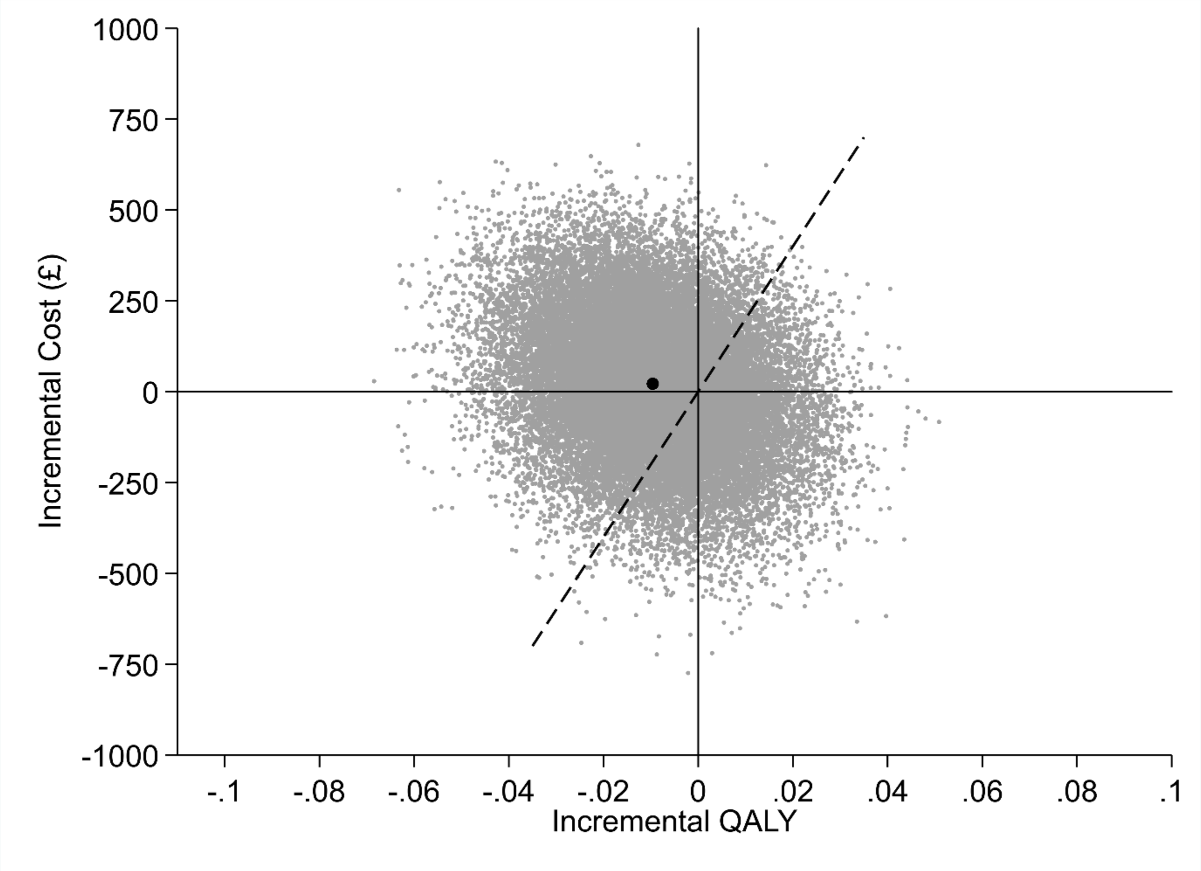

Scatter plot of estimated joint density of incremental costs and QALYs of LB relative to control obtained by bootstrap re-sampling from each of the 30 imputed datasets, running the regression models on each bootstrapped dataset and extracting the estimated incremental costs and QALYs. The dashed line indicates the willingness to pay threshold at £20,000 per QALY gained. Bootstrapped results falling below this line are deemed cost-effective. From the bootstrapped results, we calculated the probability that LB was more cost-effective than control for different threshold values per QALY gained.. *Abbreviations: PSS – personal and social services; QALY – quality adjusted life year*

© 2022 Hamilton TW et al. *JAMA Surgery*.

eFigure 4. Cost-effectiveness Acceptability Curve for the NHS and Personal and Social Services Perspective and the Societal Perspective for the Base Case Analysis (Imputed Data)

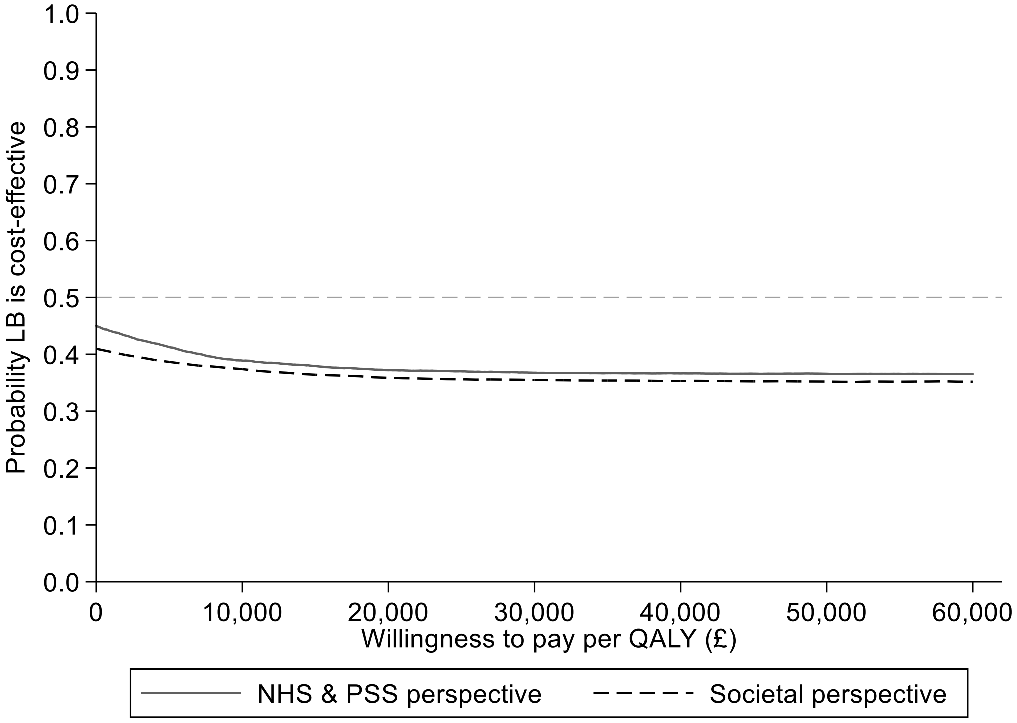

Figure plots the probability (y-axis) that LB intervention is cost-effective compared to control for different willingness to pay thresholds per QALY gain (x-axis). Probability captures the joint uncertainty in incremental costs and QALYs of LB compared to control and was obtained by estimating the proportion of bootstrapped results that were cost-effective for each threshold value. The interpretation is that, given a willingness to pay threshold of £20,000 per QALY gained, the probability that LB is cost-effective compared to control is 0.37 and from a NHS&PSS and a societal perspective, respectively. *Abbreviations: LB - liposomal bupivacaine; PSS – personal and social services; QALY – quality adjusted life year*

© 2022 Hamilton TW et al. *JAMA Surgery*.

eReferences

1. van Hout B, Janssen MF, Feng YS, et al. Interim scoring for the EQ-5D-5L: mapping the EQ-5D-5L to EQ-5D-3L value sets. Value Health. 2012; 15: 708-15.
2. Department of Health. NHS reference costs 2017 to 2018. 2018.
3. Curtis L, Burns A. Unit Costs of Health and Social Care 2018. Kent, UK: Personal Social Services Research Unit, 2018.
4. NHS Business Services Authority. Prescription Cost Analysis (PCA) Data. 2018.
5. Hobbs FDR, Bankhead C, Mukhtar T, et al. Clinical workload in UK primary care: a retrospective analysis of 100 million consultations in England, 2007-14. Lancet. 2016; 387: 2323-30.
6. Leal J, Murphy J, Garriga C, et al. Costs of joint replacement in osteoarthritis: a study using the National Joint Registry and Clinical Practice Research Datalink datasets. Arthritis Care Res (Hoboken). 2020.
7. Joint Fomulary Committee. British National Formulary (online). 2021.
8. Faria R, Gomes M, Epstein D, et al. A guide to handling missing data in cost-effectiveness analysis conducted within randomised controlled trials. Pharmacoeconomics. 2014; 32: 1157-70.
9. Fenwick E, O'Brien BJ, Briggs A. Cost-effectiveness acceptability curves--facts, fallacies and frequently asked questions. Health Econ. 2004; 13: 405-15.

© 2022 Hamilton TW et al. *JAMA Surgery*.
