# Supplementary material for: Efficacy of Liposomal Bupivacaine and Bupivacaine Hydrochloride vs Bupivacaine Hydrochloride Alone as a Periarticular Anesthetic for Patients Undergoing Knee Replacement: A Randomized Clinical Trial
Source: JAMA Surg. 2022 Apr 6;157(6):481–9. doi: 10.1001/jamasurg.2022.0713 (PMC8988023; doi:10.1001/jamasurg.2022.0713)
Supplement: Supplement 3. — Nonauthor Collaborators [file jamasurg-e220713-s003.pdf]

Supplemental Online Content: Nonauthor Collaborators

*Indicates required information. Only first name, last name, and suffix will appear in PubMed.

*Group Name(s): Study of Peri-Articular Anaesthetic for Replacement of the Knee (SPAARK) Study Group							
***First Name and Middle Initial(s)**	***Last Name**	***Suffix (eg, Jr, III)**	Academic Degrees	Institution	Location (city, state/province, country)	Role or Contribution, eg, chair, principal investigator	Group (if more than 1 Group listed in the byline) and/or Subgroup (eg, Steering Committee)
Kirti	Mohalkar						
Simon	Jones						
Alexander	Anderson						
Anthony	Smith						
Sushrut	Kulkarni						
David	Isaac						
William	Bartlett						
Rohit	Rambani						
Benedict	Lankester						
Mark	Andrews						
Shiv	Sha						
Richard	Pilling						
Joseph	Aderinto						
Ram	Venkatesh						
Mark	Emerton						
Campbell	Maceachern						
Jonathan	Lamb						
Ayman	Sorail						
Shoaib	Ahmed						
Edward	Holloway						
Sameer	Jain						
Veysi	Veysi						
Jeya	Palan						
Lutz	Koch						
Francis	Sim						
Andrew	Legg						
Kevin	Wembridge						
Anil	Hormis						
Pete	Gallacher						

Supplemental Online Content: Nonauthor Collaborators

*Indicates required information. Only first name, last name, and suffix will appear in PubMed.

***First Name and Middle Initial(s)**	***Last Name**	***Suffix (eg, Jr, III)**	Academic Degrees	Institution	Location (city, state/province, country)	Role or Contribution, eg, chair, principal investigator	Group (if more than 1 Group listed in the byline) and/or Subgroup (eg, Steering Committee)
Paul	Jermin						
Nikhil	Sharma						
Theo	Boras						
Andrew	Barnett						
Taushaba	Hossain						
Shanaka	Senevirathna						
Omer	Salas						
Vikram	Desai						
Joby	John						
Michael	Hockings						
Talal	Al-Jabri						
Dipak	Raj						
Abdul	Moeed						
Sam	Heaton						
Matthew	Hall						
Paul	Latimer						
Paul	Porter						
Sudhir	Robertson						
Ken	Mannan						
