# Supplementary material for: Efficacy of Liposomal Bupivacaine and Bupivacaine Hydrochloride vs Bupivacaine Hydrochloride Alone as a Periarticular Anesthetic for Patients Undergoing Knee Replacement: A Randomized Clinical Trial
Source: JAMA Surg. 2022 Apr 6;157(6):481–9. doi: 10.1001/jamasurg.2022.0713 (PMC8988023; doi:10.1001/jamasurg.2022.0713)
Supplement: Supplement 4. — Data Sharing Statement [file jamasurg-e220713-s004.pdf]

Data Sharing Statement

Hamilton. Efficacy of Liposomal Bupivacaine and Bupivacaine Hydrochloride vs Bupivacaine Hydrochloride Alone as a Periarticular Anesthetic for Patients Undergoing Knee Replacement. *JAMA Surg*. Published April 06, 2022. doi:10.1001/jamasurg.2022.0713

Data
Data available: Yes
Data types: Deidentified participant data, Data dictionary
How to access data: The data collected for the study, including individual participant data and a data dictionary defining each field in the set, will be made available to researchers on request to the study team and with appropriate reason when accompanied by a peer-reviewed protocol, with publication and on agreement of the Trial Steering Committee. The shared data will be deidentified participant data. Data will be shared with investigator support, after approval of a proposal, with a signed data access agreement. Requests should be directed to the corresponding author.
When available: With publication

Supporting Documents
Document types: Other (please specify)
Additional Information: Trial Protocol Statistical Analysis Plan Health Economic Analysis Plan
How to access documents: Files submitted electronically to be included as Appendices.
When available: With publication

Additional Information
Who can access the data: Files submitted electronically to be included as Appendices.
Types of analyses: The data collected for the study, including individual participant data and a data dictionary defining each field in the set, will be made available to researchers on request to the study team and with appropriate reason when accompanied by a peer-reviewed protocol, with publication and on agreement of the Trial Steering Committee. The shared data will be deidentified participant data. Data will be shared with investigator support, after approval of a proposal, with a signed data access agreement. Requests should be directed to the corresponding author.
Mechanisms of data availability: The data collected for the study, including individual participant data and a data dictionary defining each field in the set, will be made available to researchers on request to the study team and with appropriate reason when accompanied by a peer-reviewed protocol, with publication and on agreement of the Trial Steering Committee. The shared data will be deidentified participant data. Data will be shared with investigator support, after approval of a proposal, with a signed data access agreement. Requests should be directed to the corresponding author.
